# Deciphering resistance mechanisms to auxin-inducible protein degradation in mammalian cells

**DOI:** 10.1016/j.jbc.2026.113232

**Published:** 2026-06-04

**Authors:** Judith Hyle, Zhenling Liu, Shaela Fields, Jifeng Yang, Xinyan Chen, Wenjie Qi, Qiong Zhang, Byoung-Kyu Cho, Young Ah Goo, Xiuling Li, Jack Sublett, Qianqian Li, Liusheng He, Jonathon Klein, Peng Xu, Shondra M. Pruett-Miller, Beisi Xu, Chunliang Li

**Affiliations:** 1 Department of Tumor Cell Biology, St Jude Children's Research Hospital, Memphis, Tennessee, USA; 2 Center for Applied Bioinformatics, St Jude Children's Research Hospital, Memphis, Tennessee, USA; 3 Mass Spectrometry Technology Access Center at the McDonnell Genome Institute, Washington University School of Medicine, St Louis, Missouri, USA; 4 Genetically Engineered Mouse Models Shared Resource, St Jude Children's Research Hospital, Memphis, Tennessee, USA; 5 Flow Cytometry and Cell Sorting Shared Resource, St Jude Children's Research Hospital, Memphis, Tennessee, USA; 6 Center for Advanced Genome Engineering, St Jude Children's Research Hospital, Memphis, Tennessee, USA; 7 Cyrus Tang Medical Institute, National Clinical Research Center for Hematologic Diseases, State Key Laboratory of Radiation Medicine and Protection, Collaborative Innovation Center of Hematology, Soochow University, Suzhou, Jiangsu, PR China

**Keywords:** CTCF, auxin-inducible degron, resistance, genome editing, leukemia

## Abstract

Targeted protein degradation is a favorable strategy for studying the immediate downstream effects of protein loss-of-function, such as the auxin-inducible degron (AID) system. Although this system has been applied extensively to cell and animal models, degradation resistance to long-term auxin treatment has not been studied. With the advent of the new AID2 system, cellular toxicity caused by the high auxin concentrations required in the original AID1 system is no longer a concern, enabling the study of protein degradation over extended periods. In this study, we derived multiple miniAID-tagged knock-in human cell lines and a Ctcf-miniAID knock-in mouse strain to investigate mechanisms of degradation resistance. We revealed four independent resistance mechanisms, including a nonsense mutation in the CTCF coding sequence that removed the miniAID peptide, a missense point mutation in the miniAID coding region that disrupted ubiquitin complex targeting, and reduced expression of OsTIR1 adaptor protein. Resistance to auxin degradation was also observed in mouse primary Ctcf^miniAID/miniAID^ knock-in B-cell acute lymphoblastic leukemia cells through missense mutations of the OsTIR1^(F74G)^ protein *ex vivo* and *in vivo*. By systematically characterizing degron resistance mechanisms in mammalian cells, we identified potential limitations of the AID system for long-term protein degradation studies.

Due to the rapid development of genomic profiling technologies, numerous genes have been identified as essential players in human development and disease progression (1, 2). To dissect the functional roles of these genes, researchers have employed loss-of-function approaches, including shRNA (3, 4), Cre/LoxP (5, 6), and recent genome-editing tools such as CRISPR/Cas9 (7, 8, 9). While these tools are powerful for impairing gene function, they also share common drawbacks. These include a longer time to complete knockdown and off-targeting effects, especially when using DNA- and RNA-level editing techniques. Therefore, novel loss-of-function strategies that harness the cell’s intrinsic machinery to promote the degradation of the protein of interest have emerged as promising approaches to fill the gap (10, 11, 12, 13, 14).

Degrons, small peptide sequences recognized by the E3 ubiquitin ligase, can be engineered to proteins of interest to allow acute protein degradation by the cellular ubiquitin-proteasome system (UPS). Common protein degradation systems include dTAG (14), the PROTAC (proteolysis-targeting chimera) system (11, 13), Bromotag (12), and the auxin-inducible degron (AID) system (15). The AID system stands out because the miniAID tag is the smallest degron at 7 kDa, minimizing the risk of altering native protein structure and function. Although first identified in plants, the AID system was introduced into mammalian cells to efficiently, rapidly, and reversibly degrade a protein of interest (16, 17, 18, 19). Mechanistically, the AID system utilizes auxin (indole-3-acetic acid, IAA) as a molecular glue to bind Aux/IAA proteins to the endogenous UPS, after which the protein is conjugated to ubiquitin complex and targeted for protein degradation within minutes to hours (15). To functionally equip the system in live cells, a 204-bp miniAID sequence containing the highly conserved domain II of Aux/IAA proteins is delivered into the endogenous locus of the targeted gene *via* genome editing, producing a miniAID fusion protein. In addition, the plant-derived F-box adaptor protein, OsTIR1, is ectopically expressed in cells and recruits miniAID-tagged proteins to the UPS in the presence of the auxin ligand. Upon auxin treatment, the ternary UPS complex, including the AID-tagged protein, an E3 ubiquitin ligase, and auxin, immediately organizes and executes protein degradation, which can be reversed upon auxin removal. Over the past decade, the generalizable application of the AID system has been reported across numerous organ systems, including worms, flies, human, and mammalian cell lines and the mouse model *in vivo* (15, 19, 20, 21, 22, 23). A few years ago, the system was upgraded to the AID2 version by generating a mutant form of OsTIR1, OsTIR1^(F74G)^, that specifically binds a synthetic auxin analog, 5-phenyl-IAA (5-Ph-IAA). The AID2 system notably decreased auxin toxicity and increased sensitivity, further optimizing it for studying immediate gene function and long-term (LT) treatment regimens *in vitro* and *in vivo* (19, 22, 23).

As with many of the drug-based approaches, LT drug treatment to induce degradation could lead to resistance (24, 25). Indeed, acquired resistance to PROTAC has been shown to develop through genetic alterations in key components of the UPS complex (26, 27, 28). Because the AID system also relies on the cell’s intrinsic E3 ubiquitin ligase machinery, we hypothesized that LT auxin treatment would enable cells to develop mechanisms to counteract protein degradation. This could lead to drug resistance, particularly for essential genes required for cell survival. To our knowledge, there is a lack of systematic investigation of AID resistance in mammalian systems, hindering our understanding of safety for future applications.

To this end, we have derived multiple human miniAID-protein knock-in cell lines targeting genes defined as survival essential in the acute lymphoblastic leukemia cell line SEM, including *CTCF*, *RBM5*, and *MBNL1*. Upon LT auxin treatment, miniAID-tagged RBM5 and MBNL1 retained auxin sensitivity. Strikingly, the miniAID-tagged CTCF frequently became auxin-resistant in human cell lines. To further explore whether the resistance phenotype is due to *in vitro* culture conditions, we confirmed the resistance phenotype from a newly established Ctcf-miniAID knock-in mouse model. Overall, in the human CTCF^AID2^ SEM cells, three independent resistance mechanisms were discovered (1): a nonsense mutation of the *CTCF* coding sequence to truncate the miniAID peptide (2); a missense point mutation at the *miniAID* coding region to disrupt ubiquitin complex targeting; and (3) epigenetic silencing of the OsTIR1^(F74G)^ adaptor protein. In mouse primary Ctcf^miniAID/miniAID^ knock-in B-cell acute lymphoblastic leukemia (B-ALL) cells, missense mutations of the OsTIR1^(F74G)^ adaptor protein were seen *ex vivo* and *in vivo*. In summary, our comprehensive study has revealed the detailed molecular mechanisms of AID resistance in mammalian cells. These findings significantly expand our understanding of the AID system and encourage careful consideration in the design of future applications.

## Results

### Development and characterization of auxin resistance in CTCF^AID2^ human cell lines

**Figure 1:**
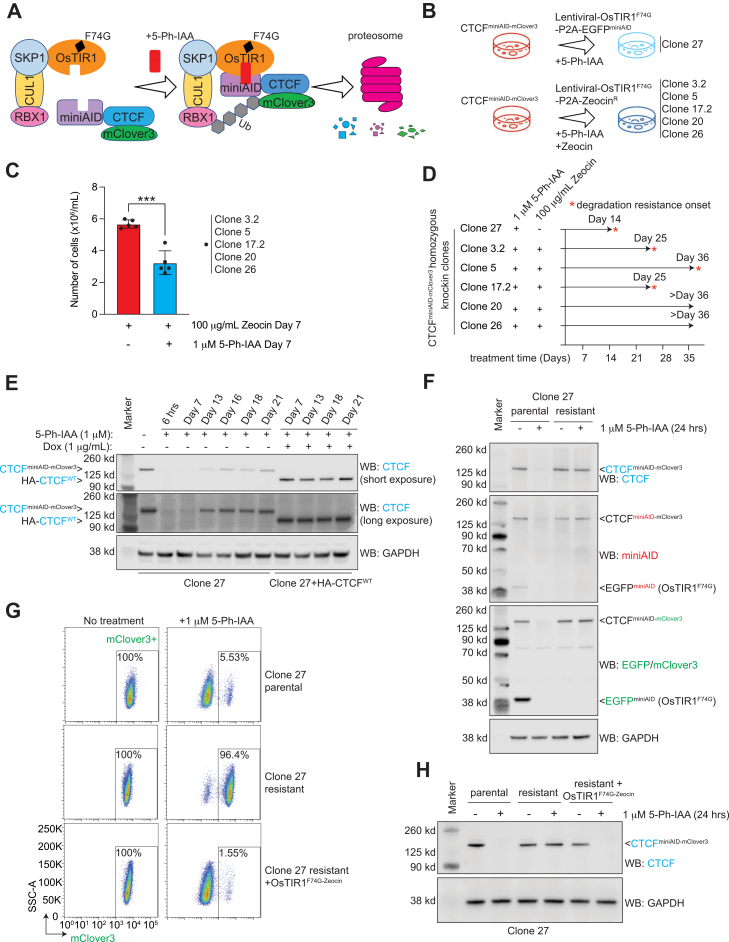
**Exploring auxin-inducible degradation resistance in human cell culture.***A*, schematic diagram detailing how the auxin-inducible system works to degrade miniAID-tagged proteins. The auxin analog, 5-Ph-IAA, acts as a ligand to bind OsTIR1^(F74G)^ to miniAID-mClover3-tagged CTCF. The SCF complex is recruited to bind OsTIR1^(F74G)^. The miniAID-mClover3-tagged CTCF is subsequently ubiquitinated by E3 ligase, and proteasomal cleavage occurs. *B*, diagram depicting the long-term auxin culture conditions for CTCF^AID2^ clones. All clones were grown in suspension cell culture and treated with 1 μM 5-Ph-IAA for up to 36 days. Clones 3.2, 5, 17.2, 20, and 26 were also treated with 100 μg/ml Zeocin to maintain selection pressure for OsTIR1^(F74G)^ expression. *C*, bar graph representing cell counts on day 7 in culture from CTCF^AID2^ clones treated with 100 μg/ml Zeocin only or 1 μM 5-Ph-IAA plus 100 μg/ml Zeocin. Clones were initially plated at the same cell density (1 × 10^6^/ml). Cells were counted on the seventh day of treatment. ∗∗∗*p* value ≤ 0.001 calculated by unpaired Student’s *t* test. N = 5. *D*, timeline representing the observation of auxin resistance in CTCF^AID2^ clones. *E*, immunoblots from long-term 5-Ph-IAA culture of Clone 27 and Clone 27 + HA-CTCF^WT^. All cultures were maintained under continuous 1 μM 5-Ph-IAA treatment. Clone 27 + HA-CTCF^WT^ was also treated with 1 μg/ml doxycycline (Dox) to induce exogenous HA-CTCF^WT^. Lysates were blotted for CTCF, and GAPDH was included as a loading control. The CTCF^miniAID-mClover3^ protein can be distinguished from HA-CTCF^WT^ by its larger molecular mass. *F*, immunoblots of Clone 27 parental and resistant cells with or without 24 h of 1 μM 5-Ph-IAA treatment. CTCF and miniAID immunoblots confirmed that resistant cells were insensitive to auxin treatment. The GFP immunoblot showed that the OsTIR1^(F74G)^-P2A-EGFP^miniAID^ protein was no longer detected in resistant cells. GAPDH was included as a loading control. *G*, flow cytometry analysis of mClover3 fluorescence, which corresponds to the protein expression of CTCF^miniAID-mClover3^, in Clone 27 parental cells, resistant cells, and resistant cells plus OsTIR1^(F74G)^-Zeocin^R^ with or without 1 μM 5-Ph-IAA treatment for 24 h. Percentages of mClover3+ cells were represented in the lower-right quadrant. *H*, immunoblot of CTCF expression in Clone 27 parental cells, resistant cells, and resistant cells plus OsTIR1^(F74G)^-Zeocin^R^ with or without 1 μM 5-Ph-IAA treatment. Restoring OsTIR1^(F74G)^ to resistant cells reestablished auxin-inducible degradation of CTCF. GAPDH was included as a loading control. SCF, SKP1, CUL1, and F-box; IAA, indole-3-acetic acid; 5-Ph-IAA, 5-phenyl-IAA.

We previously engineered the miniAID tag to an essential gene *CTCF* (29), which is required for maintaining genome-wide chromatin architecture organization in the acute lymphoblastic leukemia B-ALL cell line SEM. CTCF^AID2^ cells have a miniAID-mClover3 tag in frame with both alleles of the 3′ end of *CTCF*. The cells exogenously express OsTIR1^(F74G)^, a mutant of OsTIR1 that binds to SKP1 to form the SKP1, CUL1, and F-box protein complex, which promotes ubiquitination and degradation of miniAID-tagged proteins in the presence of the auxin analog 5-Ph-IAA (Fig. 1*A*). In this study, we combined CTCF^AID2^ cells with extended 5-Ph-IAA treatment to determine how the system would respond to LT CTCF depletion (23). The previously designed CTCF^AID2^ cells, designated Clone 27, expressed OsTIR1^(F74G)^ from the lentiviral cassette pCDH-MND-OsTIR1^(F74G)^-P2A-EGFP^miniAID^ (Fig. 1*B*) (23, 30). Additional CTCF^AID2^ clones were newly established by genome editing in SEM cells. Like Clone 27, the new clones included the miniAID-mClover3 tag at the 3′ end of both *CTCF* alleles, but OsTIR1^(F74G)^ was expressed from a lentiviral cassette, pCDH-MND-OsTIR1^(F74G)^-P2A-Zeocin^R^, enabling sustained Zeocin drug selection pressure to maintain OsTIR1^(F74G)^ expression (Fig. S1*A*). These clones were designated Clone 3.2, Clone 5, Clone 17.2, Clone 20, and Clone 26 and used for the LT auxin treatment studies (Fig. 1*B*).

We previously established in CTCF^AID2^ cells that 1 μM 5-Ph-IAA induced complete CTCF degradation 4 h post-treatment and for up to 96 h without changing the media (23). In this study, all six CTCF^AID2^ clones were maintained under continuous 1 μM 5-Ph-IAA treatment for up to 36 days, with media and drugs refreshed every 72 to 96 h. Clones 3.2, 5, 17.2, 20, and 26 were also treated concurrently with 100 μg/ml Zeocin to maintain selection pressure for OsTIR1^(F74G)^ expression. Because homozygous loss of *Ctcf* leads to early embryonic lethality in mice (31), we anticipated that cells would not tolerate LT depletion of CTCF. Indeed, we observed a significant decrease in cell growth on day 7 in the treatment groups receiving both 5-Ph-IAA and Zeocin, compared with the Zeocin-only groups, demonstrating that cellular fitness was affected by continued CTCF loss (Fig. 1*C*). Strikingly, four of the clones, Clones 27, 3.2, 5, and 17.2, eventually recovered through a survival crisis, suggesting that those clones may acquire resistance to auxin-induced degradation of CTCF (Fig. 1*D*).

The initial LT auxin treatment study was performed in Clone 27 and Clone 27 plus doxycycline-inducible exogenous HA-tagged WT-CTCF (Clone 27+HA-CTCF^WT^). The cells were treated continuously with 5-Ph-IAA for 21 days. Doxycycline was concurrently administered to Clone 27+HA-CTCF^WT^ to induce HA-CTCF^WT^ expression. Immunoblotting for CTCF showed that CTCF^miniAID-mClover3^ initially degraded as expected, but the fusion protein expression gradually reappeared after day 13 of drug treatment. When HA-CTCF^WT^ was induced in parallel with treatment, CTCF^miniAID-mClover3^ remained sensitive to auxin (Fig. 1*E*). In the absence of WT-CTCF rescue, the cells adapted to maintain the essential protein CTCF by escaping auxin-induced degradation. These data highlight that CTCF dependency is the major cause of the degradation resistance.

We defined Clone 27 cells that developed resistance to auxin-induced CTCF degradation after 21 days of auxin treatment as “resistant”, and untreated cells as “parental”. First, we investigated whether increasing the 5-Ph-IAA concentration would force degradation. However, even 10-fold more 5-Ph-IAA did not sensitize resistant cells to auxin (Fig. S1*B*). Therefore, the resistance mechanism likely relied on intrinsic cellular regulation. Since the lentiviral cassette carrying OsTIR1^(F74G)^ in Clone 27 also expressed EGFP^miniAID^, we next evaluated whether the degradation machinery maintained its function in resistant cells by examining how EGFP^miniAID^, a nonessential protein, responded to auxin. Parental cells showed that miniAID-tagged CTCF and EGFP degraded after 24 h of 5-Ph-IAA treatment. However, resistant cells showed no EGFP^miniAID^ expression even in untreated cells, suggesting that the cells may have silenced OsTIR1^(F74G)^-P2A-EGFP^miniAID^ expression or that other unknown intrinsic mechanisms were at work (Fig. 1*F*). Real-time quantitative PCR (RT-qPCR) confirmed transcription of *OsTIR1*^*(F74G)*^ was reduced in resistant cells (Fig. S1*C*). Therefore, we reasoned that additional OsTIR1^(F74G)^ would rescue the functionality of the AID system. When resistant cells were transduced with lentiviral OsTIR1^(F74G)^-P2A-Zeocin^R^, the CTCF^miniAID-mClover3^ sensitivity to auxin was completely restored. Resistant cells with OsTIR1^(F74G)^-P2A-Zeocin lost mClover3 fluorescence upon 5-Ph-IAA treatment, comparable to parental cells (Fig. 1*G*), and CTCF^miniAID-mClover3^ was not detectable by immunoblotting (Fig. 1*H*). Collectively, these data support that CTCF protein expression was restored under continuous auxin challenge by silencing an essential component of the degradation system.

To determine whether maintaining selection pressure for OsTIR1^(F74G)^ would affect the auxin-induced degradation of CTCF, LT treatment of 1 μM 5-Ph-IAA and 100 μg/ml Zeocin was administered to CTCF^AID2^ Clones 3.2, 5, 17.2, 20, and 26, which exogenously expressed OsTIR1^(F74G)^-P2A-Zeocin^R^. All clones initially exhibited reduced cellular growth by day 7 of treatment (Fig. 1*C*). Clones 3.2 and 17.2 recovered from the growth restriction around day 25 (Fig. S1*D*), followed by Clone 5. Immunoblot analysis of Clone 5 confirmed that CTCF protein expression was restored (Fig. S1*E*), and the mechanisms underlying the auxin resistance development in Clones 3.2 and 17.2 will be explored in further detail. Clones 20 and 26 did not recover CTCF protein expression over the treatment time course (Fig. S1, *F* and *G*) and maintained a reduced growth rate in culture.

### Mutation of the degron tag was revealed as a genetic mechanism of auxin resistance

**Figure 2:**
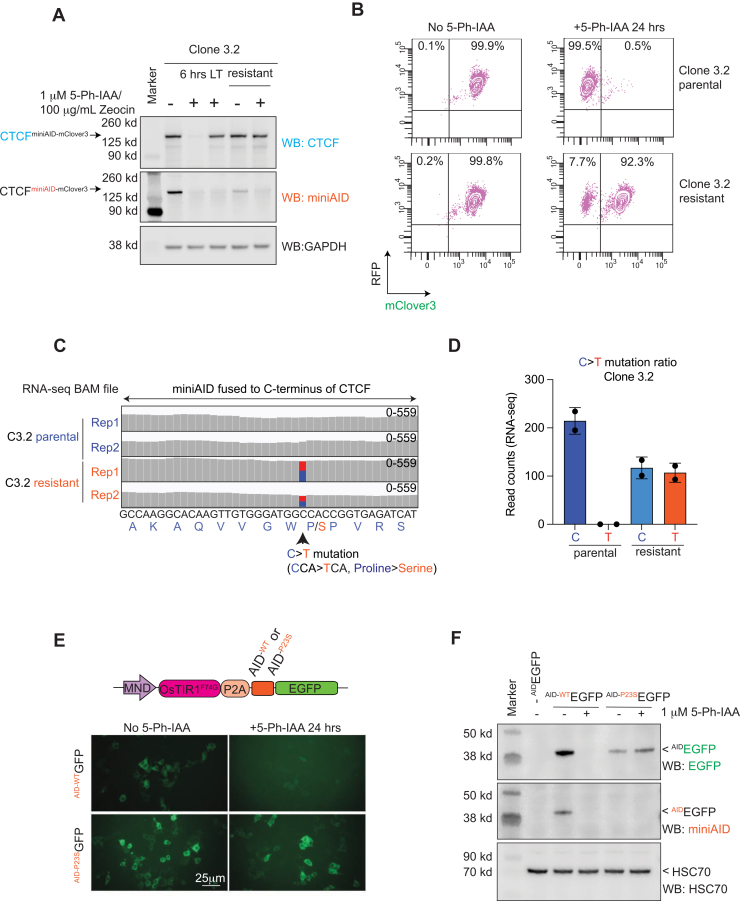
**Genetic mutation of the degron revealed upon long-term auxin treatment.***A*, immunoblots of lysates from Clone 3.2 with and without 1 μM 5-Ph-IAA/100 μg/ml Zeocin treatment for 6 h and 36 days (LT), and LT-treated cells with drugs washed out for 48 h (resistant), also with or without 1 μM 5-Ph-IAA/100 μg/ml Zeocin for 24 h. CTCF immunoblot showed that CTCF^miniAID-mClover3^ became resistant to degradation in LT and in resistant cells. However, the CTCF^miniAID-mClover3^ protein was minimally detectable by miniAID. GAPDH was included as a loading control. *B*, flow cytometry analysis of RFP [OsTIR1(F74G)-P2A-ZeocinR-EF1α-RFP] and mClover3 (CTCF^mini^^AID-mClover3^) fluorescence in Clone 3.2 parental and resistant cells with or without 1 μM 5-Ph-IAA treatment for 24 h. Percentages of RFP+ and mClover3+ cells are represented in the upper-right quadrant. RFP+ and mClover3-cells are represented in the upper-left quadrant. *C*, RNA-seq track of the miniAID cDNA sequence from RNA collected from Clone 3.2 parental and resistant cells showing the C > T mutation at amino acid 23, resulting in a proline to serine missense mutation. The *blue bar* represents the wildtype “C” nucleotide. The *red bar* represents the “T” mutation. *D*, bar graph representing read counts of the C > T mutation ratio in Clone 3.2 parental and resistant cells. The “T” mutation was not detected in parental cells. *E*, fluorescence microscopy of 293T cells transduced with OsTIR1^(F74G)^-P2A-EGFP^miniAID^ carrying either AID^WT^ or AID^P23S^ EGFP. AID^P23S^ EGFP maintained GFP fluorescence after 1 μM 5-Ph-IAA treatment for 24 h in cells expressing ^AIDP23S^EGFP. *F*, immunoblots of lysates from 293T cells transduced with OsTIR1^(F74G)^-P2A-EGFP^miniAID^ carrying either ^AID-WT^EGFP or ^AID-P23S^EGFP following 24 h of 1 μM 5-Ph-IAA treatment. The CTCF immunoblot shows that the ^AID-P23S^EGFP protein was no longer sensitive to auxin-induced degradation, and the miniAID immunoblot reveals that the ^AID-P23S^EGFP protein was no longer detectable by the miniAID antibody. HSC70 was included as a loading control. 5-Ph-IAA, 5-phenyl-IAA; AID, auxin-inducible degron; IAA, indole-3-acetic acid; LT, long-term.

Immunoblotting of Clone 3.2 following 36 days of LT treatment with 5-Ph-IAA and Zeocin showed that the endogenous CTCF^miniAID-mClover3^ became resistant to auxin-induced degradation. When LT-treated cells removed from treatment for 48 h (designated as Clone 3.2 resistant) were rechallenged with 5-Ph-IAA, CTCF^miniAID-mClover3^ remained insensitive to auxin (Fig. 2*A*). Fluorescence of mClover3 was minimally abrogated upon 5-Ph-IAA treatment in resistant cells, demonstrating that the majority of the endogenous CTCF^miniAID-mClover3^ protein was resistant to auxin (Fig. 2*B*). Although the CTCF^miniAID-mClover3^ protein was detectable by immunoblotting for CTCF in LT- and resistant-treated cells, it was minimally detected by the AID antibody in resistant cells, suggesting that the AID epitope might be blocked (Fig. 2*A*). To further examine this observation, we conducted RNA-seq analysis in Clone 3.2 parental (untreated) and resistant cells and detected a C > T mutation that resulted in a proline-to-serine missense mutation at amino acid 23 (P23S) of the miniAID tag (Fig. 2, *C* and *D*). To confirm that the P23S mutation could lead to auxin resistance, the AID on the lentiviral OsTIR1^(F74G)^-P2A-EGFP^miniAID^ cassette was mutated to express the P23S-AID. Then, 293T cells were transiently transfected with either the ^AID-WT^EGFP or ^AID-P23S^EGFP lentiviral construct and challenged with 5-Ph-IAA treatment for 24 h. Cells expressing ^AID-WT^EGFP demonstrated acute loss of EGFP fluorescence after 5-Ph-IAA treatment. However, ^AID-P23S^EGFP expressing cells maintained EGFP fluorescence following treatment (Fig. 2*E*). Immunoblot analysis further confirmed that ^AID-P23S^EGFP was no longer degradable by auxin treatment. In addition, the AID antibody no longer detected ^AID-P23S^EGFP, indicating that the mutation disrupted the antibody–epitope interaction (Fig. 2*F*). The LT treatment was repeated in parental Clone 3.2 to determine whether auxin resistance was reproducible. Indeed, auxin-resistant CTCF^miniAID-mClover3^ appeared around day 25 of treatment, and Sanger sequencing confirmed the P23S-AID mutation in cDNA and genomic DNA from cells treated for 34 days (Fig. S2, *A*–*C*). Collectively, these data support that based on the selection pressure, a genomic mutation occurred in the CTCF^miniAID-mClover3^ miniAID-tag that impaired the function of miniAID and retained the expression and function of CTCF in cells.

### Missense mutation Q666∗ in CTCF caused early truncation to exclude the degron tag and escape auxin degradation

**Figure 3:**
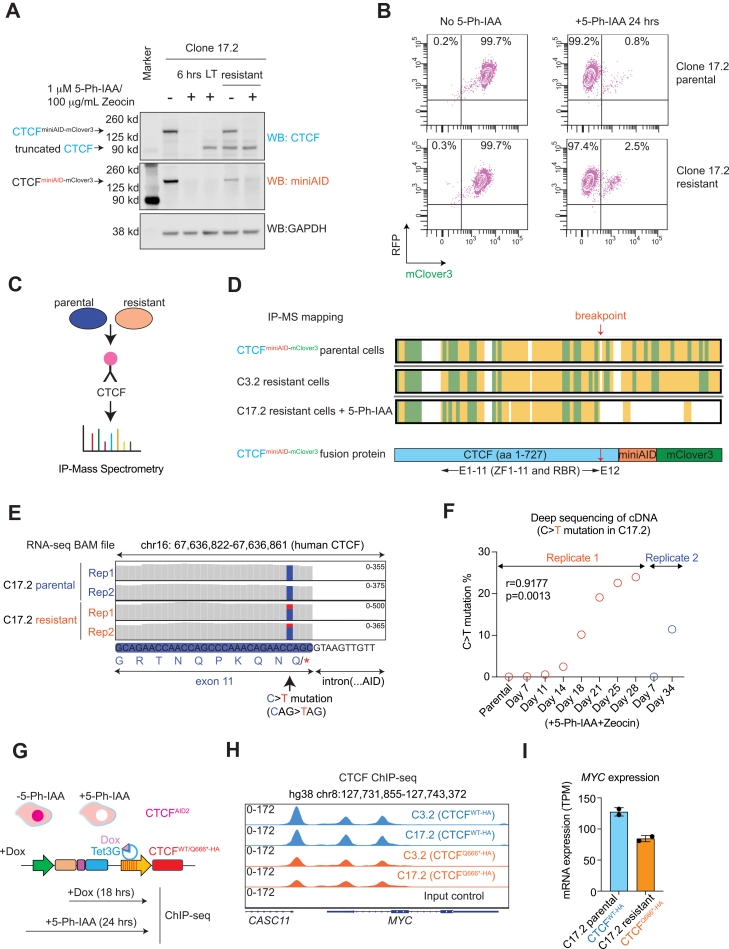
**Genetic mutation of CTCF revealed upon long-term auxin treatment.***A*, immunoblots of lysates from Clone 17.2 with or without 1 μM 5-Ph-IAA/100 μg/ml Zeocin treatment for 6 h and 36 days (LT), and LT-treated cells with drugs washed out for 48 h (resistant), also with or without 1 μM 5-Ph-IAA/100 μg/ml Zeocin for 24 h. CTCF immunoblot showing CTCF^miniAID-mClover3^ remained sensitive to degradation in LT and resistant cells. However, a truncated CTCF form was detected in LT-treated cells. The miniAID antibody did not detect the truncated CTCF protein. GAPDH was included as a loading control. *B*, flow cytometry analysis of RFP [OsTIR1^(F74G)^-P2A-ZeocinR-EF1α-RFP] and mClover3 (CTCF^miniAID-mClover3^) fluorescence in Clone 17.2 parental and resistant cells with or without 1 μM 5-Ph-IAA treatment for 24 h. Percentages of RFP+ and mClover3+ cells are represented in the upper-right quadrant. RFP+ and mClover3-cells are represented in the upper-left quadrant. *C*, schematic diagram of CTCF immunoprecipitation and mass spectrophotometry of parental and resistant CTCF^AID2^ clones 3.2 and 17.2. *D*, sequence bar representing mass spectrometry peptide sequence coverage of the CTCF^miniAID-mClover3^ protein. *Yellow* and *green* shading highlight peptides detected with high confidence. *Green* shading predicts peptides with possible posttranslational modifications. The CTCF^miniAID-mClover3^ bar represents the CTCF amino acid sequence (*blue*), miniAID tag (*orange*), and the mClover3 tag (*green*). The *red arrow* indicates the breakpoint. *E*, RNA-seq track of the exon 11 cDNA sequence from RNA collected from Clone 17.2 parental and resistant cells showing the C > T mutation at amino acid 666, resulting in a glutamine to stop codon nonsense mutation. The *blue bar* represents the WT “C” nucleotide. The *red bar* represents the “T” mutation. *F*, chart representing the deep sequencing readout percentage of the C > T mutation’s emergence in cDNA throughout continuous treatment with 1 μM 5-Ph-IAA/100 μg/ml Zeocin for up to 34 days in Clone 17.2. Two replicates were included. *G*, schematic diagram of the model to switch from endogenous CTCF expression to doxycycline-inducible exogenous CTCF^WT-HA^ and CTCF^Q666∗-HA^ expression. *Green arrow* = MND promotor; *pink rectangle* = Zeocin resistance; *purple rectangle* = P2A; *blue rectangle* = Tet-on 3G; *orange arrow* = TRE3G promoter; *red rectangle* = CTCF (either WT or Q666∗). CTCF^AID2^ cells were transduced to express either CTCF^WT-HA^ or CTCF^Q666∗-HA^. First, cells were treated with 1 μM 5-Ph-IAA for 24 h. After 6 h of treatment, doxycycline (Dox) was added to induce exogenous CTCF expression. *H*, CTCF ChIP seq tracks of CTCF^AID^^2-WT^^-HA^ and CTCF^AID^^2-Q^^666∗-HA^ clones (C3.2 and C17.2) at the *MYC* locus showed increased CTCF binding in CTCF^AID^^2-WT^^-HA^ clones. *I*, reduced expression (TPM) of *MYC* observed in C17.2-resistant cells that express the truncated CTCF^Q666∗^ protein when compared to C17.2 parental cells that express the full-length CTCF^WT^. *p*-value ≤ 0.05 calculated by unpaired Student’s *t* test. 5-Ph-IAA, 5-phenyl-IAA; E, exon; AID, auxin-inducible degron; IAA, indole-3-acetic acid; IP-MS, immunoprecipitation and mass spectrophotometry; LT, long-term; RBR, RNA binding domain; ZF, zinc finger.

Unlike Clone 3.2, immunoblotting of Clone 17.2 following LT treatment (36 days) with 5-Ph-IAA and Zeocin showed that the endogenous CTCF^miniAID-mClover3^ band remained sensitive to auxin degradation. However, the emergence of a smaller truncated band was observed (Fig. 3*A*). Although the CTCF antibody detected the truncated band, it was not detectable by the AID antibody, suggesting that the protein had undergone a C-terminus truncation. Auxin and Zeocin were washed out of LT-treated cells after 48 h, and the cells were defined as resistant. Following wash-out, resistant cells were re-challenged with 5-Ph-IAA and Zeocin for 24 h. Immunoblotting showed that the endogenous CTCF^miniAID-mClover3^ band was restored without auxin and remained sensitive to auxin treatment. The truncated CTCF band that developed during the LT treatment persisted following washout (Fig. 3*A*). Fluorescence analysis also confirmed that mClover3 fluorescence was reduced in Clone 17.2-resistant cells following auxin treatment, further demonstrating the sensitivity of endogenous CTCF^miniAID-mClover3^ to auxin-induced degradation (Fig. 3*B*). To systematically map and characterize the truncated CTCF, CTCF antibody-based immunoprecipitation and mass spectrophotometry (IP-MS) were performed on parental (untreated) and Clone 17.2-resistant cells treated with 5-Ph-IAA to remove the endogenous CTCF^miniAID-mClover3^. Clone 3.2-resistant cells were also included. While IP-MS captured peptides covering the entire sequence of CTCF^miniAID-mClover3^ in both parental and Clone 3.2-resistant cells, Clone 17.2-resistant cells displayed an abridged sequence for CTCF^miniAID-mClover3^, with a likely breakpoint occurring before exon 12 that removed the 3′ end of CTCF along with the miniAID-mClover3 tag (Figs. 3, *C*, *D* and S3*A*). RNA-seq further confirmed a C > T mutation that resulted in a nonsense mutation of glutamine to a stop codon at the amino acid 666 (Q666∗) in exon 11 (Figs. 3*E* and S3*B*). LT treatment of Clone 17.2 was performed independently for two additional replicates to determine whether the truncated CTCF would stably appear upon auxin challenge. Deep sequencing confirmed that the C > T mutation emerged in conjunction with the expression of the truncated CTCF protein, CTCF^Q666^∗ (Figs. 3*F* and S3, *C*, *D*). Ultrasensitive sequencing did not detect the Q666∗ mutation in parental populations at levels above background, suggesting that the mutation developed in culture in response to the auxin challenge (Fig. S3*E*). Notably, pan-cancer genome and transcriptome analysis of pediatric malignancies identified a splice region and frameshift mutation at P667 at the E11/E12 junction of *CTCF* in cases of AML and neuroblastoma, respectively, supporting the notion that this region of the gene is predisposed to genetic instability (32).

To determine if the truncated CTCF^Q666^∗ would recapitulate CTCF^WT^ function, we compared the DNA-binding affinity of CTCF^Q666^∗ to CTCF^WT^ by ChIP-seq. Since the truncated CTCF arose during LT culture under a drug challenge, alterations in CTCF-DNA binding could not be distinguished from culture stress. Therefore, an inducible expression cell model was developed to ectopically express CTCF^WT^ or CTCF^Q666^∗ upon doxycycline treatment. CTCF^AID2^ clones 5, 3.2, and 17.2 were transduced to express doxycycline-inducible and HA-tagged CTCF^WT^ or CTCF^Q666^∗. Cells were treated for 24 h with 1 μM 5-Ph-IAA to degrade endogenous CTCF. Six hours into treatment, doxycycline was added to induce exogenous CTCF expression (Fig. 3*G*). A doxycycline titration was performed to induce comparable expression between CTCF^AID2-WT-HA^ and CTCF^AID2-Q666^∗^-HA^ cells, as determined to be 0.05 μg/ml and 1.0 μg/ml doxycycline, respectively (Fig. S4*A*). CTCF ChIP-seq isolated the DNA regions bound to CTCF-binding sites in either CTCF^WT-HA^ or CTCF^Q666^∗^-HA^ settings. Principal component analysis confirmed that the reproducible variation among the samples was due to CTCF mutation (Fig. S4*B*). Global CTCF binding was maintained in all groups, as expected, since the core zinc finger DNA-binding domain remained intact in the CTCF^Q666^∗ mutant. Peaks called with the least change in binding (control) exhibited weaker binding at CTCF motifs (Fig. S4*C*), with ∼ 40% binding at regions without a CTCF consensus motif, which could be attributed to cofactor binding (Fig. S4*D*). In 7727 peaks, CTCF binding was greater in CTCF^AID2-WT-HA^ cells compared to CTCF^AID2-Q666^∗^-HA^ cells [log2 FC > 1, false discovery rate (FDR) < 0.05], with more than 90% enrichment at regions containing the CTCF consensus motif (Fig. S4, *E* and *F*). Binding was reduced at only 59 peaks in CTCF^AID2-WT-HA^ cells compared to CTCF^AID2-Q666^∗^-HA^ cells (log2 FC > 1, FDR < 0.05). Motif enrichment analysis confirmed the CTCF binding motif as the most enriched motif among the 7727 increased binding peaks (Fig. S4*G*). The *MYC* locus, which is positively regulated by CTCF, showed decreased CTCF binding and transcription in CTCF^AID2-Q666^∗^-HA^ cells (Fig. 3, *H* and *I*). Also, at the *BLCAP* locus where CTCF acts as an insulator of transcription, CTCF binding decreased in CTCF^AID2-Q666^∗^-HA^ cells. However, most loci showed equal CTCF binding (Fig. S4*H*). Taken together, upon selection pressure in culture, the CTCF^miniAID-mClover3^ miniAID-tag was lost by the genetic mutation of an early stop codon in CTCF that retained the near-functional peptide fraction of CTCF at a genome-wide scale.

### Auxin resistance is selective and requires survival pressure

To further extend our study of degron resistance to other proteins, we used a similar genome-editing approach [a mixture of Cas9 protein, single-guide RNA (sgRNA), and a knock-in donor plasmid] to deliver the miniAID tag to *RBM5* and *MBNL1*, two genes previously identified as essential in the SEM cell line (33, 34). ^HA-miniAID^RBM5 and MBNL1^miniAID- HA^ knock-in clones were derived in SEM cells, and knock-in cells were transduced with lentiviral-OsTIR1^(F74G)^-P2A-Zeocin^R^. Cells were grown in culture with concurrent treatment with 1 μM 5-Ph-IAA and 100 μg/ml Zeocin for up to 23 days. Interestingly, reduced proliferation was not observed in either cell line. Immunoblotting of the fusion proteins by protein-specific and HA-tag antibodies following LT treatment (>23 days) with 5-Ph-IAA and Zeocin showed that the endogenous ^HA-miniAID^RBM5 and MBNL1^miniAID-HA^ proteins remained sensitive to auxin degradation over the treatment time course (Fig. S5, *A* and *B*). These data suggest that auxin resistance was selective and less likely to be initiated without selection pressure.

### Establishment and validation of the Ctcf^miniAID/miniAID^ BCR-ABL B-ALL mouse model

**Figure 4:**
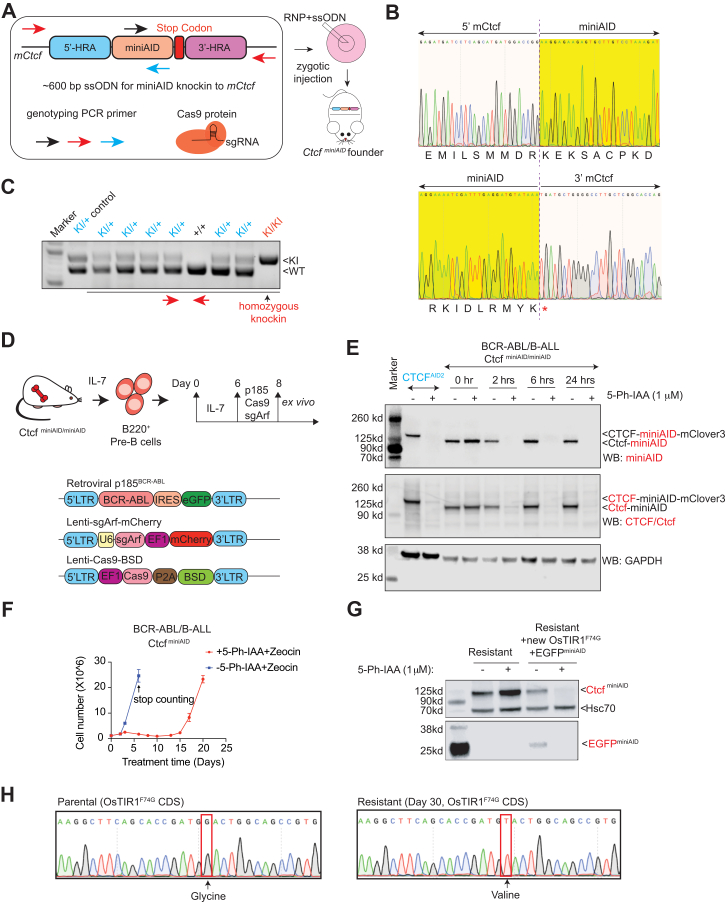
**Establish and characterize the CtcfminiAID/miniAID mouse strain and CtcfminiAID/miniAID BCR-ABL B-ALL model for studying acute and long-term response to auxin-induced degradation.***A*, schematic diagram showing the 600 bp single-stranded oligodeoxynucleotides (ssODN) with 200-bp 5′ and 3′ homology recombinant arms (5′-HRA and 3′-HRA) and a 204-bp miniAID sequence. The *arrows* indicate primer positions for genotyping PCR. The ribonucleotide complex (RNP) is formed by the Cas9 protein and the sgRNA against Ctcf *in vitro*. The ssODN was mixed with the RNP complex, followed by a zygotic injection to generate genome editing and establish the Ctcf^miniAID^ knock-in founder mice. *B*, Sanger sequencing results confirm the junction sequence of 5′ and 3′ miniAID in the same reading frame with Ctcf before the stop codon. The *top* row shows the reflective codon readout of the 3′ end of Ctcf switching to the 5′ miniAID codon sequence. The second row illustrates the 3′ end of the miniAID tag with a stop codon transitioning back to the Ctcf endogenous sequence. *C*, genotyping PCR results confirm homozygous Ctcf^miniAID/miniAID^ among wildtype and heterozygous offspring. *Red arrows* indicate that the genotyping PCR primers used sat outside the homology arms. *D*, schematic diagram describing the procedure to derive the primary cell model from the homozygous Ctcf^miniAID/miniAID^ knock-in animal. Bone marrow samples from 8-week-old mice were collected and cultured with BCM5 and IL-7 conditional medium for 6 to 8 days to maintain pre-B cells, followed by transformation with retroviral-BCR-ABL and lentiviral-Cas9, as well as lentiviral-sgArf to transform pre-B and obtain the BCR-ABL B-cell acute lymphoblastic leukemia (B-ALL) model. *E*, immunoblot of Ctcf and miniAID expression with or without 1 μM 5-Ph-IAA and 50 μg/ml Zeocin treatment for 0 h (0 h), 2 h (2 h), 6 h (6 h), or 24 h (24 h). CTCF^AID2^ cells with or without 5-Ph-IAA treatment were used as controls. Upon 5-Ph-IAA treatment, the Ctcf-miniAID fusion protein was acutely degraded as early as 2 h post 5-Ph-IAA treatment. GAPDH served as the loading control. *F*, cell counting was conducted for Ctcf^miniAID/miniAID^ BCR-ABL B-ALL cells with and without long-term treatment of 1 μM 5-Ph-IAA and 50 μg/ml Zeocin. Replication = 3. *G*, resistant cells transduced with additional lentiviral OsTIR1^(F74G)^-EGFP-miniAID restored the Ctcf^miniAID^ sensitivity to auxin treatment, shown by immunoblotting against Ctcf and EGFP (internal control). HSC70 served as the loading control. *H*, sanger sequencing confirmed the missense mutation (G to T) in the OsTIR1^(F74G)^ CDS, encoding an amino acid change from GGA (glycine) to GTA (valine). 5-Ph-IAA, 5-phenyl-IAA; AID, auxin-inducible degron; IAA, indole-3-acetic acid.

Given that all degron resistance experiments were conducted in human cell lines known to be susceptible to clonal adaptation and variation, we explored whether auxin resistance would occur in mouse primary cells, which more accurately represent *in vivo* conditions. As it is impossible to deliver the miniAID tag to both alleles of the endogenous locus in mouse primary cells, we engineered a new Ctcf-miniAID knock-in mouse model that enables the derivation of primary cells from any tissue for degradation assays. To this end, gene editing was used to deliver a mixture of Cas9 protein, an sgRNA against *Ctcf* near the stop codon, and a 600-bp single-stranded oligodeoxynucleotide carrying *Ctcf* homology recombinant arms and the miniAID fragment to mouse zygotes by microinjection (Fig. 4*A*). As a result, three founder mice with successful knock-in were obtained. Sanger sequencing confirmed the seamless knock-in of miniAID in frame with *Ctcf* before the stop codon (Figs. 4*B* and S6*A*). One male founder mouse was bred for germline transmission and produced viable offspring with homozygous Ctcf^miniAID/miniAID^ alleles (Figs. 4*C* and S6*B*). To determine the Ctcf-miniAID fusion protein expression in the knock-in animals, liver, spleen, and kidney tissues were collected from wildtype, heterozygous, and homozygous mice, followed by immunoblotting with the Ctcf antibody. As expected, all tissues expressed the Ctcf-miniAID fusion protein with a molecular weight 7 kDa higher than that of the wildtype Ctcf protein (Fig. S6*C*).

To derive a primary cell model from the homozygous Ctcf^miniAID/miniAID^ knock-in animal, we collected bone marrow from an 8-week-old animal to isolate pre-B cells. The pre-B cells were subsequently cultured with B-cell medium supplemented with IL-7, followed by transformation with retroviral-BCR-ABL (GFP+), lentiviral-Cas9, and lentiviral-sgArf (mCherry+), leading to the establishment of the classic double-positive BCR-ABL B-ALL model (Figs. 4*D* and S6*D*) (35, 36). These Ctcf^miniAID/miniAID^ BCR-ABL B-ALL cells exhibited a fast proliferation rate and sensitivity to the second-generation tyrosine kinase inhibitor Dasatinib, both classic signatures of BCR-ABL transformed cells (Fig. S6, *E* and *F*). They also demonstrated classic features of pre-B cells, including high expression levels of B220 and negative expression of IgM (Fig. S6*F*). The established BCR-ABL Ctcf^miniAID/miniAID^ cells were then transduced with lentiviral OsTIR1^(F74G)^-P2A-Zeocin^R^. Upon 5-Ph-IAA treatment, the Ctcf-miniAID fusion protein was acutely degraded as early as 2 h posttreatment, and expression was restored after auxin removal (Figs. 4*E* and S6*G*).

*Ctcf* was considered an essential gene for survival in almost all cell types. To confirm its role in our Ctcf^miniAID/miniAID^ BCR-ABL B-ALL cells, a competitive proliferation assay was conducted by infecting cells with lentiviral-Cas9 and three sgRNAs against the coding exons of *Ctcf*. All guide RNAs were cloned into a lentiviral cassette with CFP fluorescence. A flow cytometry analysis tracing the CFP fluorescence profile of cells over 8 days showed that cellular fitness depends on the positive control *Myc* and *Ctcf*, supporting *Ctcf* as essential for survival (Fig. S6, *H* and *I*). To explore whether resistance during LT Ctcf^miniAID^ degradation would arise due to a survival crisis, a LT treatment of 1 μM 5-Ph-IAA and 50 μg/ml Zeocin was administered to the cell culture. Cells initially exhibited a dramatic growth crisis immediately upon treatment, then recovered around day 14, with a similar expansion rate compared with parental cells thereafter (Fig. 4*F*). Meanwhile, the resistant cells remained sensitive to dasatinib (Fig. S7*A*). Immunoblotting analysis confirmed that full-length Ctcf protein expression was restored in resistant cells (Fig. S7*B*). Because RNA-seq analysis failed to identify any genetic mutations in the *Ctcf* or *miniAID* sequences, we hypothesized that the resistance mechanism could be due to mutations in the adaptor protein, OsTIR1^(F74G)^. Indeed, reintroducing lentiviral OsTIR1^(F74G)^-P2A-EGFP^miniAID^ into the cells restored the AID system’s functionality (Fig. 4*G*). Sanger sequencing of *OsTIR1*^*(F74G*)^ confirmed a G142V missense mutation (Fig. 4*H*).

To determine if Ctcf^miniAID/miniAID^ BCR-ABL B-ALL cells could potentiate leukemia *in vivo*, 100,000 Ctcf^miniAID/miniAID^ BCR-ABL B-ALL cells were injected into CD1 nude mice (Fig. S7*C*). Flow cytometry analysis of peripheral blood showed increased peripheral leukemic load (Fig. S7*D*). A moribund phenotype typical of leukemia onset was observed 2 to 3 weeks postinjection. Spleen from sacrificed mice showed a significant increase in weight and size compared to the negative control (Fig. S7, *E* and *F*). Hematoxylin and eosin (H&E) staining confirmed morphological abnormalities in the spleen of sick mice *versus* control, and immunohistochemistry (IHC) staining showed abundant GFP-positive leukemia cells in the spleen samples from sick mice (Fig. S7, *G* and *H*). These data confirmed the *in vivo* leukemogenic potential of the Ctcf^miniAID/miniAID^ BCR-ABL B-ALL engrafted cells.

### In vivo modeling of auxin resistance

**Figure 5:**
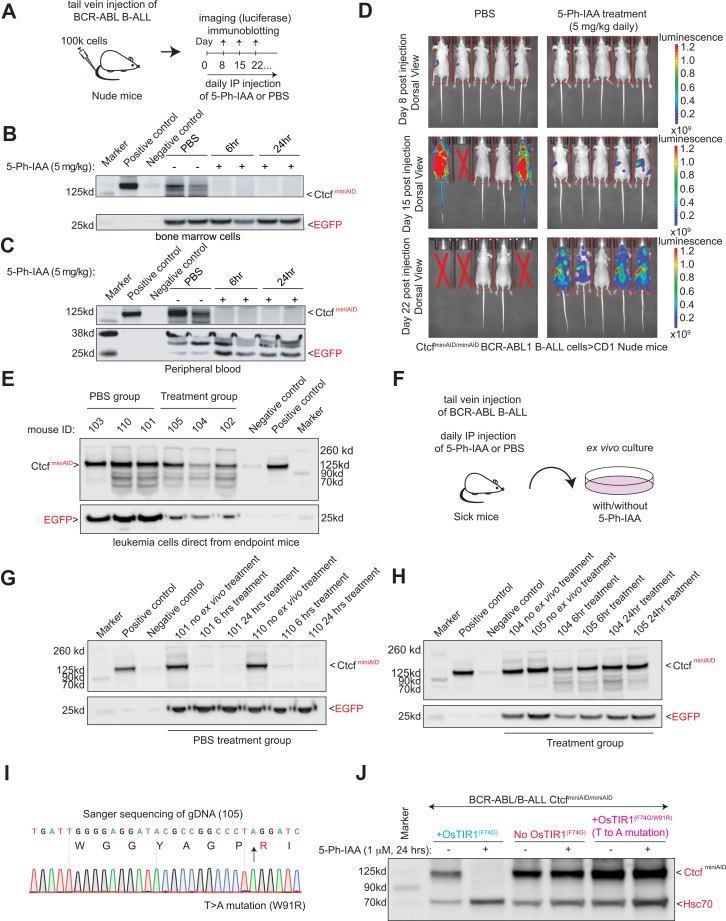
**Long-term *in vivo* treatment with 5-Ph-IAA induced auxin resistance.***A*, schematic diagram summarizing *in vivo* cell injection and 5-Ph-IAA treatment. 100 K Ctcf^miniAID/miniAID^ BCR-ABL B-ALL cells were injected into the nude mice through tail-vein injection. Daily IP injections of 5-Ph-IAA (5 mg/kg) or PBS were administered from the second day after cell injection. *B*, to determine how degradation efficacy *in vivo*, Ctcf^miniAID/miniAID^ BCR-ABL B-ALL cells were injected into CD1 Nude female mice, followed by a 2-week expansion in host mice. Mice confirmed to have leukemia burden in their peripheral blood were then injected with PBS or 5-Ph-IAA. After cell injection and one dose of 5-Ph-IAA treatment, bone marrow cells were collected at 6-h and 24-h time points. Immunoblotting of miniAID confirmed degradation *in vivo*. EGFP was included as a loading control. Ctcf^miniAID/miniAID^ BCR-ABL B-ALL cells without luciferase-YFP transduction ± 5-Ph-IAA treatment were used as positive and negative controls. PBS administration was performed for 24 h as the treatment control. *C*, after cell injection and one dose of 5-Ph-IAA treatment, peripheral blood was collected at 6-h and 24-h time points. Immunoblotting of miniAID confirmed degradation *in vivo*. EGFP was included as a loading control. Ctcf^miniAID/miniAID^ BCR-ABL B-ALL cells without luciferase-YFP transduction ± 5-Ph-IAA treatment were used as positive and negative controls. PBS administration was performed for 24 h as the treatment control. *D*, weekly luciferase imaging of the PBS vehicle group and the 5-Ph-IAA (5 mg/kg) treatment group. On day 15, three-fifths mice in the vehicle group showed abundant leukemic burden, whereas the treatment group showed minimal leukemic burden. On day 22, mice in the 5-Ph-IAA (5 mg/kg) treatment group demonstrated a high leukemic burden. *E*, immunoblot analysis of miniAID and EGFP expression from the leukemia cells collected directly from endpoint mice. Both the PBS vehicle group and treatment group maintained Ctcf-miniAID fusion protein expression. *F*, schematic diagram of *ex vivo* culture of leukemia cells from sick mice. Cells were cultured *ex vivo* and subjected to 5-Ph-IAA treatment (1 μM) to confirm Ctcf degradation. *G*, immunoblotting analysis of miniAID and EGFP expression after *ex vivo* treatment of the PBS vehicle group confirmed Ctcf degradation after 6 h and 24 h of 5-Ph-IAA treatment. *H*, immunoblotting analysis of miniAID and EGFP expression after *ex vivo* treatment of the 5-Ph-IAA treatment group confirmed degradation resistance after 6-h and 24-h 5-Ph-IAA treatment. Ctcf^miniAID/miniAID^ BCR-ABL B-ALL cells without luciferase-YFP transduction ± 5-Ph-IAA treatment were used as positive and negative controls. *I*, Sanger sequencing of the resistant cells confirmed the missense mutation (T to A) in the OsTIR1^(F74G)^ CDS to switch to OsTIR1^(F74G/W91R)^. *J*, a lentiviral construct expressing the OsTIR1^(F74G/W91R)^ mutant was infected into the Ctcf^miniAID/miniAID^ BCR-ABL/B-ALL cells. Auxin treatment was administered to three groups of cells: wildtype OsTIR1^(F74G)^, no OsTIR1^(^^F74G)^, and the mutant form. Immunoblotting was conducted using specific antibodies against Ctcf and Hsc70. 5-Ph-IAA, 5-phenyl-IAA; AID, auxin-inducible degron; B-ALL, B-cell acute lymphoblastic leukemia; IAA, indole-3-acetic acid.

To determine if auxin resistance would occur *in vivo*, Ctcf^miniAID/miniAID^ BCR-ABL B-ALL cells were transduced with MND-luciferase-P2A-YFP. Injecting luciferase-expressing leukemia cells into 10-12-week-old CD1 nude female mice *via* the tail vein enables bioluminescence imaging of leukemia progression after intraperitoneal (IP) injection of luciferin. Before transplantation, the cells were challenged with 5-Ph-IAA to confirm sensitivity to auxin (Fig. S7*I*). After leukemia cells were transplanted, daily IP injections of PBS or 5-Ph-IAA were administered from the second day (Fig. 5*A*). Before LT treatment, a pilot experiment was conducted in mice with leukemia burden to test the drug's efficacy *in vivo*. To this end, blood and bone marrow cells were collected at 6 and 24 h after the first IP injection of drug or PBS, and acute auxin sensitivity was confirmed by Ctcf^miniAID/miniAID^ depletion *in vivo* (Fig. 5, *B* and *C*). Thereafter, the LT treatment of 5-Ph-IAA was carried out by daily IP injection to maintain the degradation pressure. Leukemia load was monitored by daily observation and weekly luciferase imaging. In the PBS/vehicle control group, three out of five mice exhibited successful engraftment of Ctcf^miniAID/miniAID^ BCR-ABL B-ALL cells, with two-fifths expressing high luciferase levels by day 15 postinjection. One mouse from the vehicle group had to be sacrificed by this time point due to high leukemic load, and the remaining two were sacrificed by day 22 after injection. In contrast, the mice administered 5-Ph-IAA showed a notably delayed onset of luminescence. However, four out of five eventually developed a significant leukemic burden (Fig. 5*D*). At the endpoint (approximately 3 weeks after transplantation), peripheral blood, bone marrow, and spleen cells were collected immediately and combined in both the vehicle and treatment groups. Immunoblotting against these fresh samples confirmed that Ctcf^miniAID^ had escaped degradation in the mice treated LT with 5-Ph-IAA (Fig. 5*E*).

### OsTIR1^(F74G)^ mutation led to auxin resistance in vivo

To further determine whether the resistant phenotype could be maintained, leukemia cells collected from the peripheral blood, bone marrow, and spleen of sick mice were combined and cultured *ex vivo*. The *ex vivo* culture was challenged with 5-Ph-IAA treatment (1 μM) for 6 and 24 h (Fig. 5*F*). The vehicle group cells remained sensitive to 5-Ph-IAA treatment. In contrast, the treatment group cells exhibited resistance to auxin degradation at 6 and 24 h after 5-Ph-IAA treatment, confirming that the cells had become resistant to auxin over the LT *in vivo* treatment (Fig. 5, *G* and *H*). Sanger sequencing of the resistant cells confirmed the T > A missense mutation (W91R) in the OsTIR1^(F74G)^ adaptor protein (Fig. 5*I*). Strikingly, resistant cells from three individual mice carried the same point mutation. Site-directed mutagenesis recreated the W91R mutation in the lentiviral OsTIR1^(F74G)^-P2A-Zeocin expression vector, which was transduced to Ctcf^miniAID/miniAID^ BCR-ABL B-ALL cells. Ctcf was not sensitive to auxin-induced degradation in cells expressing the OsTIR1^(F74G/W91R)^ protein (Fig. 5*J*). These data support that under survival-related selection pressure, a genetic mutation in a crucial component of the degron system conferred auxin resistance to preserve expression of the survival-essential protein, Ctcf.

**Figure 6:**
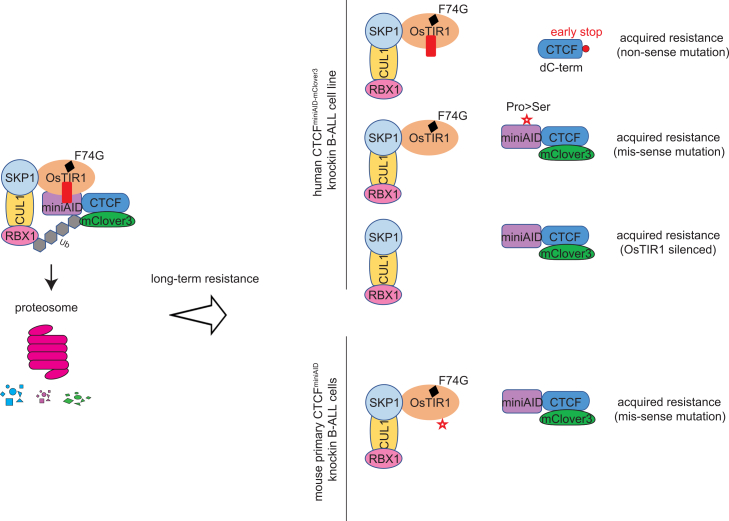
**Schematic diagram summarizing the auxin-induced degron system and the auxin resistance mechanisms observed.** In human CTCF^AID2^ SEM cells: (1) a nonsense mutation of the CTCF coding sequence to truncate the miniAID peptide; (2) a missense point mutation at the miniAID coding region to dissociate the E3 complex targeting; and (3) reduced expression of the OsTIR1^(F74G)^ adaptor protein. In mouse primary Ctcf^miniAID/miniAID^ knock-in B-ALL cells, a missense mutation in the OsTIR1^(F74G)^ adaptor protein was identified. B-ALL, B-cell acute lymphoblastic leukemia.

In summary, we have investigated the mechanisms underlying resistance that developed during prolonged auxin-induced degradation using knock-in cell lines and animal models. We revealed various genetic mutations and possible epigenetic silencing with an unknown intrinsic mechanism underlying auxin resistance (Fig. 6). This innovative study significantly extends our understanding of the AID system, underscoring the need for continued efforts to mitigate resistance.

## Discussion

Targeted protein degradation has recently emerged as an appealing approach to study the immediate effects of protein loss-of-function and continues to evolve in functional genomics research. Recently, proteome-scale AID libraries were developed in *Saccharomyces cerevisiae,* allowing proteome-wide, high-throughput screening of any yeast protein (37, 38) and enabling dynamic functional studies. However, this technology is not feasible in mammalian cells, as homology-directed repair is less efficient than in yeast. Advances in degron targeting, such as the use of single-chain AID2 antibodies (scAb-AID2) to degrade endogenous target proteins, are a step forward toward the therapeutic applicability of AID technology (39). However, limitations such as the need to express OsTIR1^(F74G)^ prevent the use of scAb-AID2 in clinical settings. For now, the AID system remains a key tool in preclinical studies for functionally characterizing proteins and validating targets for future therapeutic potential.

While the AID system has primarily been used for acute protein degradation, studying LT protein loss reveals not only resistance mechanisms but also pathway rewiring and compensatory survival strategies that would be missed in acute depletion studies. Additionally, sustained protein loss better recapitulates disease states and therapeutic pressure, allowing phenotypes to develop under prolonged stress. The AID system is advantageous over CRISPR or siRNA for chronic protein depletion because it removes proteins directly and reversibly, with precise control over the degree of knockdown and recovery, without permanently altering the genome. Additionally, the AID system ensures persistent protein loss, unlike siRNA, which degrades mRNA and inefficiently knocks down long-lived proteins, leading to greater variability in knockdown efficiency. We hypothesized that, upon LT auxin treatment, leukemia cells may evolve mechanisms to overcome protein degradation, particularly for survival-relevant genes, leading to drug resistance, as observed by Petrik *et al.* in *S. cerevisiae* (40). To our knowledge, no investigation of AID resistance has been conducted in mammalian cells, hindering our understanding of the safety and appropriate use of future targeted protein degradation applications.

Our AID model was primarily built on the endogenous *CTCF* gene locus, a multifunctional master transcription factor that organizes chromatin architecture and plays diverse roles in transcriptional regulation (29). Homozygous knockout in mice is lethal, and complete knockout in most cells leads to impaired cell viability. Over the years, several research groups, including ours, have demonstrated the indispensable role of CTCF in maintaining genome-wide chromatin architecture, controlling chromatin accessibility and transcription modulation (23, 30, 41, 42, 43, 44). It is well recognized that cells are addicted to CTCF expression to maintain normal physiology (31, 41, 45, 46, 47, 48). When targeting essential genes or pathways, cells hijack compensatory pathways to counter the consequences of the knockout. For instance, in high-risk MLL-rearranged leukemia cells, HOXA9-targeted cells may switch to dependency on HOXA7 and HOXA10 (49). MYC inhibition can trigger MYCN reactivation in many cancer cell types (50, 51). In our LT degradation resistance study, all mechanisms of auxin resistance occurred in essential components of the AID system. This suggests that these cells may lack compensatory mechanisms upon CTCF loss, as CTCF plays an essential and unique role in maintaining chromatin architecture. However, in the ^HA-miniAID^RBM5 and MBNL1^HA-miniAID^ systems, the other RBM and MBNL family members may compensate for the functional loss upon degradation. Therefore, the selective pressure is required to trigger auxin resistance to degradation. We will continue to study this hypothesis in more AID knock-in models.

Targeted protein degradation has emerged as a promising therapeutic approach to overcome the limitations of traditional therapies, such as small-molecule inhibitors and chemotherapeutic agents (52). With the advent of this technology, proteins that had previously been difficult to target became accessible for study and could be evaluated for their therapeutic potential. The PROTAC system, which uses a ligand to tether the E3 ligase to a protein of interest, has rapidly advanced into clinical trials (13). However, the ligand binds the protein of interest *via* small-molecule targeting, which is not available for many difficult-to-target proteins, such as transcription factors. Additionally, resistance to PROTAC degradation has been observed with mutations arising in essential components of the E3 ligase machinery (26, 27, 28). Unlike the PROTAC system, the AID system utilizes the *Oryza sativa* F-box TIR1 protein, OsTIR1, instead of a linker molecule to bring the AID-tagged protein and E3 ligase machinery together. In the PROTAC system, genetic alterations to essential components of the UPS complex emerged that afforded resistance to degradation. Genetic mutations in the substrate that would abrogate ligand recognition were not observed, unlike what had previously been observed with small-molecule inhibitor resistance (53). In contrast, auxin resistance occurred through genetic alterations of *CTCF*, *miniAID*, and *OsTIR1*^*(F74G)*^ and reduced expression of *OsTIR1*^*(F74G)*^ in the AID system. Without sufficient selection pressure to enforce *OsTIR1*^*(F74G)*^ expression, the human CTCF^AID2^ Clone 27 cells escaped targeted degradation. In addition, two separate genetic mutations, a missense mutation within the AID tag and an early stop codon in CTCF to remove the degron tag, were observed in CTCF^AID2^ clones 3.2 and 17.2, respectively, that mitigated the function of the miniAID tag. The P23S missense mutation in the AID tag occurred within the highly conserved hydrophobic GWPPV motif of the degron tag, which lies on top of auxin within the TIR1 auxin-binding pocket (54). Previously, mutations within this region, including a P-to-S mutation equivalent to P23S, reduced auxin response (55). The tryptophan and proline that flank proline 23 interact with the hydrophobic wall of the TIR1 auxin-binding pocket. P23 stabilizes this interaction and interacts hydrophobically with TIR1 (54). Since the P23S mutation occurred within this highly conserved and structurally relevant motif, the AID tag-OsTIR1^(F74G)^ interaction has likely been inhibited due to the change from a larger, more rigid hydrophobic amino acid to a smaller, more flexible hydrophilic one. Clones 3.2 and 17.2 maintained similar RNA expression levels of the endogenous UPS complex members, such as *SKP1*, *CUL1*, and *RBX1* (Table S1), and retained functional OsTIR1^(F74G)^ under continuous Zeocin selection pressure, supporting that the main mechanism of resistance came from the AID tag genetic alteration and loss.

In the mouse Ctcf^miniAID/miniAID^ BCR-ABL B-ALL cells, mutations were observed in the OsTIR1^(F74G)^ adaptor protein in both cell culture and *in vivo* settings. A W91R missense mutation was observed in OsTIR1^(F74G)^ from *in vivo* auxin-resistant cells. The OsTIR1 protein contains a series of leucine-rich repeat loops that are essential in forming the auxin-binding pocket. A critical feature of these loops is their predominantly hydrophobic charge. The W91R mutation changed a large, hydrophobic amino acid, tryptophan, to a smaller, hydrophilic amino acid, arginine, potentially altering the structure of OsTIR1’s auxin binding pocket.

Taken together, in the AID system, the cells preferred genetic alterations that inhibited the function of the degron tag or *OsTIR1*^*(F74G)*^ over the endogenous UPS complex members. Our study emphasizes the urgent need to investigate resistance-prevention measures before their general application. For instance, the P23S mutation in the AID cassette, which occurs within the highly conserved hydrophobic GWPPV motif of the degron, highlights that this region of the tag is functionally vulnerable to mutation. The AID tag could be engineered with alternative amino acids to examine whether an altered composition can prevent the loss of function of this motif while retaining its degradation sensitivity.

We systematically characterized the mechanisms of degron resistance in mammalian cells, identifying potential limitations of the AID system. However, the study primarily used a single B-ALL cell line, SEM, and mouse primary cells, which limits the generalizability of the resistance mechanisms identified. Additionally, the study focused on the resistance mechanisms underlying auxin-induced degradation of CTCF. A broader panel of genes and cell contexts is necessary to fully establish the relationship between survival-related essential gene dependency and degron resistance. Understanding the challenges of using the AID system for chronic protein loss has broad applicability for future studies of essential genes, allowing researchers to more thoughtfully design LT degradation experiments. In the future, experimental design can be optimized to preclude resistance onset, making the AID system a more powerful and generalizable tool for functional genomic studies.

## Experimental procedures

### Animal model

CD1 nude mice used in this study were 8-week-old females purchased from Jackson Laboratory and maintained in the mouse facility at the St Jude Children’s Research Hospital. The knock-in mouse strain (mCtcf^miniAID^) was generated by the genetically engineered mouse modeling core facility at the St Jude Children’s Research Hospital. All mouse procedure protocols including mouse breeding, leukemia cell injection through tail vein, tissue collection (bone marrow, peripheral blood, and spleen), live animal imaging, auxin treatment through IP injection, euthanasia, and humane endpoint determination utilized in this study were approved by the Institutional Animal Care and Use Committee of St Jude Children’s Research Hospital (protocol #: 3082, 2023/9/14-2026/9/14, PI: Dr Chunliang Li). All mice were maintained in sterile conditions at 20 to 23 °C and 40 to 60% humidity. For the miniAID knock-in mouse generation, a 600-bp single-stranded oligodeoxynucleotide carrying 200-bp 5′ and 3′ homology arms and a 204-bp miniAID sequence was synthesized to insert miniAID to the C terminus of endogenous Ctcf. The single-stranded oligodeoxynucleotide (Genewiz, Inc.) was mixed with Cas9 protein (Protein Production Core Facility at St Jude) and sgCtcf (Genewiz, Inc.). The mixture was then injected into zygotes to obtain Ctcf^miniAID^ founder mice. Founder mice were bred into the C57BL/6 background for germline transmission and homozygous offspring. Genotyping primer sequences are listed in Table S1.

### Vector construction

The Lenti-Cas9-Blast vector was purchased from Addgene (Addgene, 83480). The Lenti-Guide-Puro (Addgene, 52963) plasmid was purchased from Addgene and subcloned to contain the IRES-CFP cassette. A pair of oligonucleotides containing a 20-bp sgRNA sequence targeting the candidate region was synthesized (Thermo Fisher Scientific) and cloned into the Lenti-Guide-Puro-IRES-CFP construct between two BsmBI sites. The retroviral expression system encoding BCR-ABL (P185) in mouse stem cell virus-internal ribosome entry site-GFP vector was provided by Dr Charles J. Sherr (St Jude Children’s Research Hospital, Memphis). The pCL20-SF2-Luc2a-YFP plasmid was provided by Dr Martine F. Roussel (St Jude Children’s Research Hospital, Memphis) and subcloned with the MND promoter after PpuMI and EcoRI digestion to remove the CMV promoter. The pCDH-MND-OsTIR1^(F74G)^-P2A-EGFP-AID2^-^EF1α-RFP (Addgene, 232800) and TRE3G-CTCF-WT-HA-MND-Zeocin^R^ (Addgene, 232801) constructs were generated in a previous study (23). The pCDH-MND-OsTIR1(F74G)-P2A-Zeocin^R^-EF1α-RFP construct was made by using a two-insert infusion cloning to add OsTIR1(F74G)-P2A and Zeocin^R^ to pCDH-EF1α-RFP (NEBuilder HiFi DNA assembly). The OsTIR1(F74G)-P2A cassette was amplified from pCDH-MND-OsTIR1(F74G)-P2A-EGFP-AID2-EF1α-RFP by using “Frag1 OsTIR1 F and R primers”, and the Zeocin expression cassette was amplified from TRE3G-CTCF-WT-HA-MND-Zeocin^R^ by using “Frag2 Zeo F and R primers”. The CMV promoter was removed by SnaB1/Xba1 restriction digestion and replaced by infusion cloning of the MND promoter amplified from TRE3G-CTCF-WT-HA-MND-Zeocin^R^ using the “pCDH MND inf F and R primers”. The pCDH-MND-OsTIR1(F74G)-P2A-Zeocin^R^ cassette was made by excising EF1α-RFP from pCDH-MND-OsTIR1(F74G)-P2A-Zeocin^R^-EF1α-RFP by restriction digestion and blunt-end cloning to circularize the plasmid. TRE3G-CTCF-WT-HA-MND-BSD was made by amplifying CTCF-WT-HA from TRE3G-CTCF-WT-HA-MND-Zeocin^R^ (Addgene, 232801) and cloning by infusion into TRE3G-mCherry-MND-BSD cut with BsiWI and EcoRI. TRE3G-CTCF-Q666∗-HA-MND-BSD was made by amplifying the truncated CTCF and HA-NLS from the TRE3G-CTCF-WT-HA-MND-Zeocin^R^ plasmid and cloning by infusion into TRE3G-mCherry-MND-BSD cut with BsiWI and EcoRI. Primers for cloning were designed with Snapgene software. PCR was performed with CloneAmp polymerase (Clontech) according to the manufacturer’s protocol, and infusion cloning was performed according to the NEBuilder HiFi DNA Assembly protocol. Site-directed mutagenesis was performed to mutate the AID tag of pCDH-MND-OSTIR1^(F74G)^-P2A-EGFP-AID2-EF1α-RFP to generate pCDH-MND-OSTIR1^(F74G)^-P2A-EGFP-P23S-AID2-EF1α-RFP. AID P23S SDM F and R primers were used to amplify pCDH-MND-OSTIR1^(F74G)^-P2A-EGFP-AID2-EF1α-RFP. CloneAmp polymerase was used for the PCR, and the cycling conditions were: 98 °C for 20 s one cycle; 98 °C for 20 s, 55 °C for 20 s, 72 °C for 1 min for 18 cycles; 72 °C for 5 min for one cycle. *Dpn1* was added to the PCR reaction to degrade the methylated DNA template. One microliter of the reaction was transformed into One Shot Stbl3 cells (Thermo Fisher Scientific). The P23S-AID clones were confirmed by Sanger sequencing. All primer and plasmid sequences are listed in Table S1.

### Generation of the AID2 knock-in cell lines

The CTCF^AID2^ cell line Clone 27 was created by infecting the pCDH-MND-OsTIR1^(F74G)^-P2A-EGFP^AID2^-EF1α-RFP construct into a previously derived SEM B-ALL cell line expressing the endogenous CTCF^miniAID-mClover3^ fusion protein and doxycycline-inducible wild-type OsTIR1^WT(23)^. The Clone 27 +HA-CTCF^WT^ was made by transducing Clone 27 with TRE3G-CTCF-WT-HA-MND- Zeocin^R^. The CTCF^AID2^ Clones 3.2, 5, 17.2, 20, and 26 were made by using a sgRNA designed to target the 3′ end of *CTCF* (Synthego), purified Cas9 protein (Protein Production Core Facility at St Jude), and the previously described CTCF-miniAID-mClover3 donor knock-in vector to deliver a miniAID-mClover3 tag to the 3′ end of endogenous *CTCF*. The Lonza Nucleofector 4D SF kit and program EH100 were used to deliver 100 μM sgCTCF, 20 μM Cas9 protein, and 100 ng of the CTCF-miniAID-mClover3 donor knock-in vector to 50,000 SEM wildtype cells. Cells were allowed to recover before sorting for mClover3 expression. A bulk population of mClover3+ cells was transduced with pCDH-MND-OsTIR1^(F74G)^-P2A-Zeocin^R^-EF1α-RFP. After recovery, cells were sorted for double-positive mClover3 and RFP populations. This population was again sorted for single cells, and clones were screened by PCR and immunoblotting to confirm the biallelic miniAID-mClover3 knock-in. A similar knock-in strategy delivered the miniAID tag to the N terminus of *RBM5* and the C terminus of *MBNL1* in SEM cells. Knock-in cassettes were made by Twist. Guide RNA sequences are available in Table S1.

### Mouse bone marrow cell isolation and B-ALL cell culture

Mouse bone marrow cells were isolated from the long bones of 8-week-old Ctcf^miniAID/miniAID^ knock-in mice in a C57BL/6 background. Red blood cells were removed with red blood cell lysis buffer (Sigma, R7757). Cells were suspended and cultured in complete BCM5 with 20 ng/ml IL-7 (R&D Systems, 407-ML-005/CF) for 6 to 8 days to maintain pre-B cell expansion. Then, the cell medium was switched to the Liquid B Cell Medium. About 100,000 cells were infected with MSCV-BCR-ABL(P185)-IRES-GFP, Lenti-Cas9-Blast, and Lenti-sgArf-mCherry. A detailed recipe for the culture medium is provided in Table S1. For LT treatment, mouse BCR-ABL Ctcf^miniAID/miniAID^ cells were first transduced with pCDH-MND-OsTIR1^(F74G)^-P2A-Zeocin^R^. Cells were cultured in a 6-well plate with 1.2 million cells for three replicates in complete liquid B cell medium with Zeocin (50 μg/ml) and with or without 1 μM 5-Ph-IAA treatment. Medium with fresh auxin was changed every 2 to 3 days. Cell numbers were counted every 2 to 3 days using a Countess II automated cell counter (Thermo Fisher Scientific) until the counts reached over 20 million (days 6–7 for non-5-Ph-IAA treatment and day 20 for 5-Ph-IAA treatment groups).

### Cell line culture

The SEM B-ALL cells were cultured in RPMI-1640 medium (Lonza) containing 10% fetal bovine serum (Hyclone), 2 mM glutamine (Sigma), and 1% penicillin/streptomycin (Thermo Fisher Scientific) in a 37 °C incubator with a 5% CO_2_ atmosphere and 95% humidity. Cells were routinely tested for *mycoplasma* contamination (Lookout *Mycoplasma* PCR Detection Kit, Sigma), and SEM cell identity was confirmed by short tandem repeat analysis. 293T cells were cultured in Dulbecco's modified Eagle's medium (Gibco) containing 10% fetal bovine serum (Hyclone), 2 mM glutamine (Sigma), and 1% penicillin/streptomycin (Thermo Fisher Scientific) in a 37 °C incubator with a 5% CO_2_ atmosphere and 95% humidity. For LT auxin treatment, Clone 27 was treated with 1 μM 5-Ph-IAA (MedChemExpress) for 21 days, with media refreshed every 72 to 96 h. Clone 27 + HA-CTCF^WT^ was treated with 1 μM 5-Ph-IAA and 1 μg/ml doxycycline for 21 days, with media refreshed every 72 to 96 h. Clones 3.2, 5, 17.2, 20, and 26 were initially plated at the same density, and cell counts were recorded. These clones were treated with 1 μM 5-Ph-IAA and 100 μg/ml Zeocin (Thermo Fisher Scientific) for up to 36 days, with media refreshed every 72 to 96 h. ^HA-miniAID^RBM5 and MBNL1^HA-miniAID^ clones were treated with 1 μM 5-Ph-IAA and 100 μg/ml Zeocin for up to 23 days, with media refreshed every 72 to 96 h.

### In vivo cell injection and treatment

Ctcf^miniAID/miniAID^ BCR-ABL B-ALL cells were infected with Lenti-MND-luciferase-P2A-YFP to select for a triple-positive population (GFP+, mCherry+, and YFP+). Approximately 100,000 cells were resuspended in 0.15 ml of PBS and then implanted through tail vein injection into 10- to 12-week-old CD1 nude female mice (Jackson Laboratory, 005557). PBS or 5-Ph-IAA (MedChemExpress, 5 mg/kg body weight) was administered daily from the second day after injection. Daily observation and weekly bioluminescence imaging were applied to monitor leukemia progression. Mice were euthanized when moribund: (dehydration, ruffled fur, poor mobility, respiratory distress, and > 20% body weight loss) or showed > 80% leukemia cell burden in peripheral blood. At the humane endpoint, peripheral blood, bone marrow cells, and spleen were collected from mice for immunoblotting.

### MTT assay

In brief, live cells were cultured in 96-well plates with corresponding medium (BCR-ABL cells: 24,000 cells/well; SEM cells: 24,000 cells/well; K562: 10,000 cells/well), with a diluted concentration spectrum of Dasatinib (Selleckchem, S1021) from 10 μM to 0.001 μM for 3 days. MTT (Sigma, M5655-1G) was added to each well at 5 mg/ml. Cells were incubated at 37 °C for 4 h, followed by adding 100 μl of isopropanol/acetic acid (1 ml HCl in 250 ml isopropanol) to stop the reaction. The absorbance was measured using a microplate reader (BioTek Synergy2) at 570 nm. The dose–response curves and IC50 values were calculated using Prism 10 software.

### Flow cytometry

Suspension-cultured cells were collected and filtered through a 70-micron cell strainer before flow cytometry sorting for RFP, mCherry, and/or mClover3-positive cells. The same FL1/FITC channel as GFP was used to detect fluorescence from mClover3. Dead cells were excluded by DAPI. For the staining of BCR-ABL B-ALL cells, cells were mixed with 100 μl APC-B220 and PE-IgM antibody buffer (0.1% BSA PBS+ 1:50 antibody), followed by incubation in the dark room at room temperature for 20 min. Cells were washed twice with 4 ml of 0.1% BSA PBS and resuspended in 200 μl of 0.1% BSA PBS for flow analysis. Data analysis and presentation were produced by FlowJo software.

### Competitive proliferation assay

BCR-ABL B-ALL pre-B cells were transduced with Lenti-Cas9-Blast, selected by blasticidin and then transduced with individual sgRNAs to *Myc* and *Ctcf* in CFP-expressing lentiviral vectors. The day after transduction, the cells were washed three times with PBS to remove the virus. On the second day after transduction, the percentage of CFP-positive cells was measured by flow cytometry and used as the reference time point. CFP fluorescence was measured again on days 5 and 8 post-transduction. The percentage of CFP-positive cells was normalized to the starting time point.

### Immunoblotting

Cells were lysed in RIPA buffer, and lysates were run on an SDS-PAGE gel (Thermo Fisher Scientific), followed by protein transfer to a PVDF membrane (Bio-Rad) at 100 V for 1 h. Membranes were blocked with 5% nonfat milk in TBS-T (10 mM Tris, pH 8.0, 150 mM NaCl, 0.5% Tween-20) for 1 h at room temperature, then incubated overnight with primary antibodies at 4 °C with gentle rocking (GAPDH, ThermoFisher Scientific, AM4300; CTCF, Diagenode, C15410210-50; CTCF, Millipore, 07729; miniAID, MBL, M214-3; GFP, Santa Cruz, sc-9996; HSC70, Santa Cruz, sc-7298; HA, Cell Signaling, 3724S). Membranes were washed in TBS-T and then incubated with secondary antibodies in 5% nonfat milk/TBS-T for 1 h at room temperature [donkey anti-rabbit IgG HRP (GE Healthcare) for CTCF and HA primary blots; sheep anti-mouse IgG HRP (GE Healthcare) for GAPDH, miniAID, GFP, and HSC70 primary blots]. After washing in TBS-T, blots were developed with ECL (PerkinElmer) and visualized by using the Imaging System (Licor).

### RNA-seq

The Zymo RNA Clean and Concentrator Kit with in-column DNase treatment (Zymo Research) was used to isolate RNA from cells. The Kapa RNA HyperPrep Kit with RiboErase (HMR) was used to prepare cDNA libraries.

### Quantitative real-time Q-PCR

The high-capacity cDNA reverse transcriptase kit (Applied Biosystems) was used to make cDNA, and the FAST SYBR Green Master Mix was used for real-time RT-qPCR (Applied Biosystems). The ^ΔΔ^CT method was used to determine relative expression levels (56). RT-qPCR primers for GAPDH and OsTIR1^(F74G)^ are listed in Table S1.

### In vivo bioluminescence imaging

Imaging was performed using IVIS Spectrum or IVIS-200 systems. Five to 10 min prior to imaging, animals were injected intraperitoneally with D-Luciferin (15 mg/ml in sterile saline) at a dose of 150 mg/kg (10 μl/g of body weight). After administration, animals were anesthetized using Isoflurane and maintained *via* nosecone on a heated imaging bed within the system for the duration of the scan. After imaging, animals were allowed to recover on a heating blanket under observation and supplemented with oxygen as required.

### Amplicon-based deep sequencing and Sanger sequencing

RNA was extracted from Clone 17.2 cells at various time points during the LT 5-Ph-IAA/Zeocin treatment, and cDNA was synthesized. First-round PCR primers were designed to amplify a ∼340-bp region containing the C > T mutation in exon 11 and to add the Nextera Read1 and Read2 adapter sequences. PCR products were gel-extracted and amplified in a second round of PCR using indexing primers (Illumina i5(S)/i7(N)). Purified indexed PCR fragments were submitted for amplicon sequencing, 150-bp single-end reads on the Illumina NovaSeq 6000. The C > T mutation of the miniAID in Clone 3.2 was amplified and sequenced by Sanger sequencing. Primer sequences are available in Table S1.

### Immunoprecipitation and mass spectrometry

For IP-MS, 100 million cells of each of the following conditions were collected: Clone 3.2 parental, Clone 3.2 parental + 1 μM 5-Ph-IAA for 24 h, Clone 3.2 resistant, and Clone 17.2 resistant + 1 μM 5-Ph-IAA for 24 h. Cells were washed once in cold PBS and then resuspended in 10 ml cold nondenaturing IP buffer [200 mM Tris pH 7.4, 137 mM NaCl, 2 mM EDTA, 1% NP-40, protease inhibitors (PIs)] and rotated at 4 °C for 30 min. Cells were briefly sonicated, and cell lysis was monitored by trypan blue staining. Cell debris was removed from the lysates by centrifugation at 4000 rpm for 10 min. Lysates were transferred to a new 15 ml conical tube. CTCF antibody (Diagenode) was added at a concentration of 5 μg/ml and incubated with lysates overnight on a rotator at 4 °C. The following day, 250 μl prewashed Protein G Dynabeads (Pierce) were added to the lysates and incubated for 6 h on a rotator at 4 °C. Tubes were placed on a magnetic stand, and lysates were removed. Beads were washed twice with IP buffer, then 5 times with PBS. At the last wash, the beads were transferred to a new tube. Beads were shipped on dry ice to the Mass Spectrometry Technology Access Center at the McDonnell Genome Institute (MTAC@MGI) at Washington University School of Medicine for further analysis.

### ChIP-seq

CTCF^AID2^ clones 5, 3.2, and 17.2 were transduced with either TRE3G-CTCF-WT-HA-BSD (CTCF^AID2-WT-HA^ clones) or TRE3G-CTCF-Q666∗-HA-BSD (CTCF^AID2-Q666^∗^-HA^ clones) to make three biological replicates for each CTCF variant. All clones were treated with 1 μM 5-Ph-IAA for 24 h. Six hours after treatment initiation, 0.05 μg/ml or 1.0 μg/ml doxycycline was added to CTCF^AID2-WT-HA^ clones and CTCF^AID2-Q666^∗^-HA^ clones, respectively. Twenty million cells of each were collected and fixed with 1% formaldehyde for 8 min at room temperature in Covaris fixation buffer (Covaris TruChIP Chromatin Shearing Kit, 520154). Chromatin was prepared according to the TruChIP Chromatin Shearing Kit protocol. Chromatin was sheared on the Covaris M220 ultrasonicator, set to a duty factor of 10 and 200 cycles/burst, for 10 min at a set point of 6 °C. Sheared chromatin was centrifuged for 10 min at 8000×*g*. Clarified chromatin was amended to a final concentration of 50 mM Tris-HCl, pH 7.4, 100 mM NaCl, 1 mM EDTA, 1% NP-40, 0.1% SDS, and 0.5% Na deoxycholate plus PI. Active motif spike-in chromatin (53083) and antibody (61686) were added to sheared chromatin according to the manufacturer’s protocol. About 10 μg of CTCF antibody (Diagenode, c15410210-50) was also added, and the chromatin was rotated at 4 °C overnight. The following day, Protein G Dynabeads (Invitrogen, 10004D) were added, and the samples were rotated at 4 °C for 4 h. Samples were placed on a magnet, and the unbound fraction was removed. Beads were washed two times in high-salt wash buffer 1 (50 mM Tris–HCL pH 7.4, 1 M NaCl, 1 mM EDTA, 1% NP-40, 0.1% SDS, 0.5% Na deoxycholate plus PI) and one time with wash buffer 2 (20 mM Tris–HCL pH 7.4, 10 mM MgCl_2_, 0.2% Tween-20 plus PI). For the final wash, the beads were resuspended in wash buffer 2 and transferred to a new 1.5-mL Eppendorf tube. DNA was eluted and de-crosslinked in 1X TE plus 1% SDS, proteinase K, and 400 mM NaCl at 65 °C for 4 h. Phenol, chloroform, and isopropyl alcohol were used to precipitate the DNA. Libraries were constructed using the NEBNext Ultra II NEB Library Prep Kit and NEBNext Multiplex oligos for Illumina.

### RNA-seq data analysis

We performed the paired-end 101-cycle sequencing on the NovaSeq 6000 sequencer (Illumina) and analyzed the data using a standard pipeline. Briefly, raw reads were trimmed using TrimGalore (v0.6.3, “--paired --retain_unpaired”) and aligned to the *Homo sapiens* reference genome GRCh38.p13 (hg38) by using STAR (v2.7.9a) (57). Gene-level read quantification was performed using RSEM (v1.3.1) on the Gencode annotation v31 (58). Differential gene expression analysis was conducted by using the TMM normalization method (genes with CPM < 1 in all samples were removed), followed by Limma-voom analysis using the “voom”, “lmFit”, and “eBayes” functions from the limma R package (59). Complete gene expression profiling was shown in Table S2.

### Mass spectrometry and data analysis

Trypsin (800 ng) was added to immunoprecipitated proteins on the beads for 6 h at 37 °C. The initial digested samples were centrifuged for 2 min at 5000 × *g*, and the supernatants were collected into fresh tubes. Beads were washed twice with 100 mM ammonium bicarbonate, and the supernatants were pooled. The resulting samples were reduced with 20 mM dithiothreitol at 37 °C for 1 h, and cysteine was alkylated with 80 mM iodoacetamide for 45 min in the dark. Samples were incubated overnight with 600 ng of trypsin at 37 °C. The resulting peptides were desalted using solid-phase extraction on a C18 Spin column and eluted with 0.1% FA in 80% ACN. Peptides were analyzed *via* LC-MS/MS by using a Vanquish Neo UHPLC System coupled to an Orbitrap Eclipse Tribrid Mass Spectrometer with FAIMS Pro Duo interface (Thermo Fisher Scientific). The sample was loaded on a Neo trap-cartridge coupled with an analytical column (75 μm ID x 50 cm PepMa × ™ Neo C18, 2 μm). Samples were separated using a linear gradient of solvent A (0.1% formic acid in water) and solvent B (0.1% formic acid in ACN) over 120 min. For MS acquisition, FAIMS alternated between CVs of −35 V and −65 V with a cycle time of 1.5 s per CV. MS1 spectra were acquired at 120,000 resolution with a scan range from 375 to 1500 m/z, an AGC target set at 300%, and a maximum injection time set to Auto mode. Precursors were filtered using monoisotopic peak determination set to peptide, charge states 2 to 7, and dynamic exclusion of 60 s with ±10 ppm tolerance. For the MS2 analysis, the isolated ions were fragmented by assisted higher-energy collisional dissociation at 30% and acquired in an ion trap. The AGC and maximum IT were standard and dynamic modes, respectively. Data were searched using Mascot (v.3.1, Matrix Science) against a customized database comprising the Swiss-Prot human database and 11 isoforms of the transcriptional repressor CTCF. Trypsin was selected as the enzyme, and the maximum number of missed cleavages was set to 3. The precursor mass tolerance was set to 10 ppm, and the fragment mass tolerance was set to 0.6 Da for the MS2 spectra. The carbamidomethylated cysteine was set as a static modification, and dynamic modifications were set as oxidized methionine, deaminated asparagine/glutamine, and protein N-terminal acetylation. The search results were validated with a 1% FDR protein threshold and a 90% peptide threshold using Scaffold (v5.3.0, Proteome Software).

### ChIP-seq data analysis

Sequencing was performed by single-end 51-cycle sequencing on the NovaSeq 6000 sequencer (Illumina). Raw reads were trimmed using TrimGalore (v0.6.3) and aligned to the *H. sapiens* reference genome GRCh38.p13 (hg38) genome using BWA (v0.7.17-r1198). Duplicated and low mapping quality reads were removed using “bamsormadup” function from the biobambam2 tool (v2.0.87) and samtools (version 1.9, parameter “-q 1 -F 1024”) (60). The fragment size in each sample was estimated based on the cross-correlation profile calculated with SPP (v1.11). Fragments were extended to fragment size and normalized to 15 million reads to generate bigwig files. Macs2 was used to call peaks using parameters “-g hs --nomodel –extsize < SPP_fragmentSize>”. Reads were extended to < SPP_fragmentSize> and count at reproducible peaks, after trim the mean of the M-value normalization, differential peaks were identified using empirical Bayes statistical tests after linear fitting from the voom package (R 4.3.2, edgeR 3.42.4, limma 3.56.2). For downstream analyses, heatmaps and Spearman’s correlation coefficients were generated using deepTool (61).

## Data availability

RNA-seq data generated in this study were deposited at NCBI GEO as GSE307188 (reviewer token: yrwviuquzdkhzsl). The mass spectrometry data generated in this study were deposited at PXD. The mass spectrometry proteomics data have been deposited to the ProteomeXchange Consortium *via* the PRIDE partner repository with the dataset identifier PXD060834 (reviewer token: ipsPHDCv3fdo). Tables S2 and S3 summarize the RNA-expression and ChIP-seq profiling. Code repositories included RNA-seq and ChIP-seq (https://figshare.com/collections/CTCF-AID2/6186670). Raw data related to figures have been deposited in Table S4, and raw immunoblots have been deposited as a “raw data file”.

## Supporting information

This article contains supporting information.

## Conflict of interest

The authors declare that they have no conflicts of interest with the contents of this article.
