# Supplementary material for: Deciphering resistance mechanisms to auxin-inducible protein degradation in mammalian cells
Source: J Biol Chem. 2026 Jun 4;302(7):113232. doi: 10.1016/j.jbc.2026.113232 (PMC13325307; doi:10.1016/j.jbc.2026.113232)

Supplementary Figure Legend

Supplementary Figure S1. Determine auxin-inducible degradation resistance in human CTCF-miniAID knock-in cell lines.

A. Schematic illustration of the Lentiviral OsTIR1$^{(F74G)}$ cassette, pCDH-MND-OsTIR1$^{(F74G)}$-P2A-Zeocin^{R}, and the miniAID-mClover3 tag knock-in design at the *CTCF* locus.

B. Immunoblot of Clone 27 parental and resistant cells with a titration of 5-Ph-IAA treatment for 24 hours. The CTCF immunoblot confirms that resistant cells were insensitive to auxin treatment even at higher doses. GAPDH was included as a loading control.

C. Quantitative PCR showing *OsTIR1*$^{(F74G)}$ expression in Clone 27 resistant cells is lower than that of parental cells. The expression shown is relative to GAPDH. *** p-value ≤ 0.001 calculated by unpaired Student's *t*-test. N=3

D. Graph depicting increasing cell counts for Clones 3.2 and 17.2 after 25 days in culture with continuous 1 μM 5-Ph-IAA/ 100 μg/mL Zeocin treatment. Cell counts taken from days 25 to 34 are shown.

E, F, G. Immunoblots from long-term 5-Ph-IAA culture of Clones 5, 20, and 26. All cultures were kept under continuous 1 μM 5-Ph-IAA/ 100 μg/mL Zeocin treatment. No T=no treatment. Lysates were blotted for CTCF, and GAPDH was included as a loading control. CTCF^{miniAIDmCLover3} expression did not escape auxin treatment in Clones 20 and 26.

Supplementary Figure S2. Time-dependent tracing of miniAID mutation upon long-term auxin treatment.

A. Immunoblots from long-term 5-Ph-IAA culture of Clone 3.2 kept under continuous 1 μM 5-Ph-IAA/ 100 μg/mL Zeocin. Lysates were probed with antibodies against CTCF and miniAID, and GAPDH was used as a loading control. No T=no treatment. The auxin-resistant CTCF appeared

around Day 25 of treatment. Auxin-resistant CTCF, which carried the P23S-AID mutation, was not detectable by immunoblotting with a miniAID antibody.

B and C. Sanger sequencing trace images from PCRs of Clone 3.2 parental and treated cells [cDNA (B) and genomic DNA (C)] of the miniAID sequence showed the C > T mutation in the miniAID tag of Clone 3.2 cells treated with 1 μM 5-Ph-IAA/ 100 μg/mL Zeocin for 34 days.

Supplementary Figure S3. Time-dependent tracing of CTCF mutation upon long-term auxin treatment by immunoblot and mass spec analysis.

A. The full $CTCF^{miniAID3-mClover3}$ sequence representing mass spectrometry peptide coverage of the $CTCF^{miniAID-mClover3}$ protein in Clone 3.2 parental and Clones 3.2 and 17.2 resistant cells. Peptides that match the sequence with high confidence are shaded yellow and green.

B. Sequence illustration pinpointing the trypsin cleavage breakpoint observed by IP-MS in Clone 17.2 resistant cells. A red asterisk denotes the C > T mutation identified by RNA-seq of resistant cells.

C and D. Immunoblot from long-term 5-Ph-IAA culture of Clone 17.2 kept under continuous 1 μM 5-Ph-IAA/ 100 μg/mL Zeocin. Lysates were probed for CTCF and miniAID, and GAPDH was included as a loading control. Two replicates are shown. The auxin-resistant truncated-CTCF appeared around treatment Day 18 of rep 1 and treatment Day 25 of rep 2.

E. Chart representing the ultra-sensitive deep sequencing readout percentage of the C > T mutation's emergence in cDNA throughout continuous treatment with 1 μM 5-Ph-IAA/100 μg/mL Zeocin for up to 34 days in Clone 17.2. Two replicates were included.

Supplementary Figure S4. CTCF ChIP-seq of $CTCF^{AID2}$ clones expressing either $CTCF^{WT-HA}$ or $CTCF^{Q666*-HA}$

A. Immunoblots of CTCF^{AID2} clones C5, C3.2, and C17.2 with doxycycline-inducible exogenous expression of CTCF$^{WT\text{-}HA}$ or CTCF$^{Q666*\text{-}HA}$. Cells were treated for 24 hours with 1μM 5-Ph-IAA to remove endogenous CTCF. After 6 hours of treatment, 0.05 μg/mL or 1.0 μg/mL doxycycline was added to CTCF$^{AID2\text{-}WT\text{-}HA}$ or CTCF$^{AID2\text{-}Q666*\text{-}HA}$ clones, respectively. The HA and CTCF antibodies were used to assess exogenous CTCF protein expression. GAPDH antibody was used as a loading control.

B. Principal component analysis (PCA). PC1 accounted for 39.3% of the variance between the CTCF$^{AID2\text{-}WT\text{-}HA}$ and CTCF$^{AID2\text{-}Q666*\text{-}HA}$ clones. PC2 accounted for only 18.8% of the variance among the replicates.

C-D. Heatmap of CTCF ChIP-seq of CTCF$^{AID2\text{-}WT\text{-}HA}$ or CTCF$^{AID2\text{-}Q666*\text{-}HA}$ clones C3.2, C5, and C17.2 at control regions that showed the least changes in CTCF binding at peaks with and without the CTCF consensus binding motif. Control peaks were randomly selected from those with a cutoff of log2 FC < 1 and FDR > 0.05.

E-F. Heatmap of CTCF ChIP-seq of CTCF$^{AID2\text{-}WT\text{-}HA}$ or CTCF$^{AID2\text{-}Q666*\text{-}HA}$ clones C3.2, C5, and C17.2 showed increased CTCF peak density at 7,727 CTCF binding peaks in CTCF$^{AID2\text{-}WT\text{-}HA}$ vs CTCF$^{AID2\text{-}Q666*\text{-}HA}$ clones (log2 FC > 1, FDR < 0.05). Less than 10% of the increased peaks occurred in regions lacking the consensus CTCF binding motif (log2 FC > 1, FDR < 0.05).

G. CTCF motif analysis of the 7,727 CTCF binding peaks with increased density in CTCF$^{AID2\text{-}WT\text{-}HA}$ vs CTCF$^{AID2\text{-}Q666*\text{-}HA}$ clones showed that the CTCF motifs were most enriched.

H. CTCF ChIP seq tracks of CTCF$^{AID2\text{-}WT\text{-}HA}$ and CTCF$^{AID2\text{-}Q666*\text{-}HA}$ clones (C3.2 and C17.2) at the *BLCAP* locus showed increased CTCF binding in CTCF$^{AID2\text{-}Q666*\text{-}HA}$ clones. No variance in binding was observed at most CTCF binding loci, as represented at the *ISYNA1* locus.

Supplementary Figure S5. No auxin-inducible degradation resistance observed in human miniAID-RBM5 and MBNL1-miniAID knock-in cell lines.

A. Schematic diagram of the HA mini-AID knock-in design to the N-terminus of *RBM5* in SEM cells. 5'-HRA and 3'-HRA designate the homology recombinant arms (HRA) that flank the targeted knock-in site. Immunoblots for RBM5 and HA showing that $^{HA\text{-}miniAID}$RBM5 remained sensitive to auxin throughout continuous 1 μM 5-Ph-IAA/ 100 μg/mL Zeocin treatment.

B. Schematic diagram of the HA mini-AID knock-in design to the C-terminus of *MBNL1* in SEM cells. Immunoblots for MBNL1 and HA showing that MBNL1$^{miniAID\text{-} HA}$ remained sensitive to auxin throughout continuous 1 μM 5-Ph-IAA/ 100 μg/mL Zeocin treatment.

Supplementary Figure S6. Full characterization of Ctcf-miniAID knock-in mice and primary BCR-ABL B-ALL cells.

A. Successful knock-in was confirmed by genotyping PCR in founder mice. The genotyping PCR primers were combined to detect the 5' and 3' junctions of the miniAID. 5' F (red forward arrow) and R (blue arrow) primers, KI= 478bp; 3' F (black arrow) and R (red reverse arrow) primers, KI= 448bp; Homology arm primers outside the inserted miniAID sequences (red arrows): WT= 689bp, and KI= 893bp. A wild-type mouse was used as a negative control.

B. Successful germline transmission to the F1 progeny was confirmed by genotyping PCR. Primers were designed against the flanking regions of the miniAID cassette (red arrows). Tissues from wild-type and founder knock-in mouse 3F were used as negative and positive controls, respectively.

C. Immunoblotting of Ctcf-miniAID expression from tissues, including liver, spleen, and kidney of wild-type, heterozygous, and homozygous knock-in mice. All tissues expressed Ctcf-miniAID fusion protein with a molecular weight 7kDa higher than the wild-type Ctcf protein.

D. Simple schematic diagram representing BCR-ABL translocation and sensitivity to second-generation tyrosine kinase inhibitor. The translocation occurs between the ABL gene on chromosome 9 and the BCR gene on chromosome 22. Two translocation fusion proteins are

observed: p210, a hallmark of chronic myeloid leukemia (CML), and p185, associated with ALL. TKI, tyrosine kinase inhibitor.

E. MTT assay was conducted in parental $Ctcf^{miniAID/miniAID}$ BCR-ABL B-ALL, following a 3-day treatment with Dasatinib in a dosage-dependent manner. K562 was used as a positive control cell line carrying the endogenous BCR-ABL translocation, and SEM was used as a non-BCR-ABL negative control cell line; replication=3.

F. Bright field image demonstrating that $Ctcf^{miniAID/miniAID}$ BCR-ABL B-ALL cells are highly proliferative. Flow cytometry was performed to illustrate the high expression of B220 and the absence of IgM in the homogeneous populations. The eGFP channel showed BCR-ABL expression from MSCV-BCR-ABL-IRES-eGFP.

G. Ctcf and miniAID immunoblot of $Ctcf^{miniAID/miniAID}$ BCR-ABL B-ALL cells showed Ctcf degradation after 6 hours of 5-Ph-IAA treatment that was restored after auxin removal. Hsc70 was included as a loading control.

H. Schematic diagram illustrating CPA assay: $Ctcf^{miniAID/miniAID}$ BCR-ABL B-ALL cells were infected with Cas9 and sgRNA-CFP against the coding exons of *Ctcf*. Flow cytometry analysis of CFP fluorescence was performed on cells at Day 2, Day 5, and Day 8.

I. CPA analysis showed time-dependent decreased cell proliferation after Ctcf knock-out. sgNT was used as a negative control, and sgMyc as a positive control.

Supplementary Figure S7. Establishment of the *in vivo* $Ctcf^{miniAID/miniAID}$ BCR-ABL B-ALL mouse model and characterization of auxin resistance following long-term *in vivo* 5-Ph-IAA treatment.

A. MTT assay showed $Ctcf^{miniAID/miniAID}$ BCR-ABL B-ALL cells after long-term 5-Ph-IAA treatment were still sensitive to Dasatinib, similar to parental cells. SEM cells were included as a negative control.

B. Immunoblotting of Ctcf in Ctcf$^{miniAID/miniAID}$ BCR-ABL B-ALL cells treated long-term (Day 29) with 5-Ph-IAA showed auxin resistance. Hsc70 was included as a loading control.

C. Schematic diagram showing *in vivo* model establishment: 100K cells were injected through the tail vein. Weekly peripheral blood was collected for flow analysis until the endpoint.

D. Flow analysis of weekly leukemia cell percentage (GFP+ cells) from peripheral blood.

E. Image showing spleen size after Ctcf$^{miniAID/miniAID}$ BCR-ABL B-ALL cell injection compared with the negative controls.

F. Spleen weight/ body weight of the Ctcf$^{miniAID/miniAID}$ BCR-ABL B-ALL cell-injected mice compared with negative controls.

G. Spleen H&E staining of the control and Ctcf$^{miniAID/miniAID}$ BCR-ABL B-ALL cell-injected mice.

H. Spleen eGFP staining of the control and Ctcf$^{miniAID/miniAID}$ BCR-ABL B-ALL cell-injected mice.

I. After Ctcf$^{miniAID/miniAID}$ BCR-ABL-B-All cells were infected with luciferase-YFP, immunoblotting of Ctcf, miniAID confirmed Ctcf-miniAID fusion protein degradation after 6-hour 5-Ph-IAA treatment before *in vivo* transplantation.

Supplementary Table Legend

Supplementary Table S1. Culture medium recipe of Pre-B and B-ALL cells.

Supplementary Table S2. Total RNA-seq and expression values of parental and resistant cells.

Supplementary Table S3. Differential ChIP-seq analysis of CTCF binding in WT and Q666* mutant expressing cells.

Supplementary Table S4. Raw data associated with Figures 1C, 4F, Supplementary Figures S1C, S1D, S6E, S6I, S7A, S7D and S7F.

St. Jude - Confidential

Supplementary Figure 1

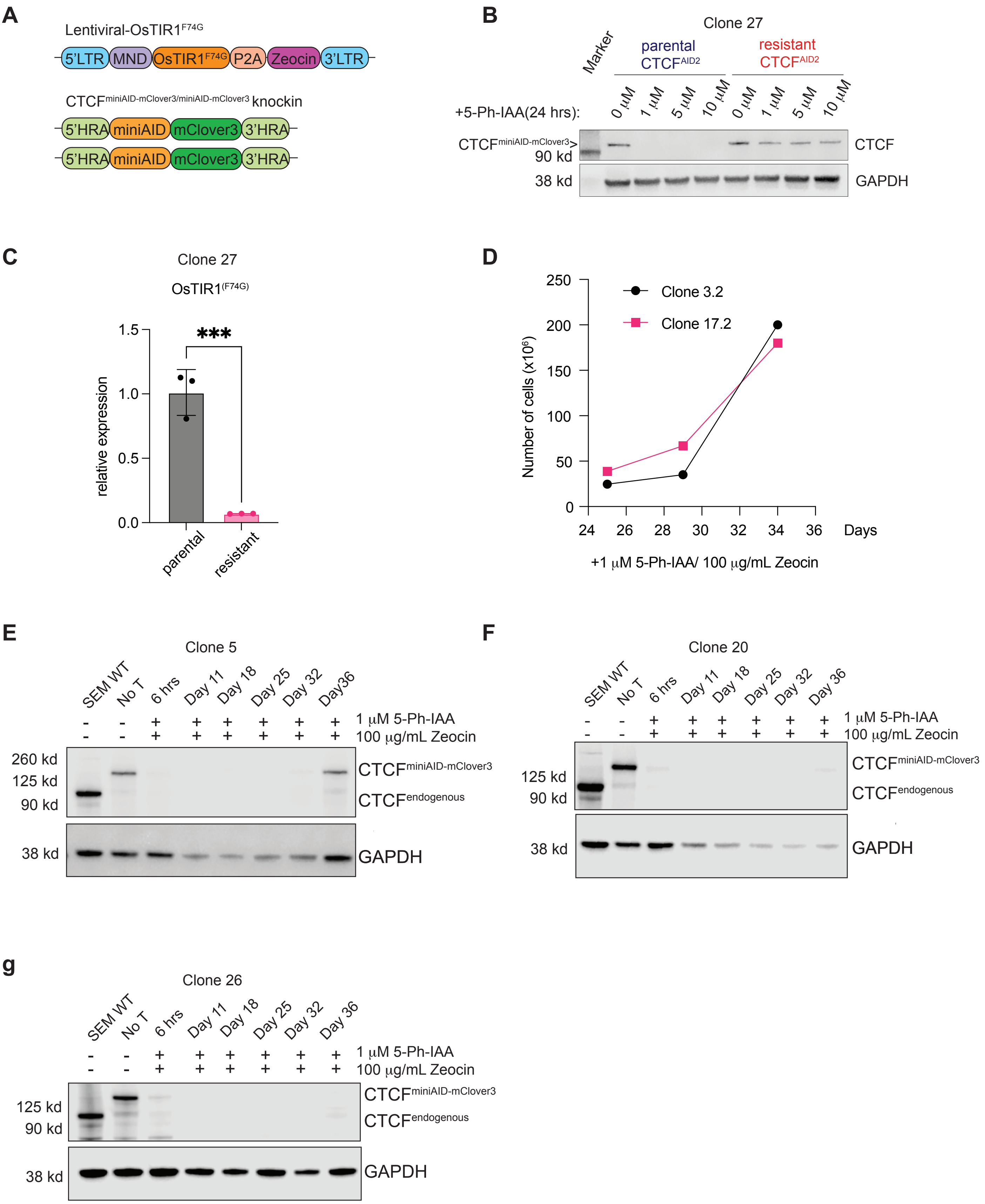

Supplementary Figure 2

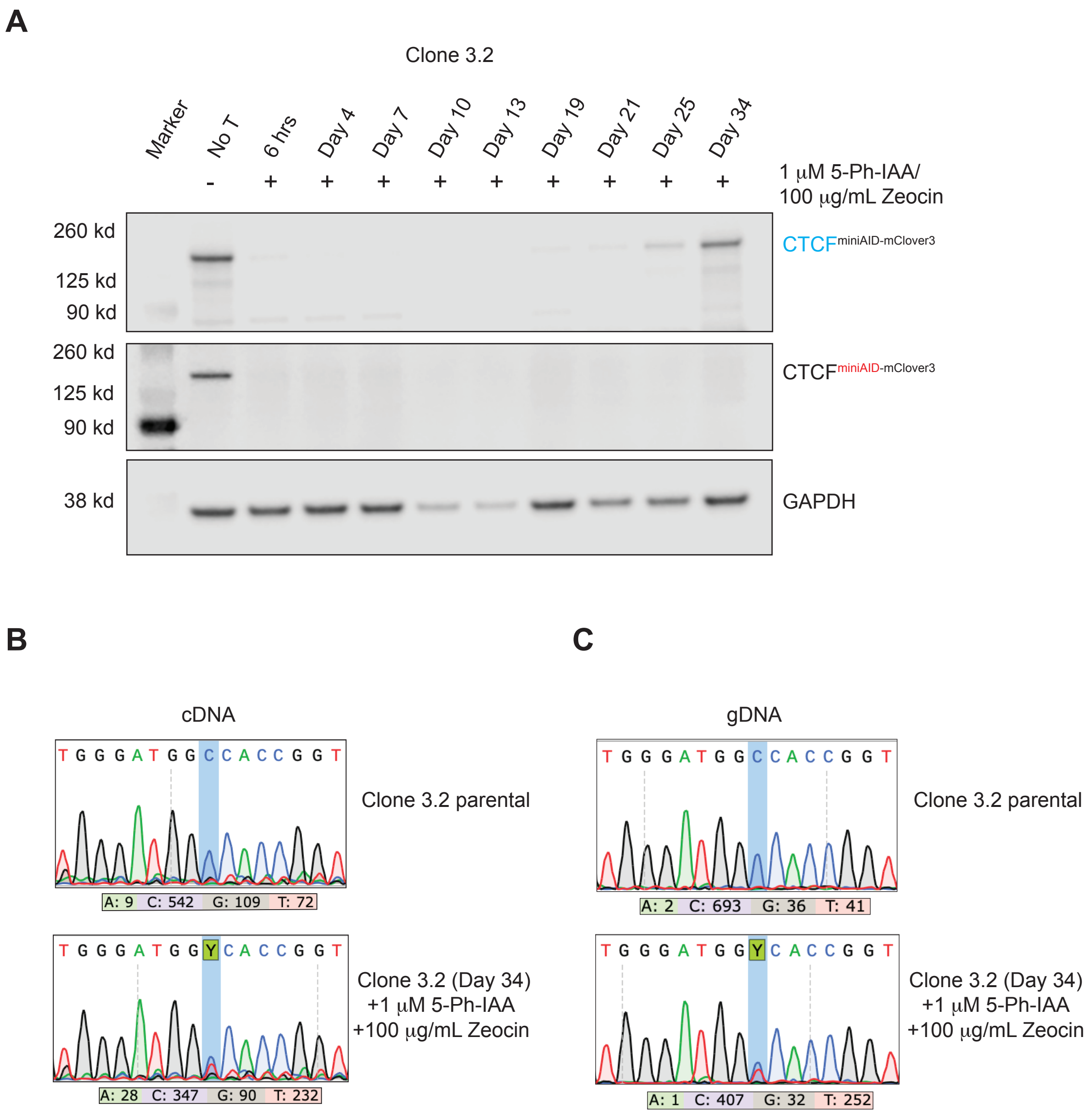

Supplementary Figure 3

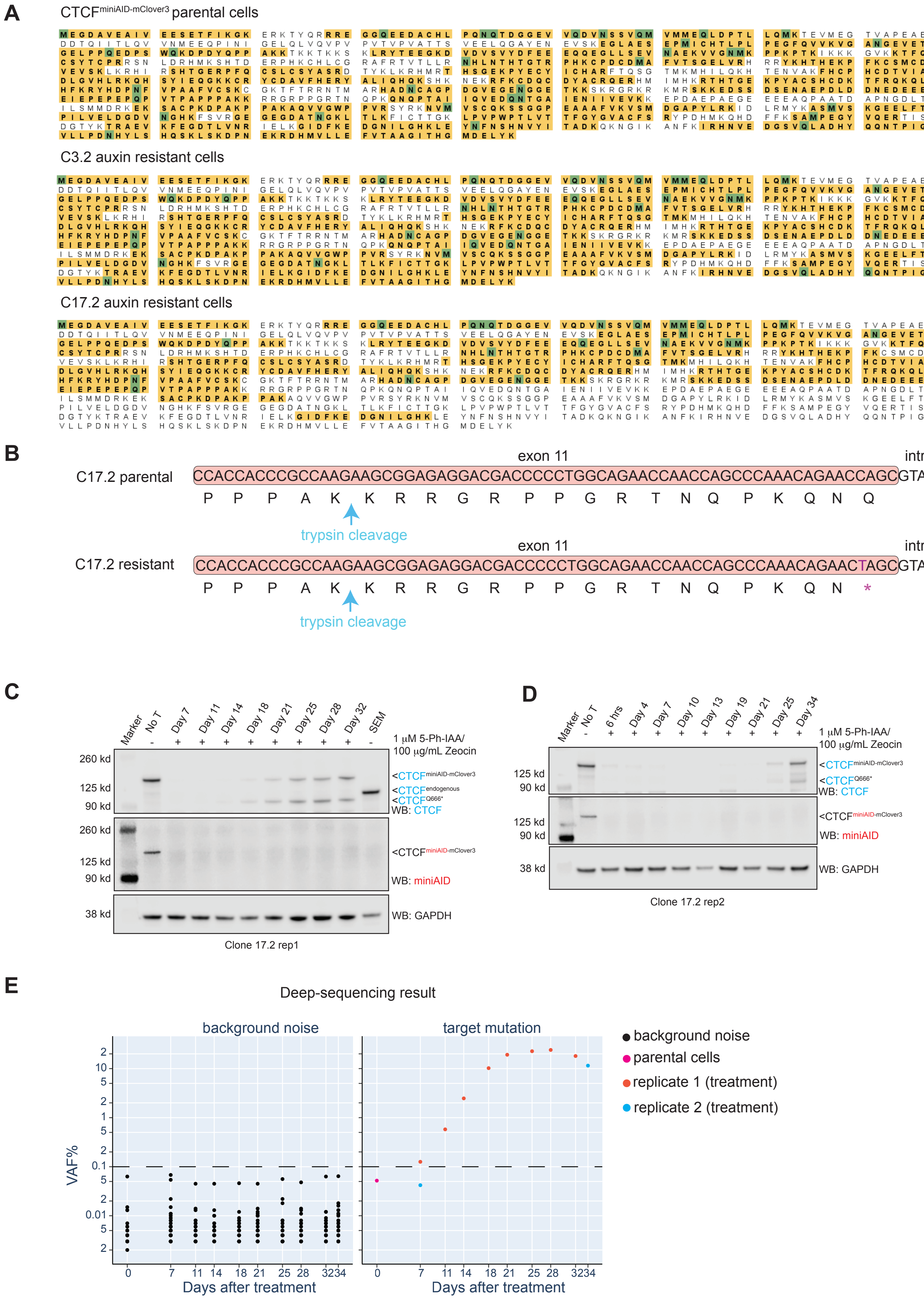

Supplementary Figure 4

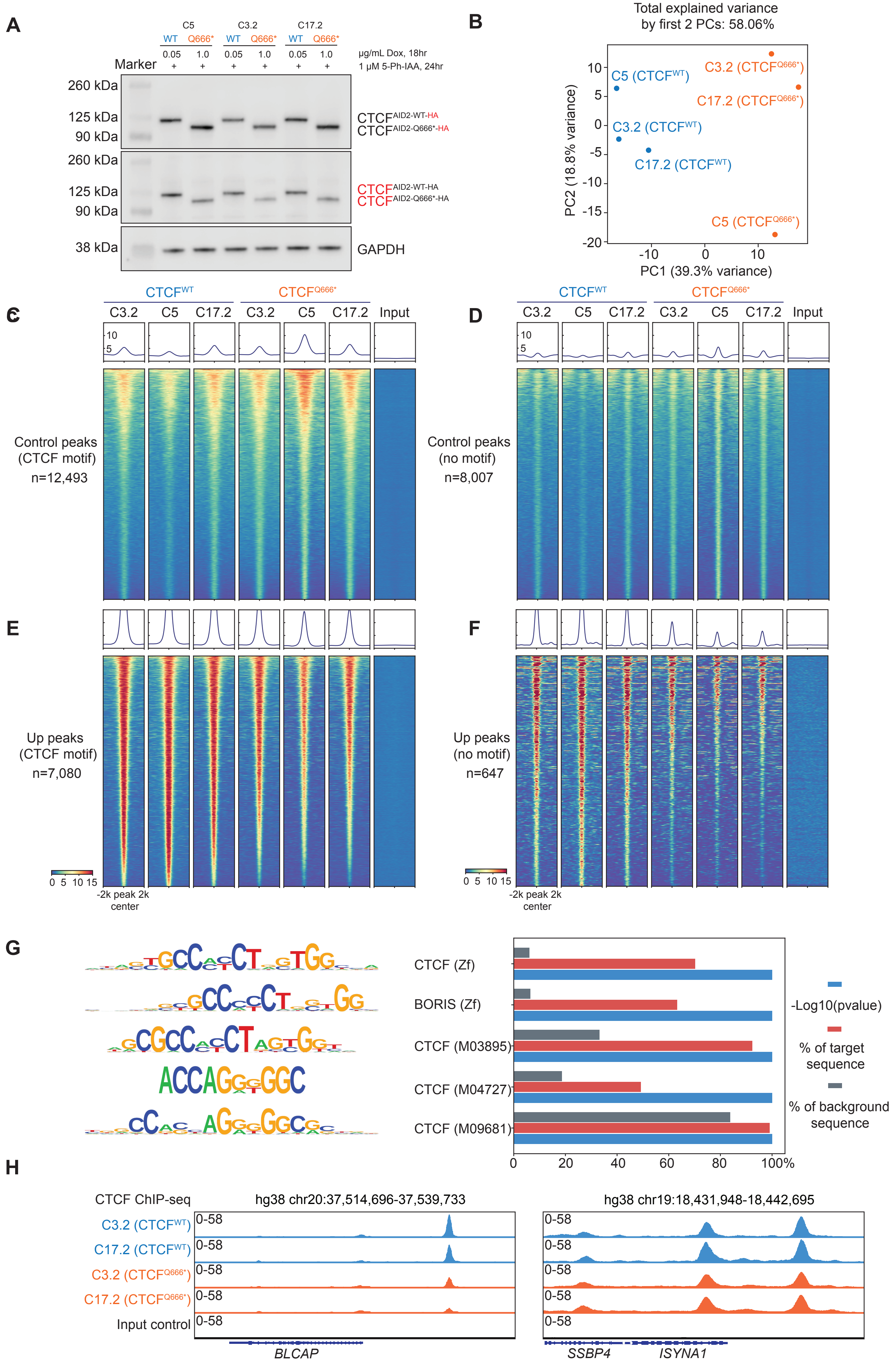

Supplementary Figure 5

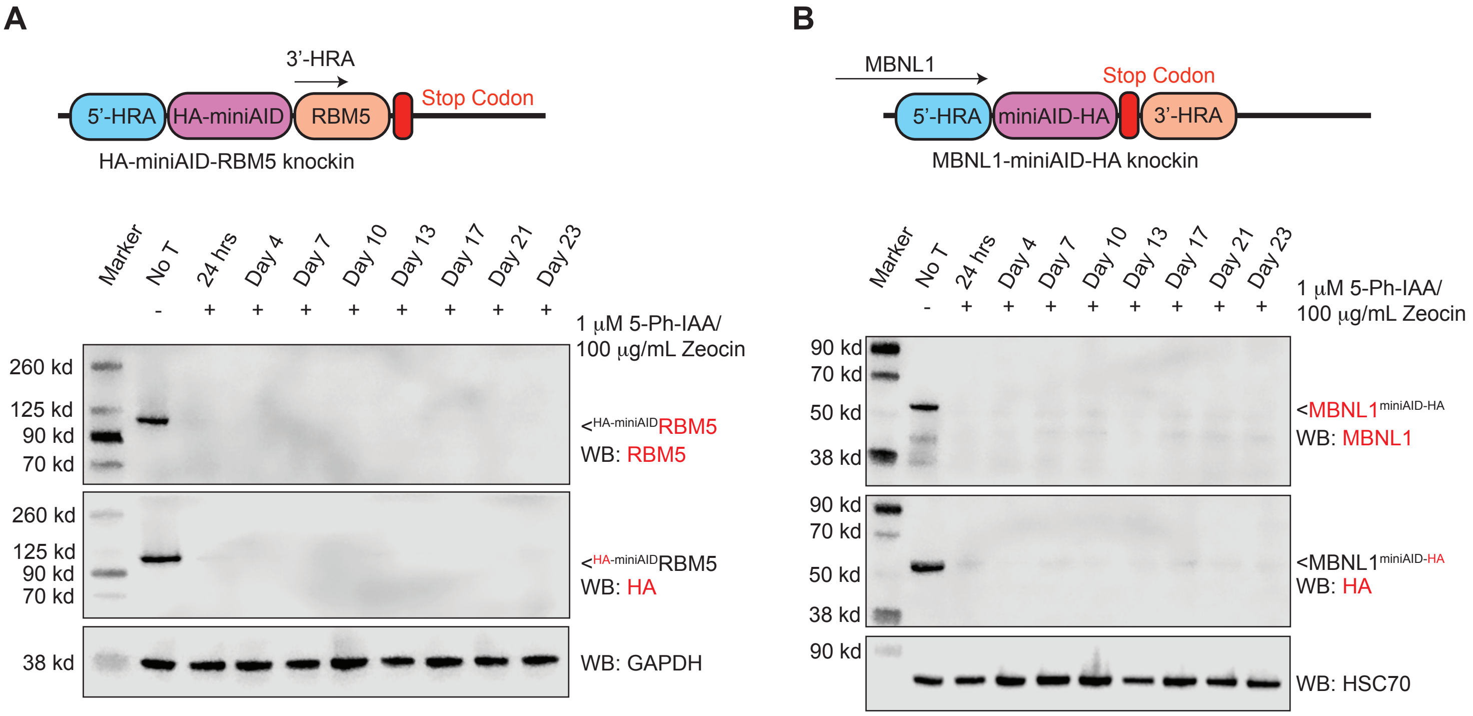

Supplementary Figure 6

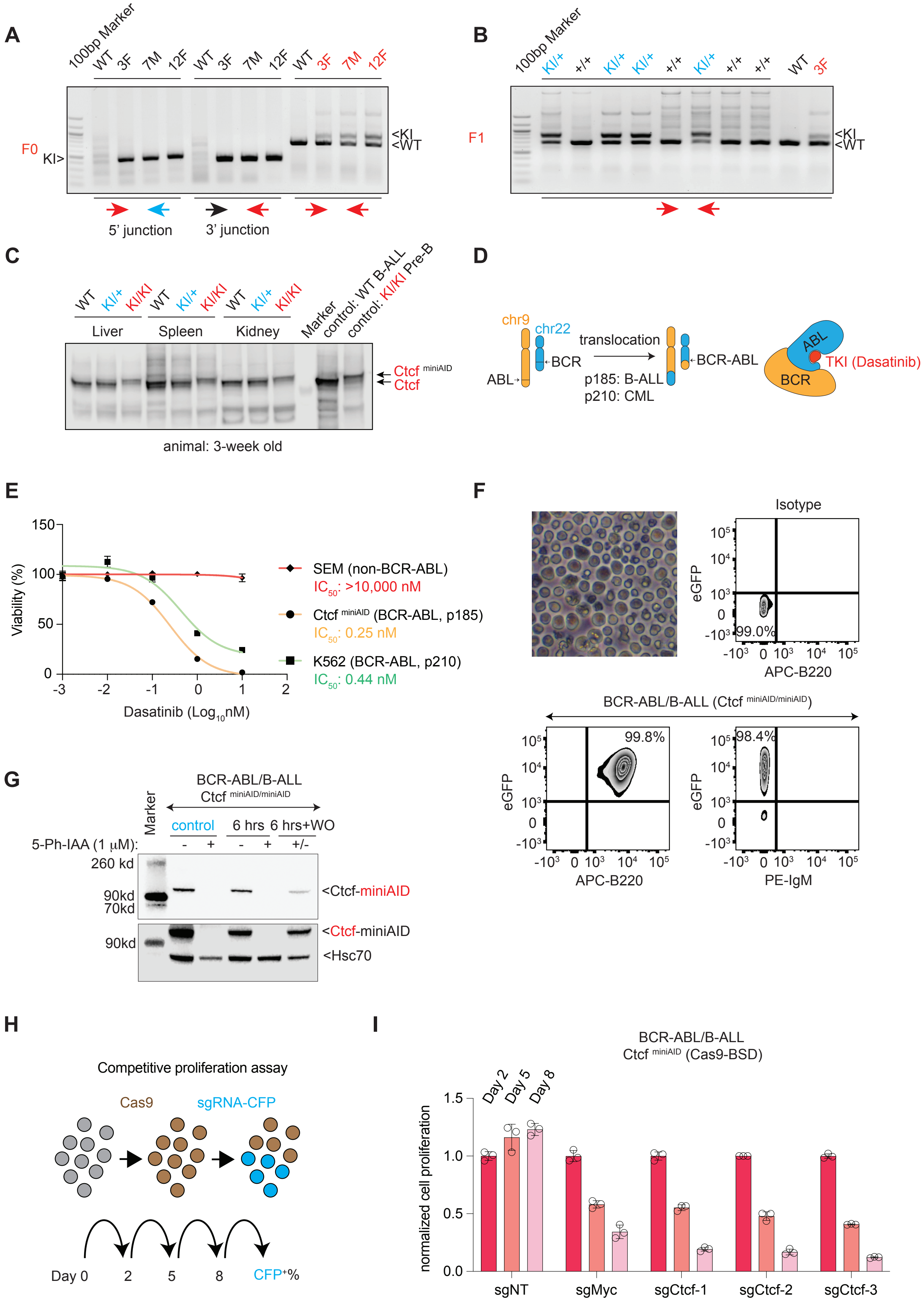

Supplementary Figure 7

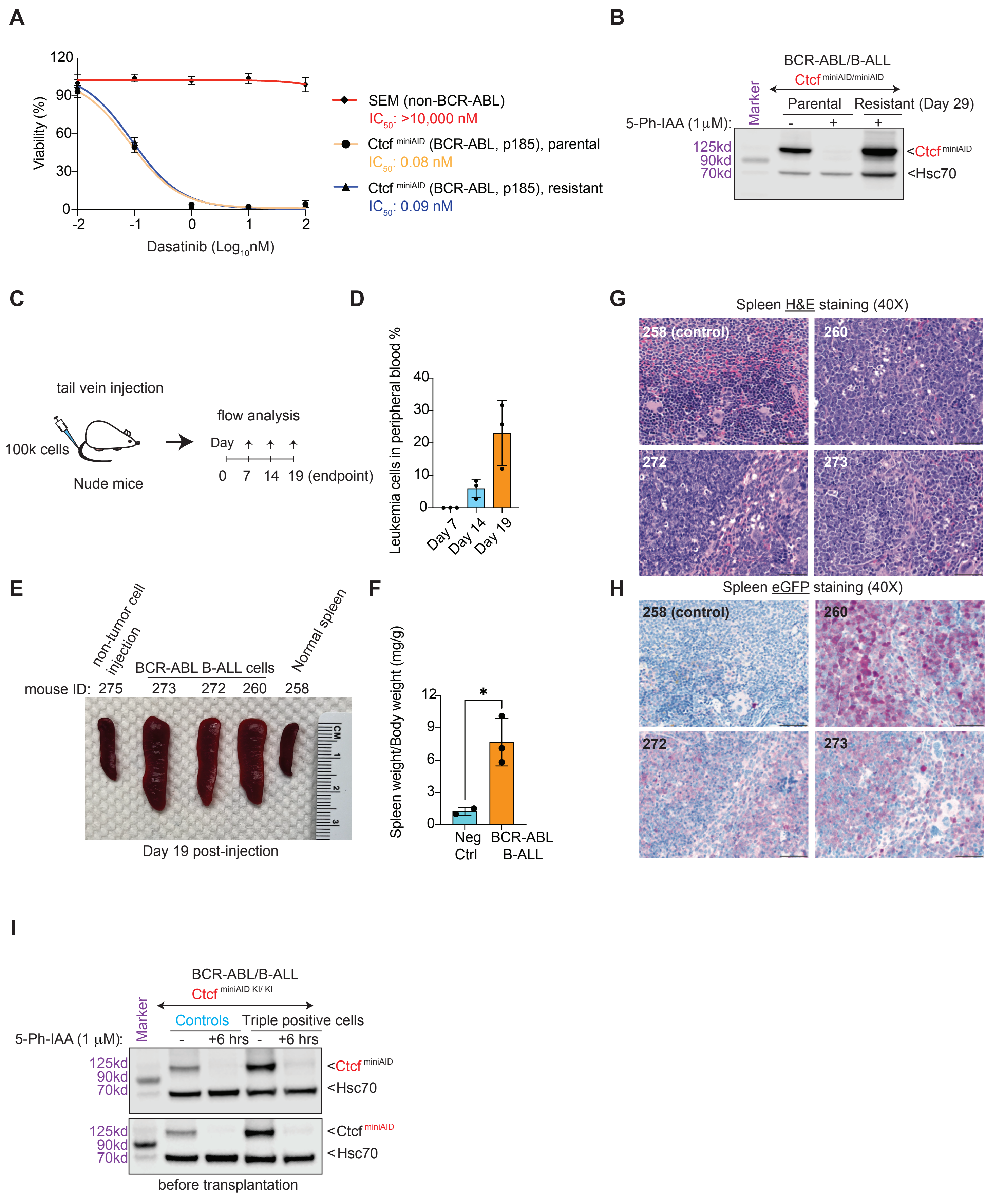

Supplement: Supplementary Figures [file mmc1.pdf]
