# Supplementary material for: Deciphering resistance mechanisms to auxin-inducible protein degradation in mammalian cells
Source: J Biol Chem. 2026 Jun 4;302(7):113232. doi: 10.1016/j.jbc.2026.113232 (PMC13325307; doi:10.1016/j.jbc.2026.113232)

Image ID: 0000667_01
Acquire Time: Dec 16, 2021 10:18:18 AM

Acquisition Information

#	Image ID	Acquire Time	Channels	Integration Times	Analysis	Image Name	Comment	Image Modifications
1	0000667_01	Dec 16, 2021 10:18:18 AM	Chemi	02:00	Western	0000667_01		

Image Display Values

Channel	Color	Minimum	Maximum	K
Chemi	Gray Scale (Black on White)	0.00000107	0.000824	0

Fig 1e CTCF short exposure

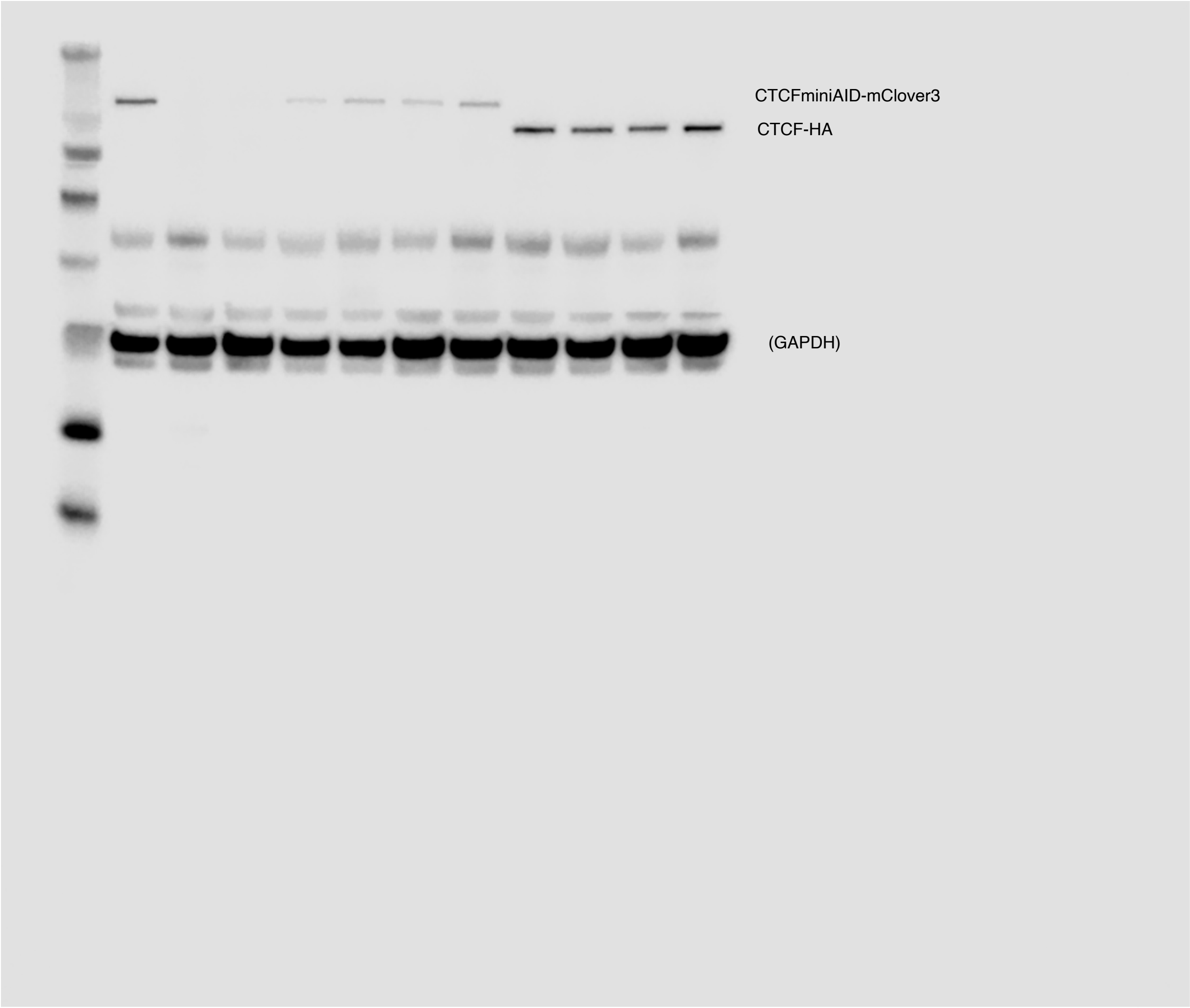

Image ID: 0000668_01
Acquire Time: Dec 16, 2021 10:23:43 AM

Acquisition Information

#	Image ID	Acquire Time	Channels	Integration Times	Analysis	Image Name	Comment	Image Modifications
1	0000668_01	Dec 16, 2021 10:23:43 AM	Chemi	09:50	Western	0000668_01		

Image Display Values

Channel	Color	Minimum	Maximum	K
Chemi	Gray Scale (Black on White)	0.000000298	0.00352	1

Fig 1e CTCF long exposure

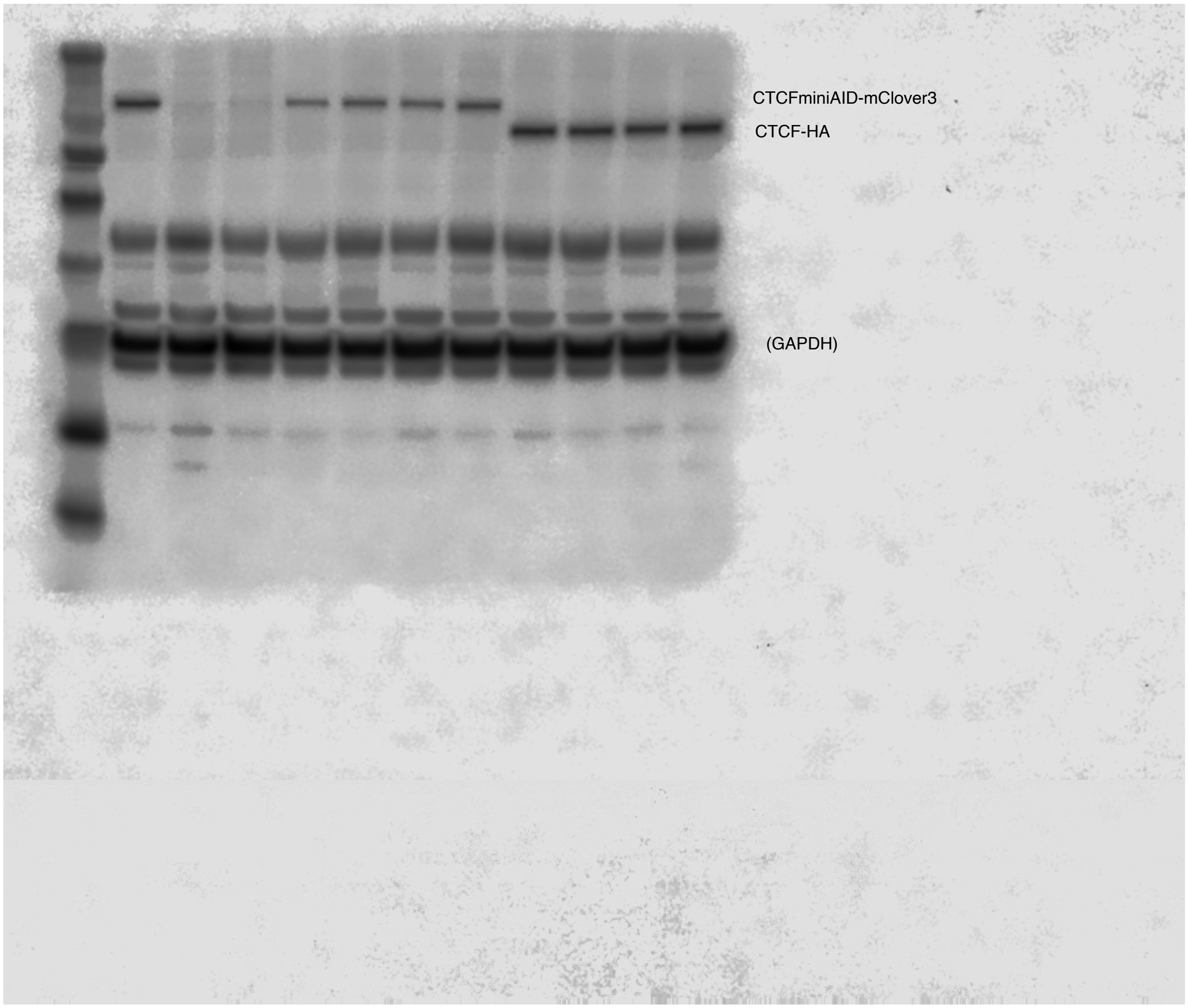

Image ID: 0000667_01
Acquire Time: Dec 16, 2021 10:18:18 AM

Acquisition Information

#	Image ID	Acquire Time	Channels	Integration Times	Analysis	Image Name	Comment	Image Modifications
1	0000667_01	Dec 16, 2021 10:18:18 AM	Chemi	02:00	Western	0000667_01		

Image Display Values

Channel	Color	Minimum	Maximum	K
Chemi	Gray Scale (Black on White)	0.0000349	0.00369	0

Fig 1 e WB GAPDH

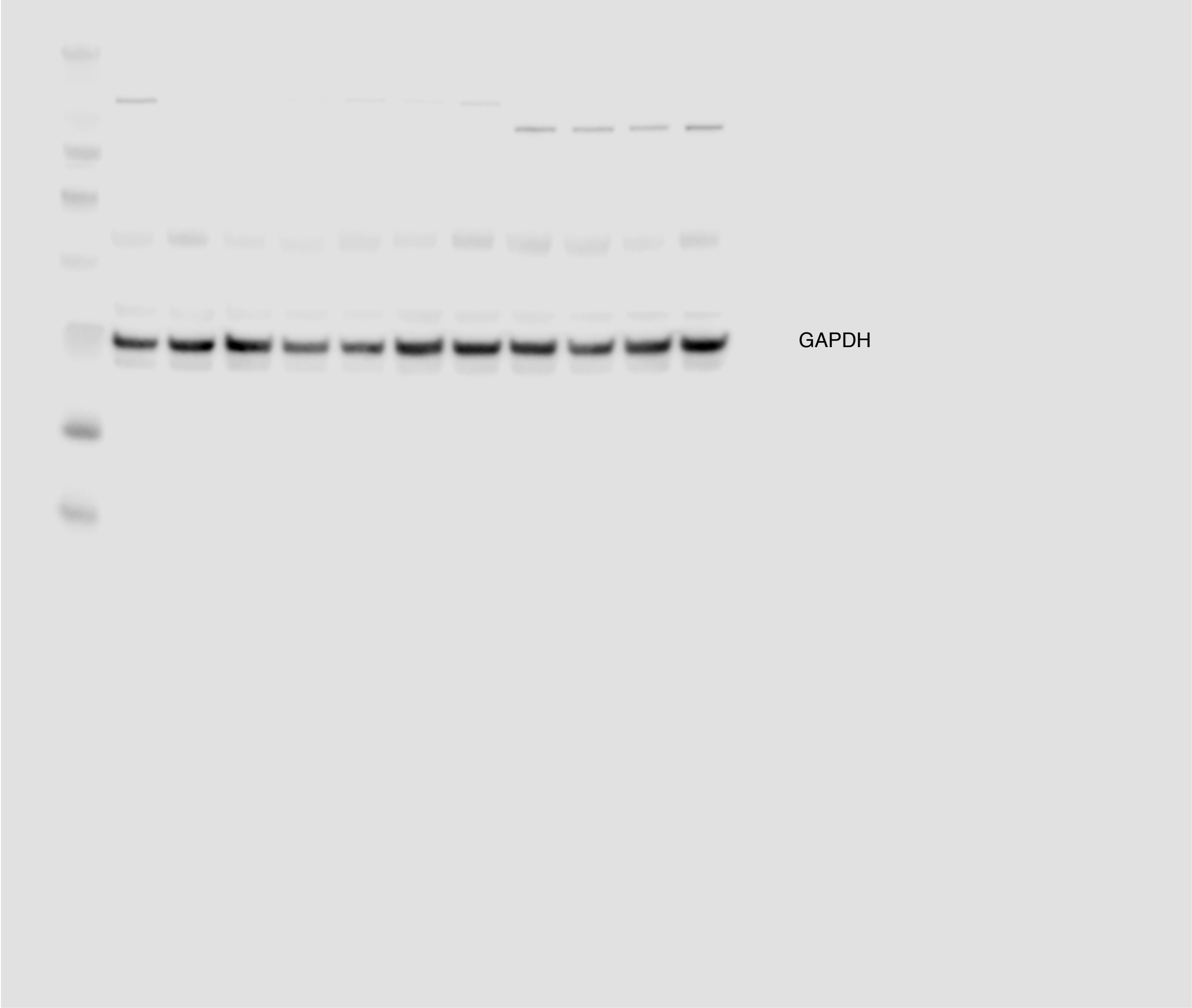

Image ID: 0003303_01
Acquire Time: Jan 11, 2024 9:01:52 AM

Acquisition Information

#	Image ID	Acquire Time	Channels	Integration Times	Analysis	Image Name	Comment	Image Modifications
1	0003303_01	Jan 11, 2024 9:01:52 AM	Chemi	03:05	Manual	0003303_01		

Image Display Values

Channel	Color	Minimum	Maximum	K
Chemi	Gray Scale (Black on White)	0.00000167	0.0131	0.05

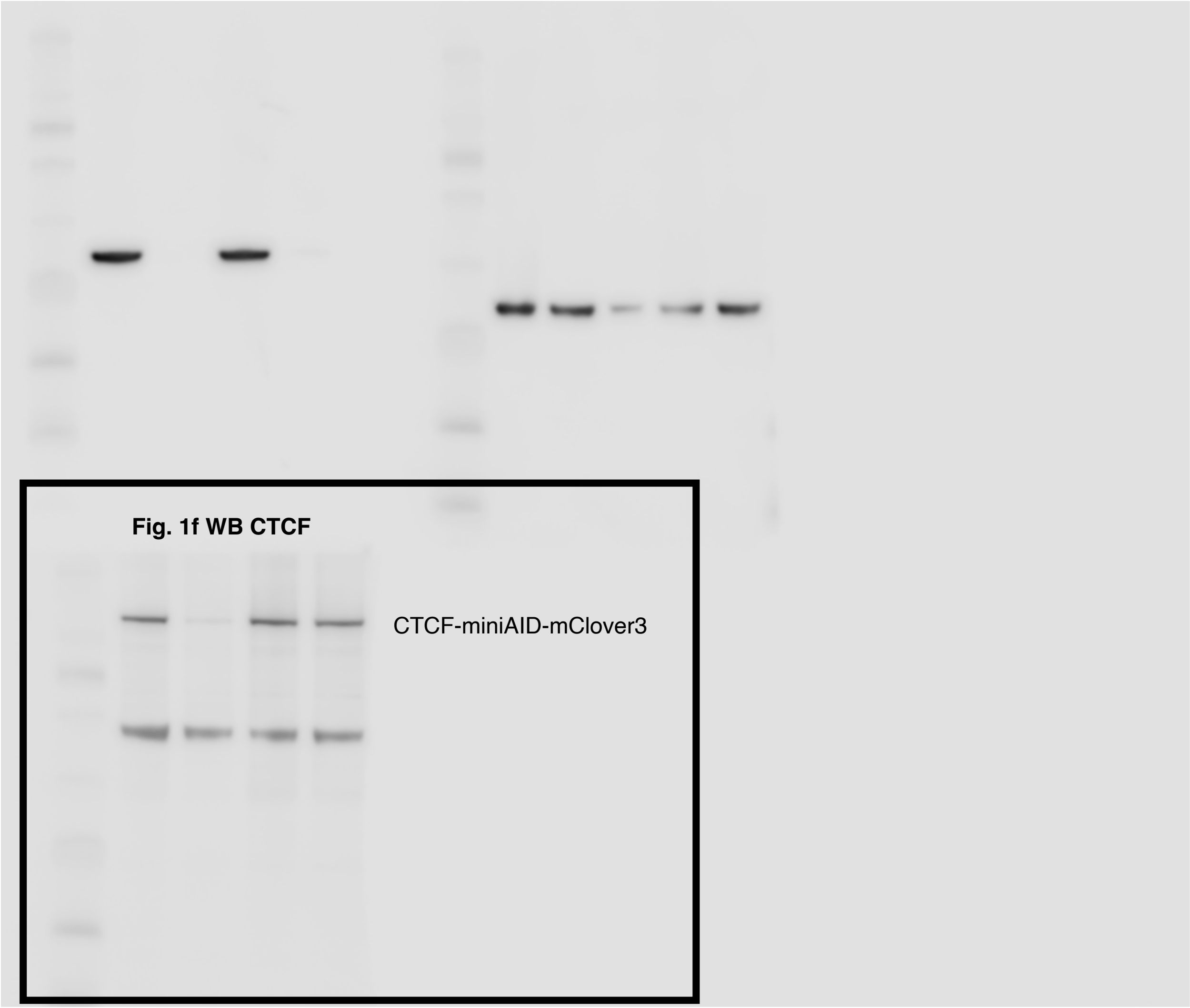

Image ID: 0003302_01
Acquire Time: Jan 11, 2024 8:49:36 AM

Acquisition Information

#	Image ID	Acquire Time	Channels	Integration Times	Analysis	Image Name	Comment	Image Modifications
1	0003302_01	Jan 11, 2024 8:49:36 AM	Chemi	04:10	Manual	0003302_01		

Image Display Values

Channel	Color	Minimum	Maximum	K
Chemi	Gray Scale (Black on White)	0.00000328	0.000378	0

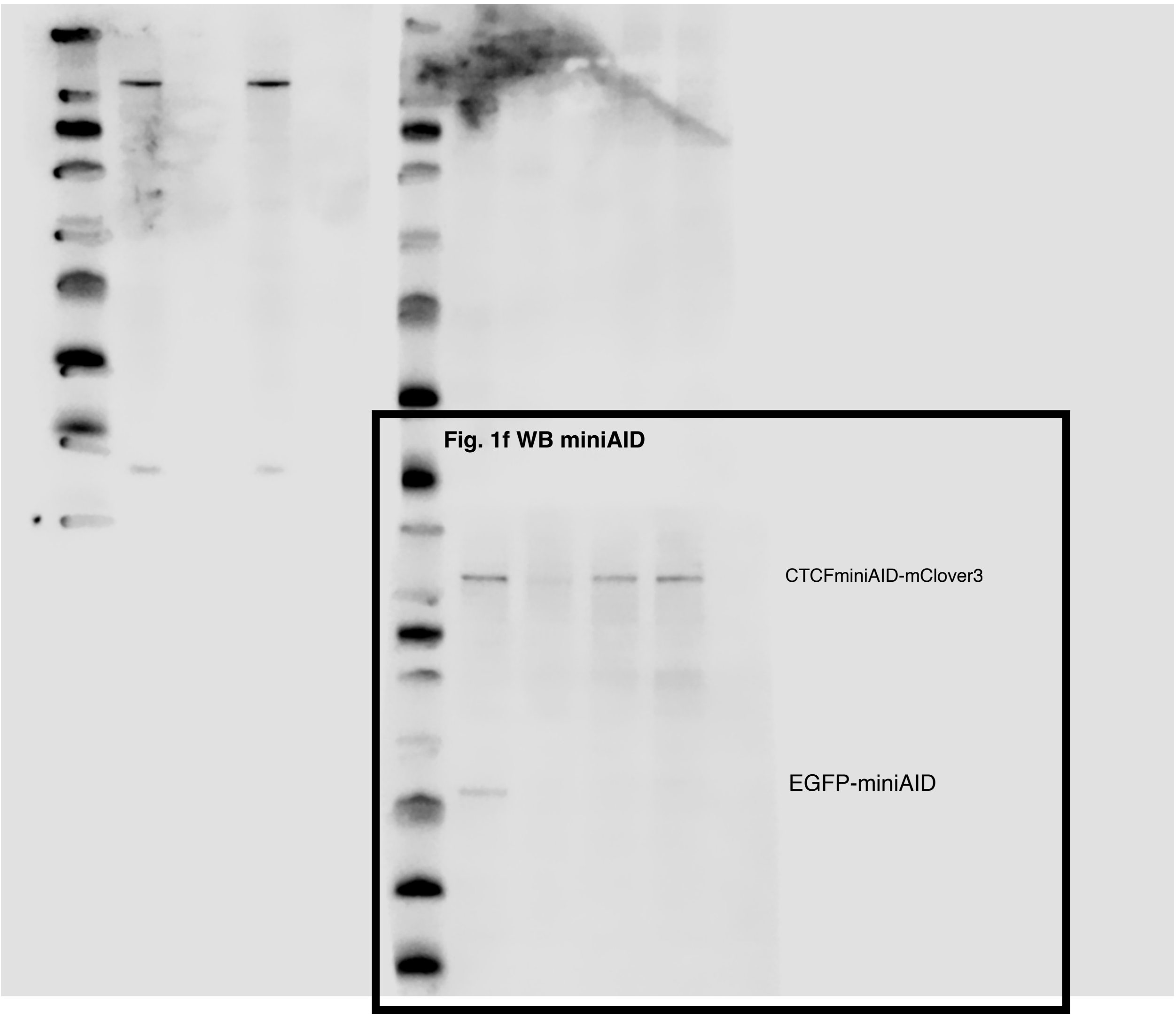

Image ID: 0003336_02
Acquire Time: Jan 30, 2024 11:34:07 AM

Acquisition Information

#	Image ID	Acquire Time	Channels	Integration Times	Analysis	Image Name	Comment
1	0003336_02	Jan 30, 2024 11:34:07 AM	Chemi	04:10	Manual	0003336_02	

Image Display Values

Channel	Color	Minimum	Maximum	K
Chemi	Gray Scale (Black on White)	0.0000116	0.000464	0

Fig. 1f WB EGFP/mClover3

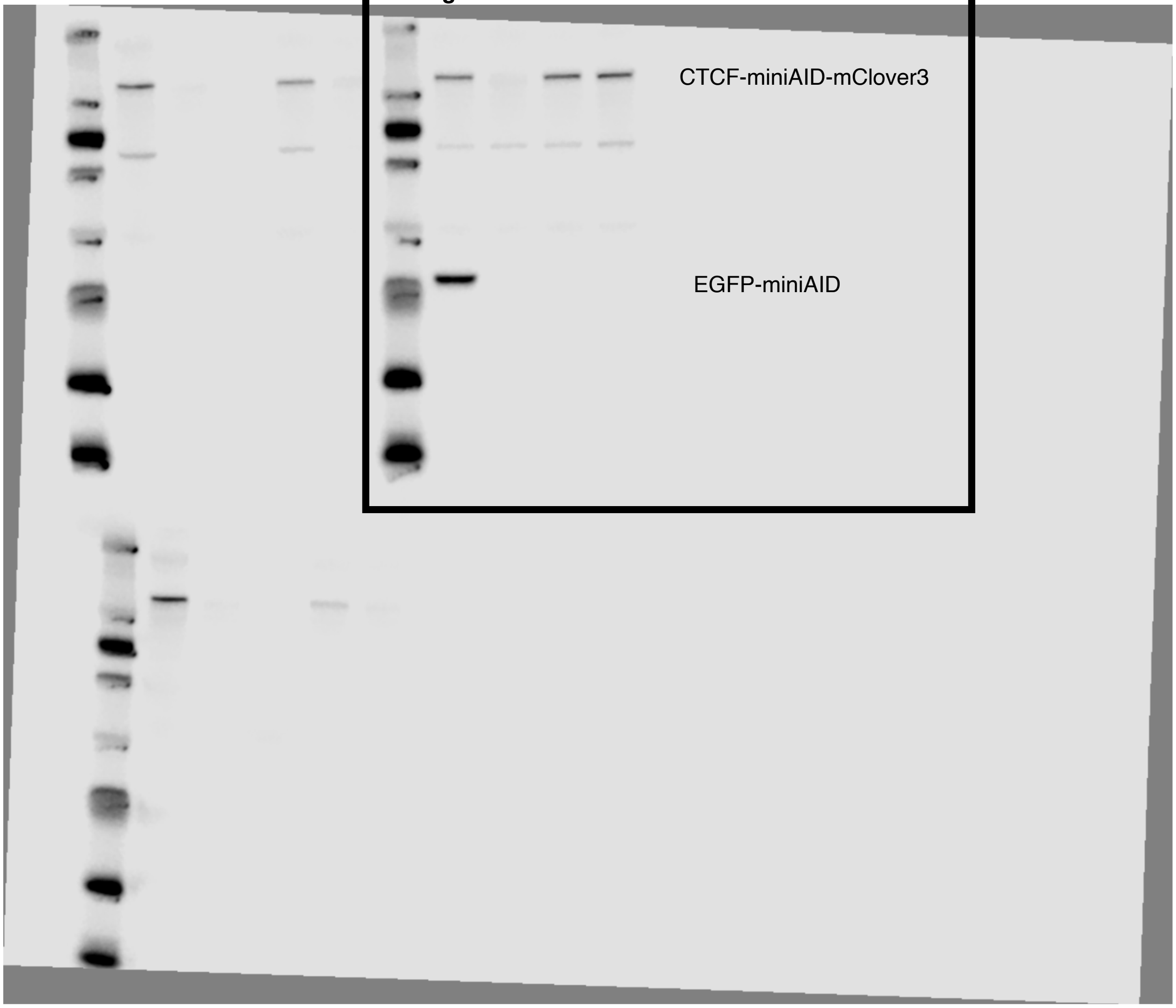

LI-COR®

Image ID: 0003308_01
Acquire Time: Jan 11, 2024 2:38:24 PM

Acquisition Information

#	Image ID	Acquire Time	Channels	Integration Times	Analysis	Image Name	Comment	Image Modifications
1	0003308_01	Jan 11, 2024 2:38:24 PM	Chemi	03:05	Manual	0003308_01		

Image Display Values

Channel	Color	Minimum	Maximum	K
Chemi	Gray Scale (Black on White)	0.00000179	0.00961	0

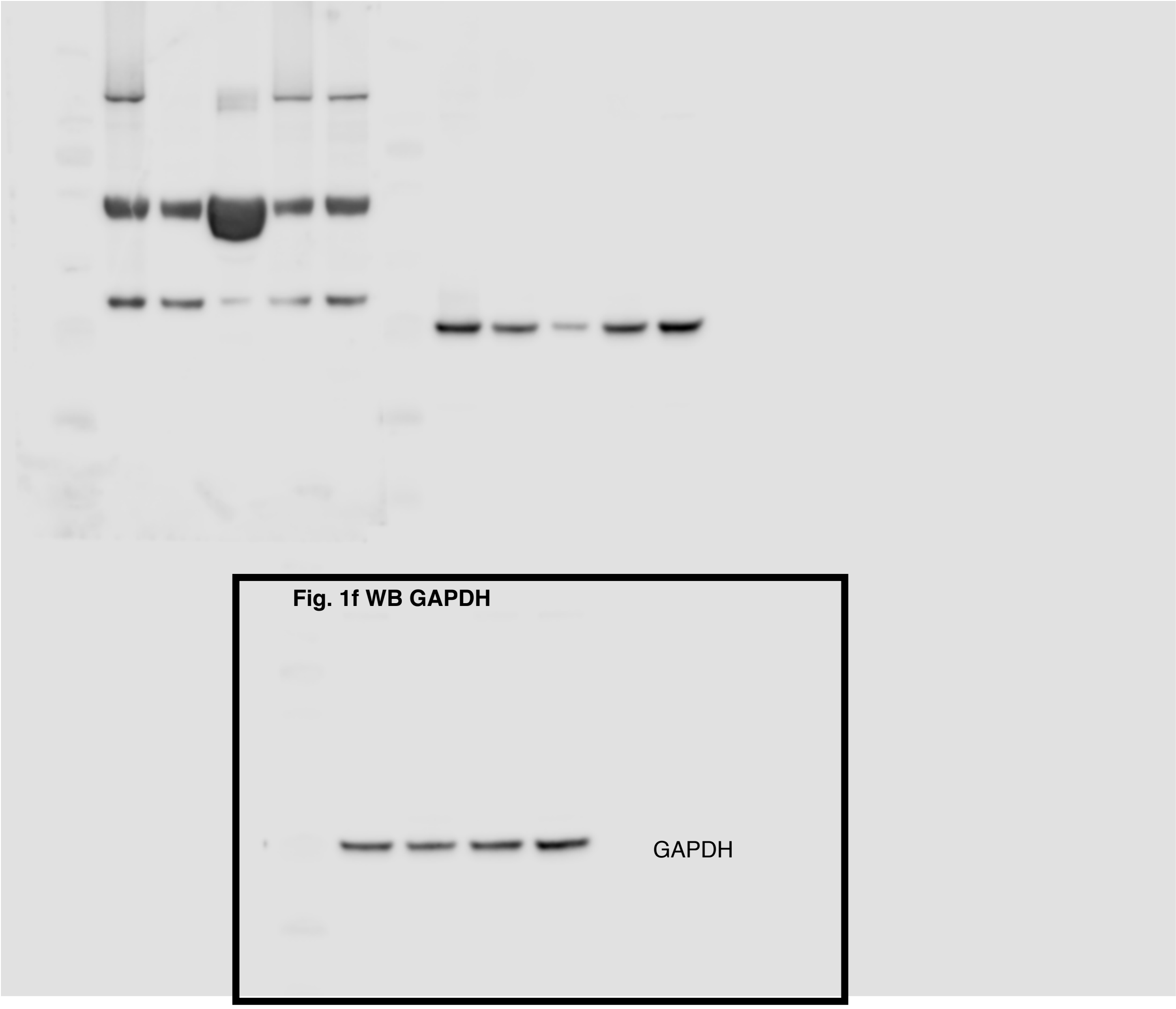

Image ID: 0005025_01
Acquire Time: Feb 4, 2025 11:55:03 AM

Acquisition Information

#	Image ID	Acquire Time	Channels	Integration Times	Analysis	Image Name	Comment	Image Modifications
1	0005025_01	Feb 4, 2025 11:55:03 AM	Chemi	01:52	Manual	0005025_01		

Image Display Values

Channel	Color	Minimum	Maximum	K
Chemi	Gray Scale (Black on White)	0.00000137	0.000762	0

Fig. 1h WB CTCF

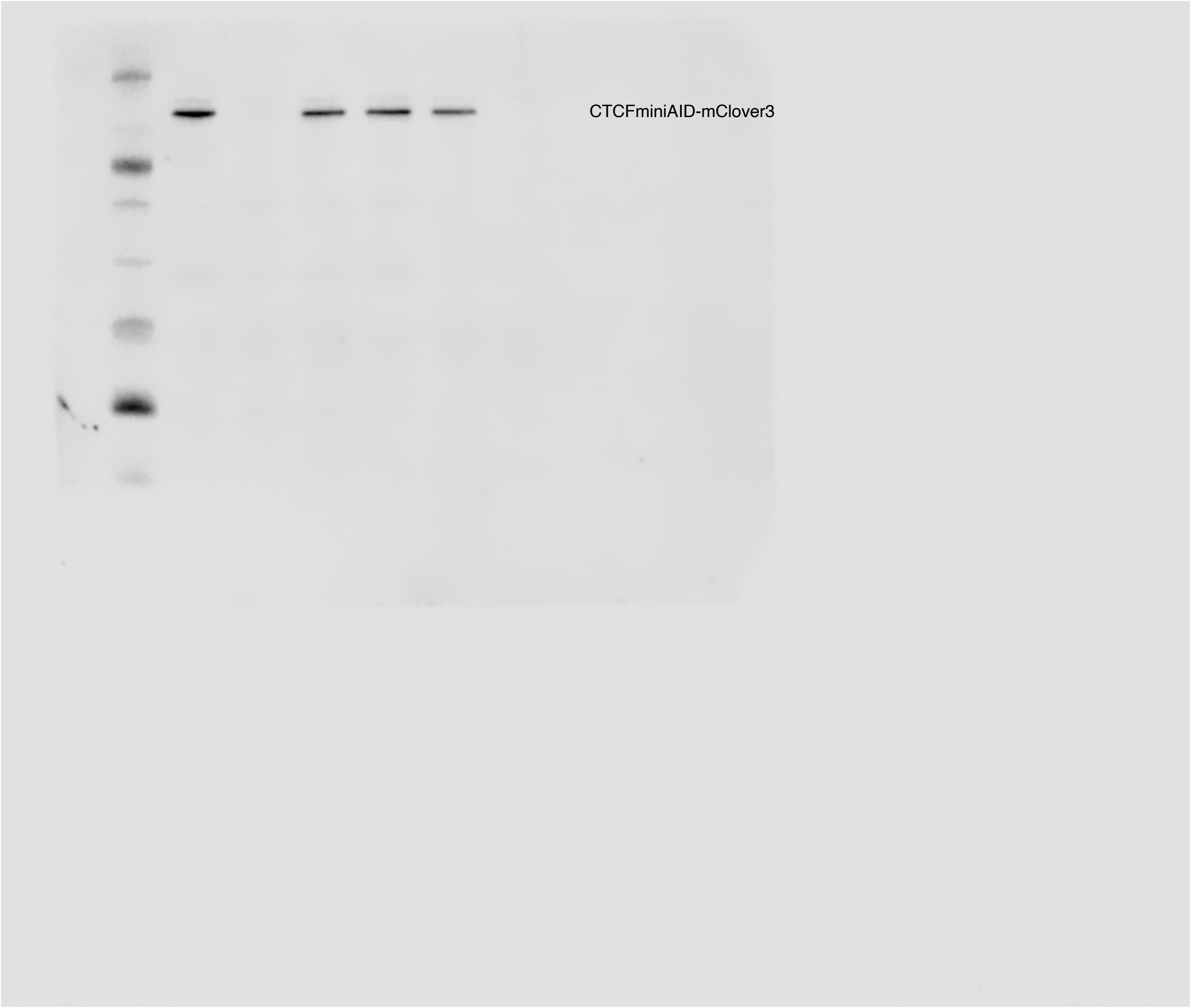

Image ID: 0005037_01
Acquire Time: Feb 5, 2025 10:14:23 AM

Acquisition Information

#	Image ID	Acquire Time	Channels	Integration Times	Analysis	Image Name	Comment	Image Modifications
1	0005037_01	Feb 5, 2025 10:14:23 AM	Chemi	00:30	Manual	0005037_01		

Image Display Values

Channel	Color	Minimum	Maximum	K
Chemi	Gray Scale (Black on White)	0.00000352	0.00712	0.13

Fig. 1h WB GAPDH

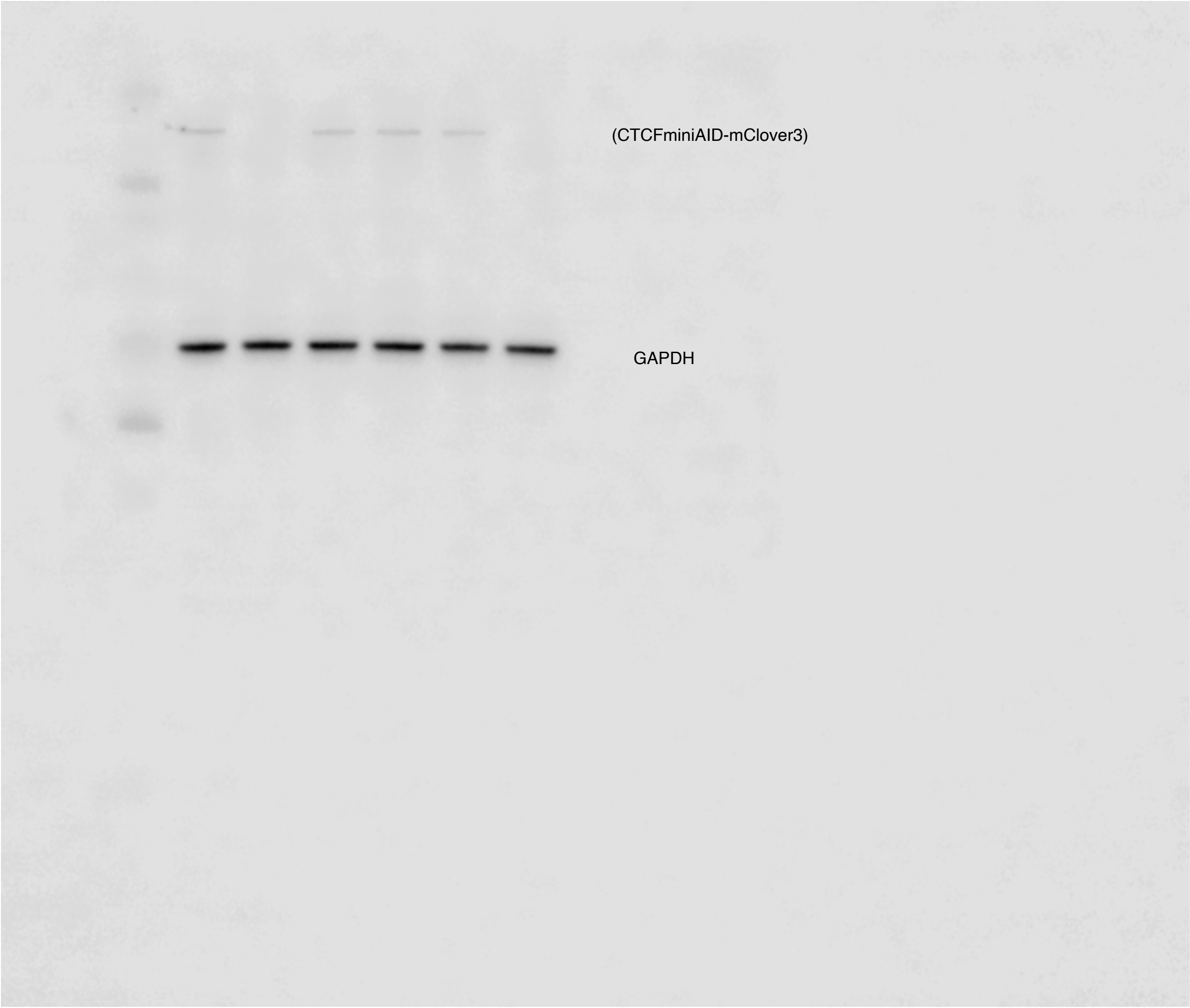

Image ID: 0003345_01
Acquire Time: Feb 1, 2024 11:43:57 AM

Acquisition Information

#	Image ID	Acquire Time	Channels	Integration Times	Analysis	Image Name	Comment	Image Modifications
1	0003345_01	Feb 1, 2024 11:43:57 AM	Chemi	02:05	Manual	0003345_01		

Image Display Values

Channel	Color	Minimum	Maximum	K
Chemi	Gray Scale (Black on White)	0.00000513	0.00983	0

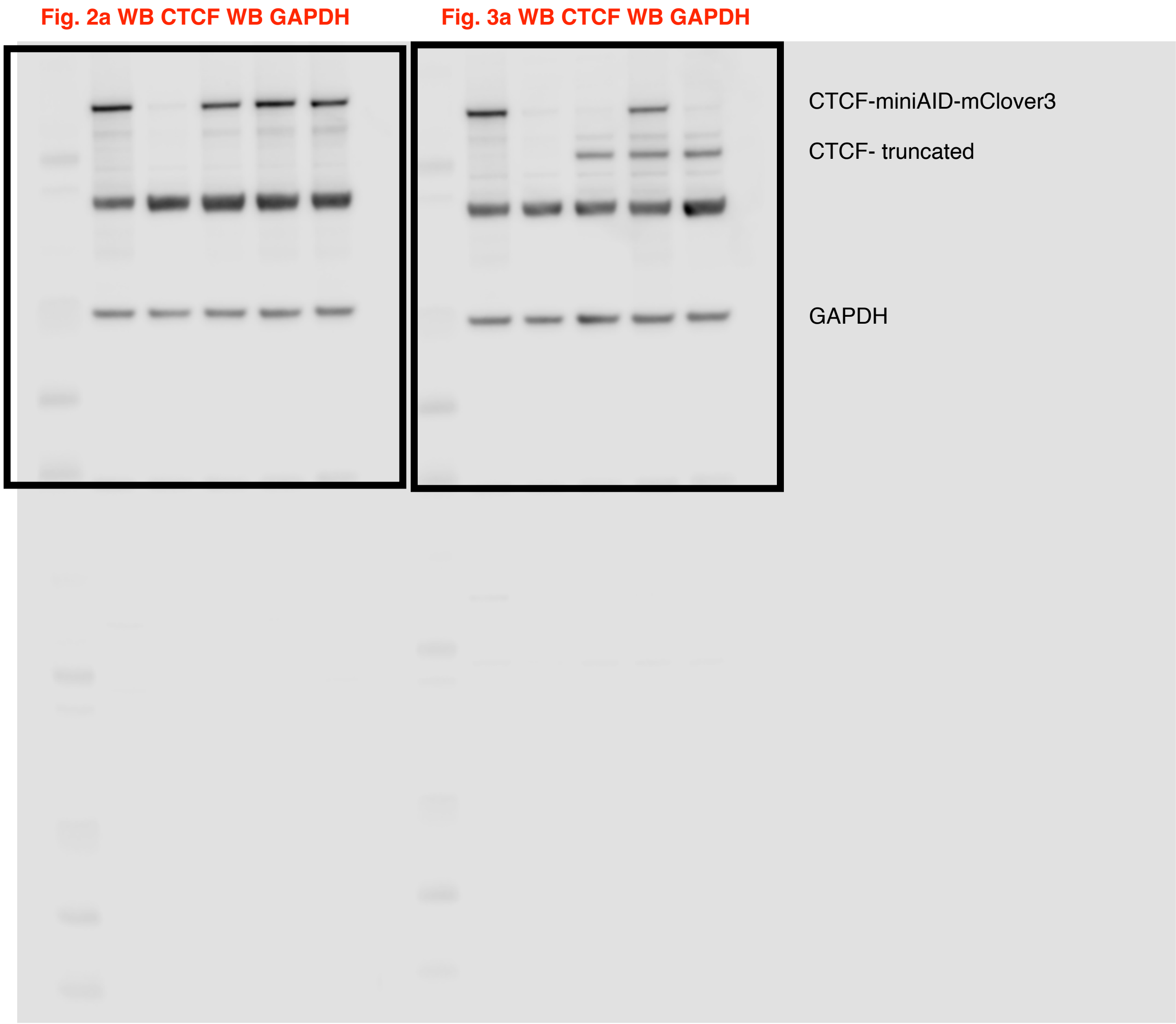

Image ID: 0003346_02
Acquire Time: Feb 1, 2024 11:48:46 AM

Acquisition Information

#	Image ID	Acquire Time	Channels	Integration Times	Analysis	Image Name	Comment
1	0003346_02	Feb 1, 2024 11:48:46 AM	Chemi	04:30	Manual	0003346_02	

Image Display Values

Channel	Color	Minimum	Maximum	K
Chemi	Gray Scale (Black on White)	0.00000948	0.000215	0

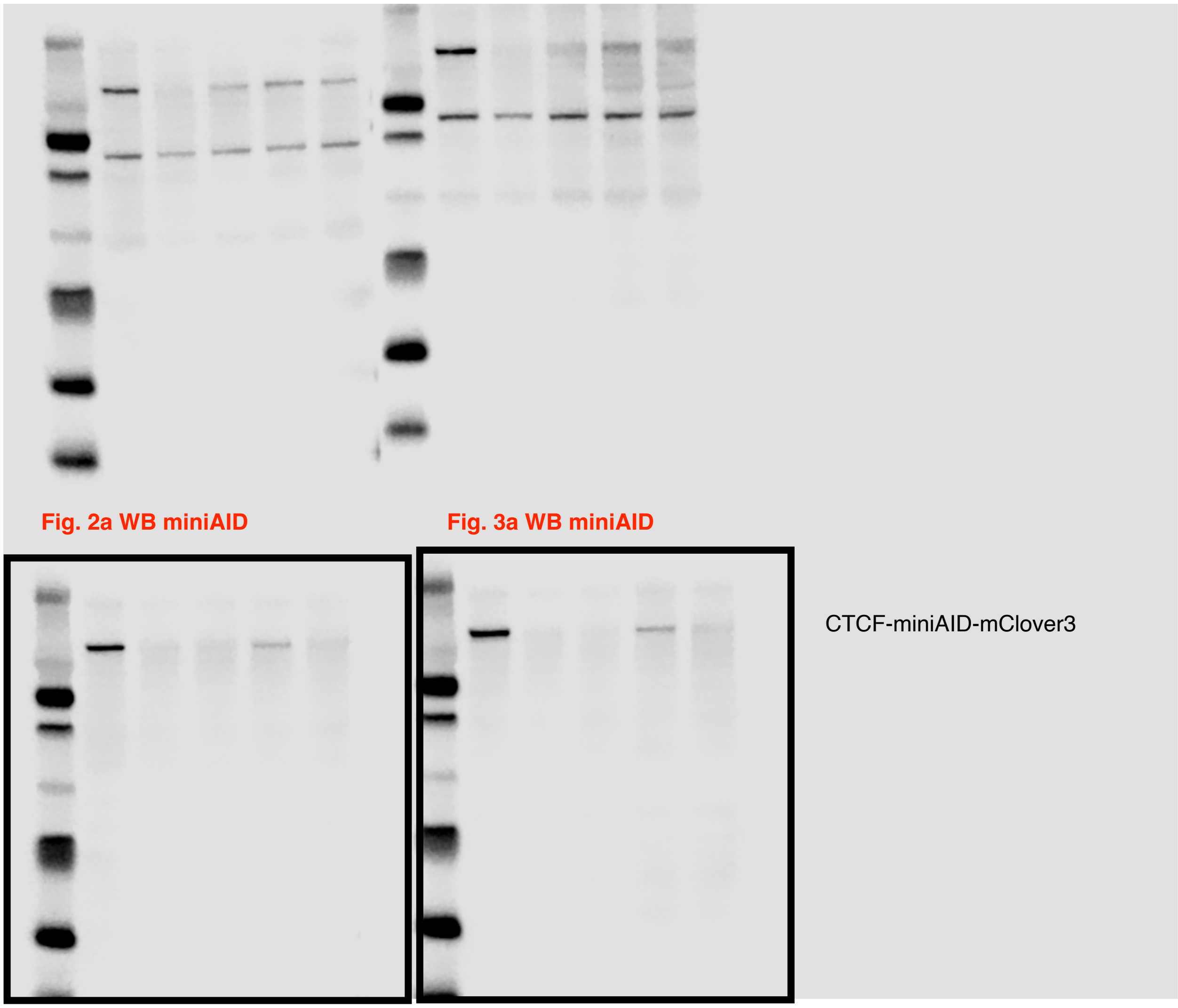

Image ID: 0004948_01
Acquire Time: Jan 22, 2025 10:15:38 AM

Acquisition Information

#	Image ID	Acquire Time	Channels	Integration Times	Analysis	Image Name	Comment	Image Modifications
1	0004948_01	Jan 22, 2025 10:15:38 AM	Chemi	01:58	Manual	0004948_01		

Image Display Values

Channel	Color	Minimum	Maximum	K
Chemi	Gray Scale (Black on White)	0.00000167	0.000457	0.14

Fig. 2f WB EGFP

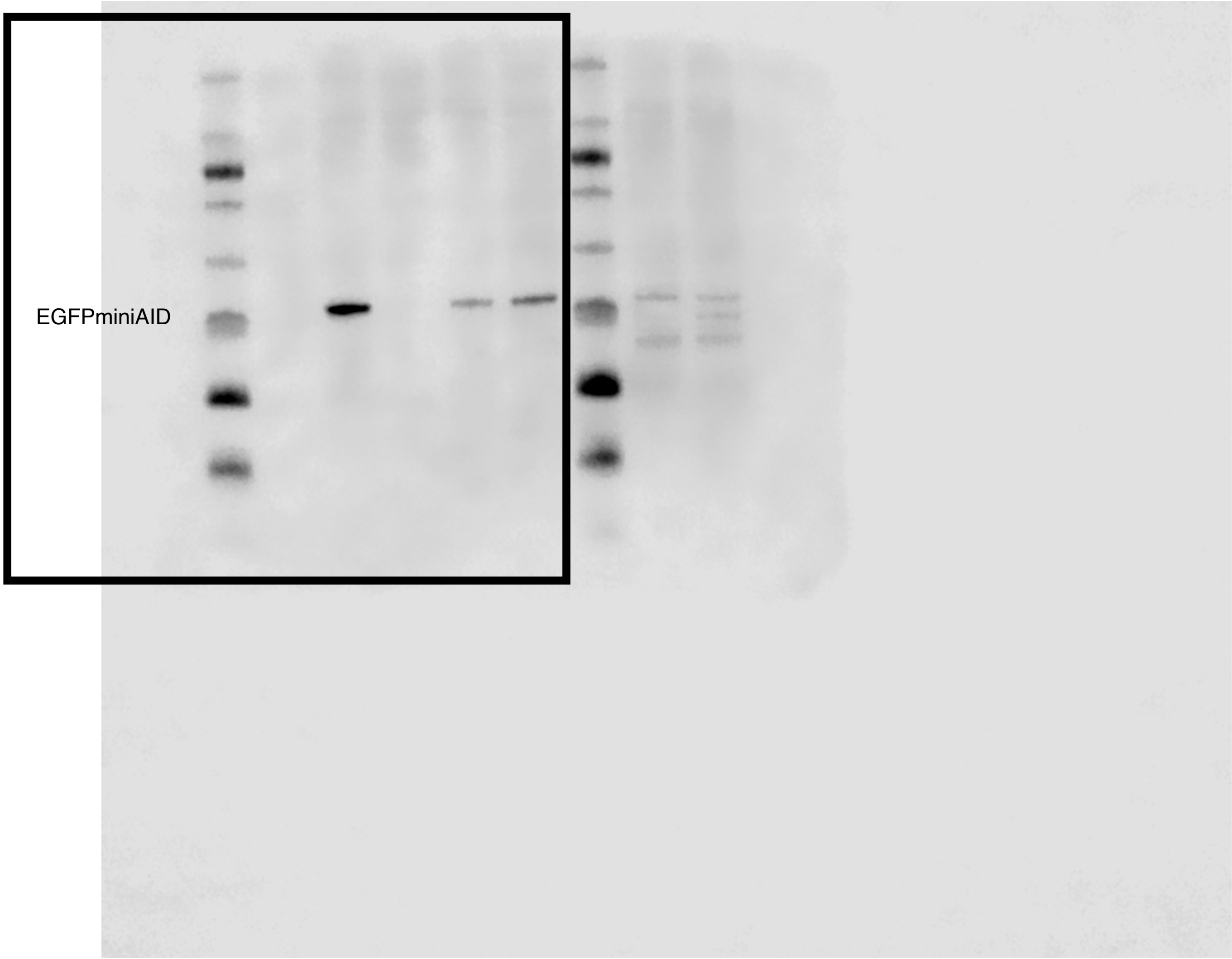

Image ID: 0004947_01
Acquire Time: Jan 22, 2025 10:05:07 AM

Acquisition Information

#	Image ID	Acquire Time	Channels	Integration Times	Analysis	Image Name	Comment	Image Modifications
1	0004947_01	Jan 22, 2025 10:05:07 AM	Chemi	01:58	Manual	0004947_01		

Image Display Values

Channel	Color	Minimum	Maximum	K
Chemi	Gray Scale (Black on White)	0.00000221	0.000460	0.34

Fig. 2f WB miniAID

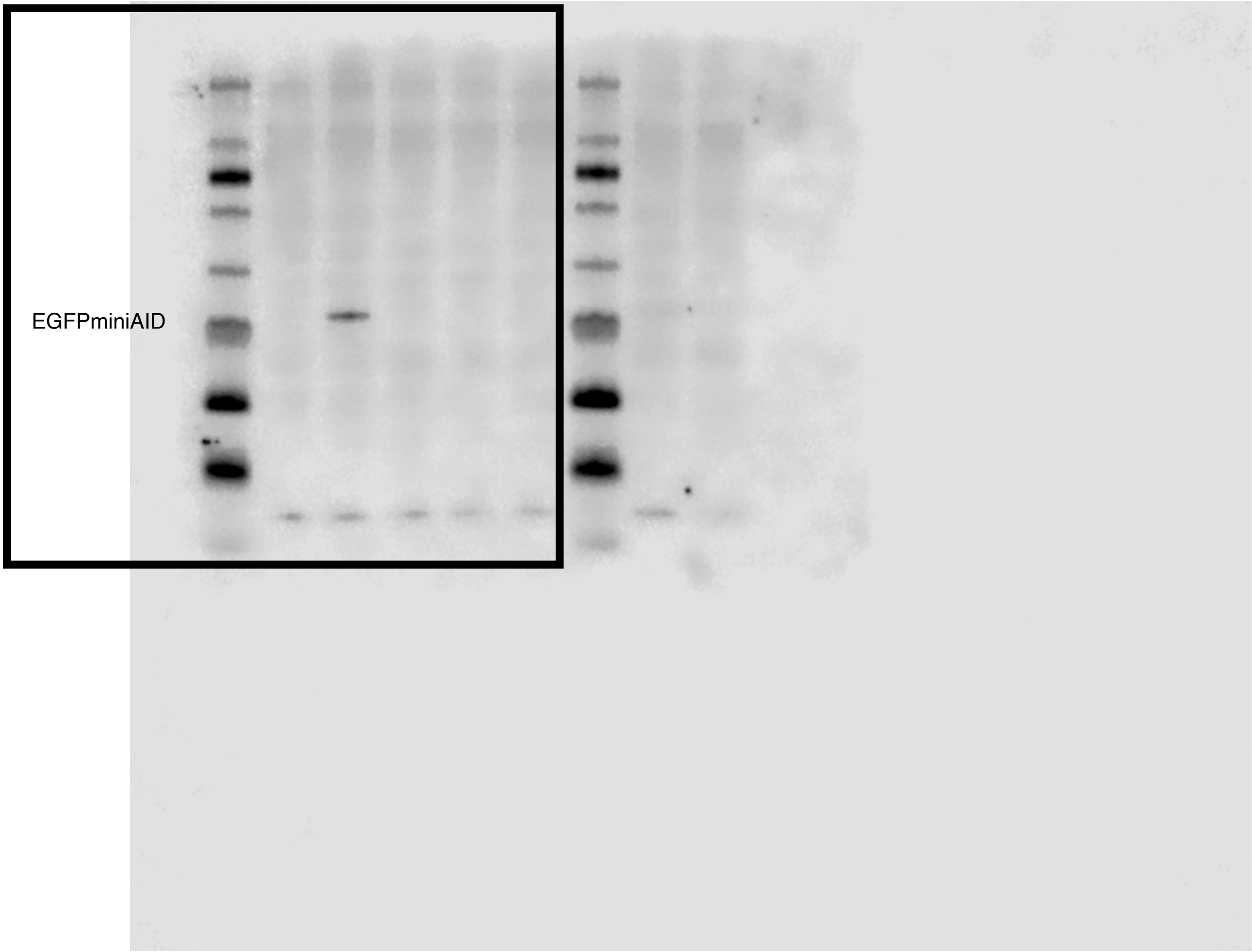

Image ID: 0004959_01
Acquire Time: Jan 23, 2025 11:45:27 AM

Acquisition Information

#	Image ID	Acquire Time	Channels	Integration Times	Analysis	Image Name	Comment	Image Modifications
1	0004959_01	Jan 23, 2025 11:45:27 AM	Chemi	01:06	Manual	0004959_01		

Image Display Values

Channel	Color	Minimum	Maximum	K
Chemi	Gray Scale (Black on White)	0.00000244	0.00872	0.1

Fig. 2f WB HSC70

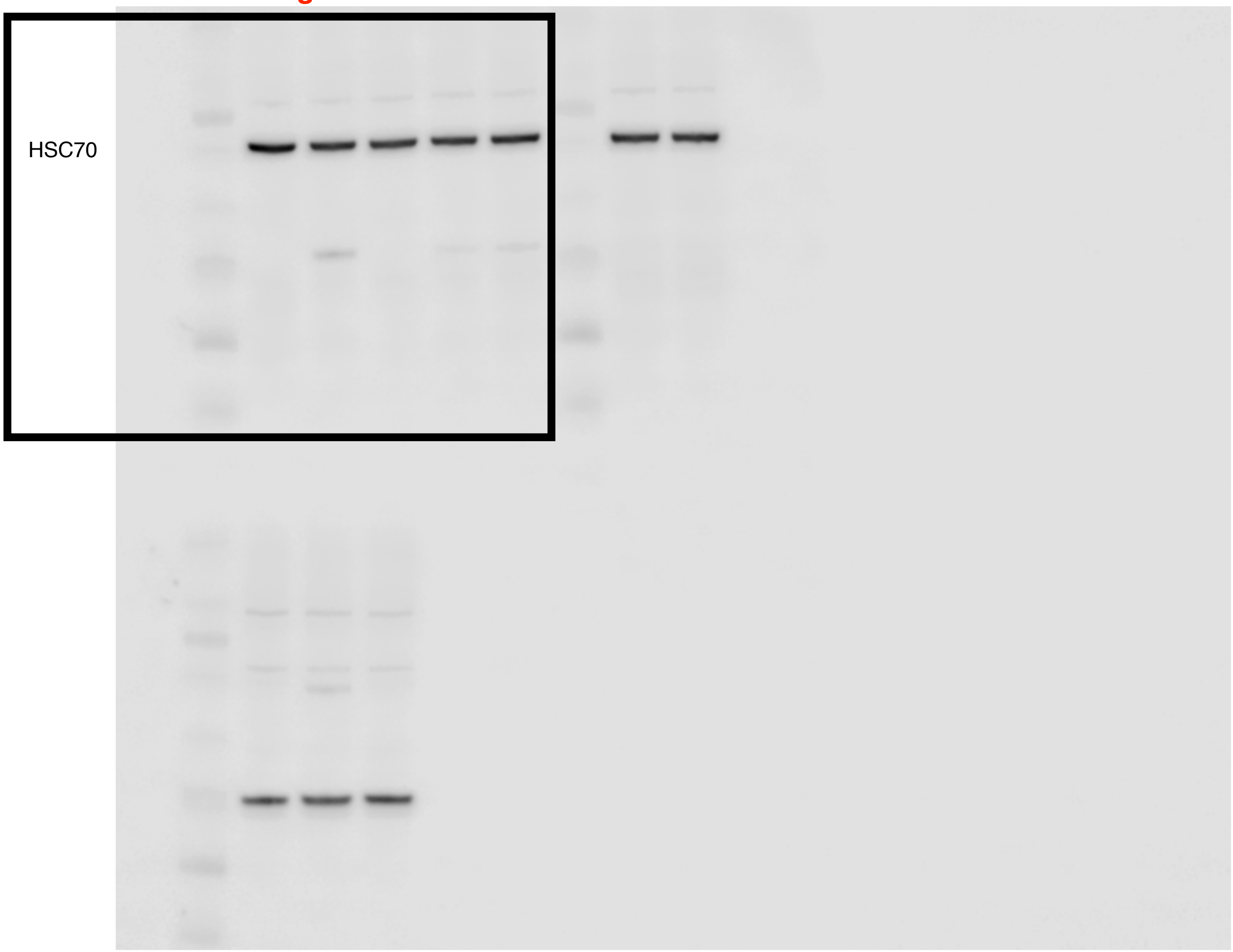

Fig. 4c genotyping PCR

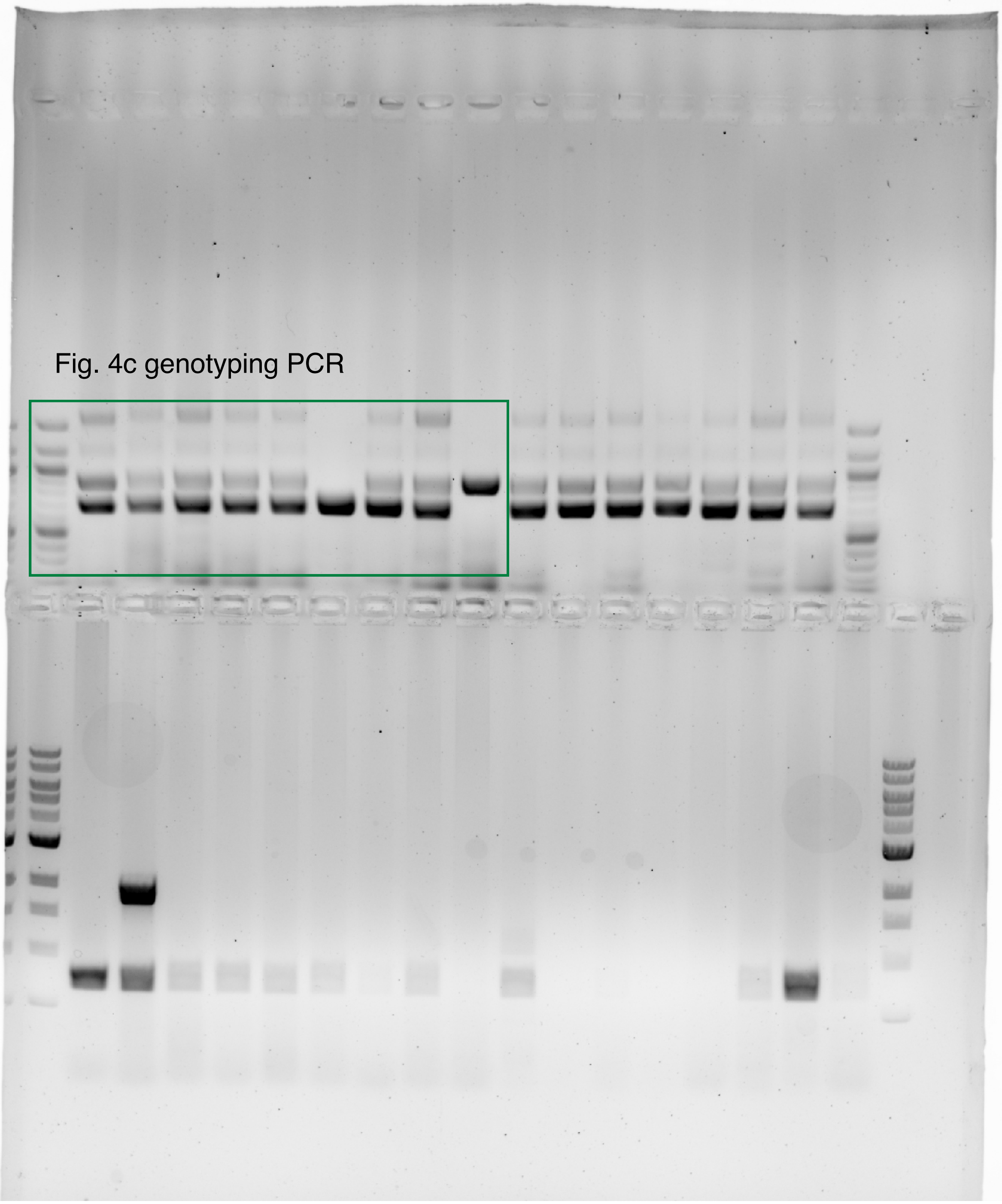

Image ID: 0004382_02
Acquire Time: Sep 20, 2024 2:04:43 PM

Acquisition Information

#	Image ID	Acquire Time	Channels	Integration Times	Analysis	Image Name	Comment
1	0004382_02	Sep 20, 2024 2:04:43 PM	Chemi	02:00	Manual	0004382_02	BMC PreB-miniAID

Image Display Values

Channel	Color	Minimum	Maximum	K
Chemi	Gray Scale (Black on White)	0.00000858	0.000212	0

Fig. 4e miniAID

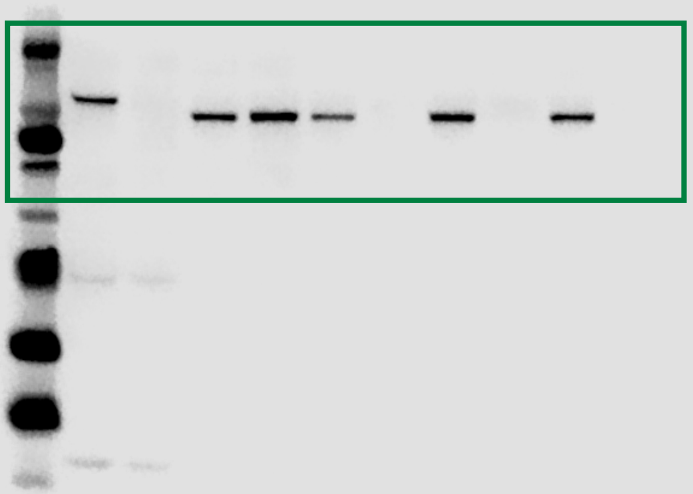

Image ID: 0004381_02
Acquire Time: Sep 20, 2024 1:59:38 PM

Acquisition Information

#	Image ID	Acquire Time	Channels	Integration Times	Analysis	Image Name	Comment
1	0004381_02	Sep 20, 2024 1:59:38 PM	Chemi	02:00	Manual	0004381_02	BMC PreB-CTCF

Image Display Values

Channel	Color	Minimum	Maximum	K
Chemi	Gray Scale (Black on White)	0.00000143	0.0111	0

Fig. 4e CTCF/Ctcf

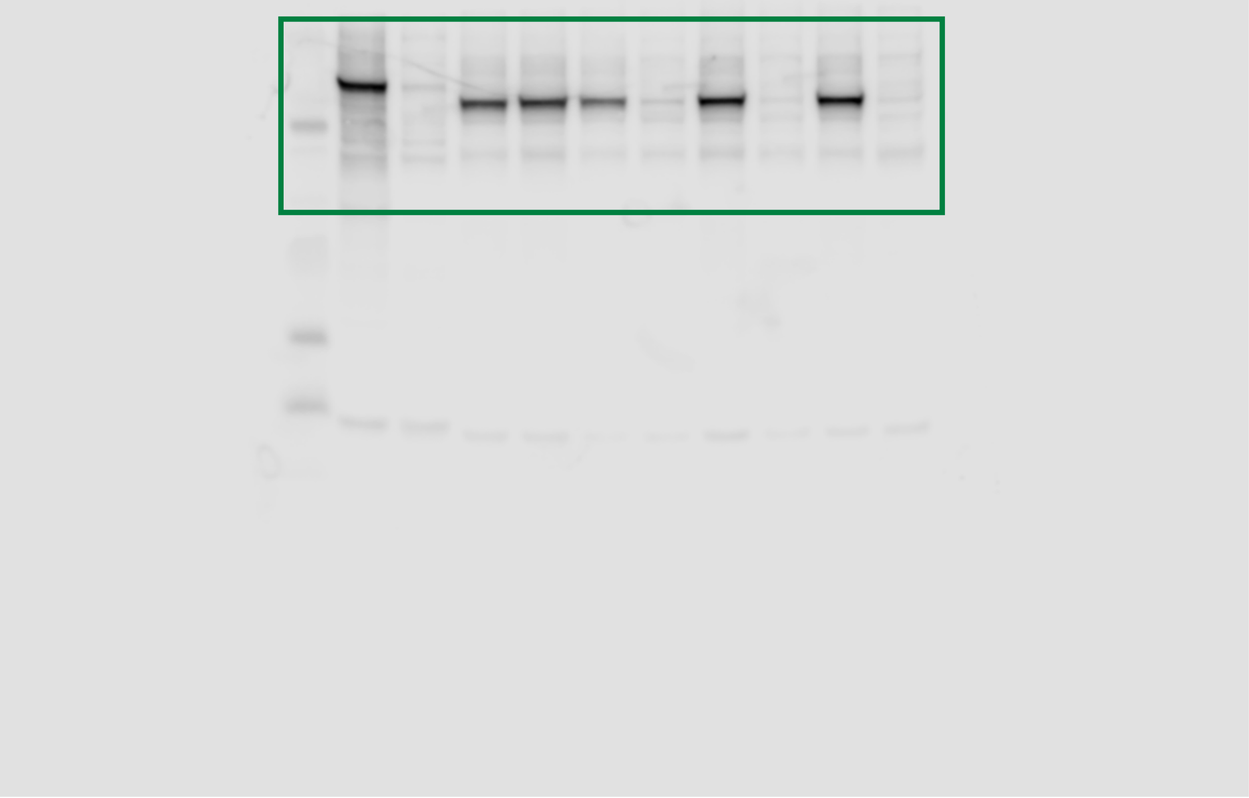

Image ID: 0004385_02
Acquire Time: Sep 20, 2024 5:15:28 PM

Acquisition Information

#	Image ID	Acquire Time	Channels	Integration Times	Analysis	Image Name	Comment
1	0004385_02	Sep 20, 2024 5:15:28 PM	Chemi	02:00	Manual	0004385_02	CTCF-GAPDH

Image Display Values

Channel	Color	Minimum	Maximum	K
Chemi	Gray Scale (Black on White)	0.000122	0.000932	0

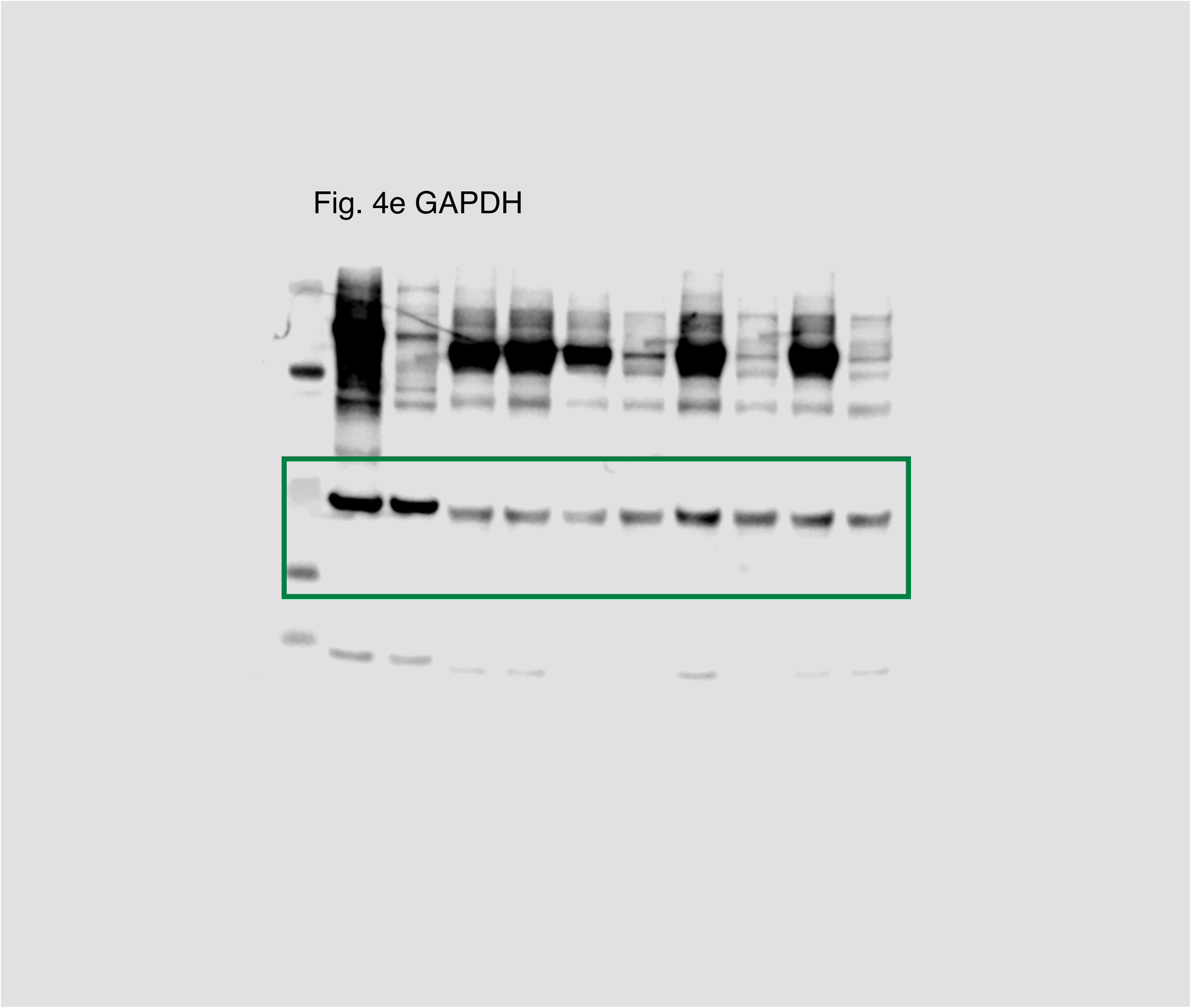

Image ID: 0004921_02
Acquire Time: Jan 8, 2025 3:21:21 PM

Acquisition Information

#	Image ID	Acquire Time	Channels	Integration Times	Analysis	Image Name	Comment
1	0004921_02	Jan 8, 2025 3:21:21 PM	Chemi	02:00	Manual	0004921_02	CTCF HSC70

Image Display Values

Channel	Color	Minimum	Maximum	K
Chemi	Gray Scale (Black on White)	0.0000677	0.00105	0

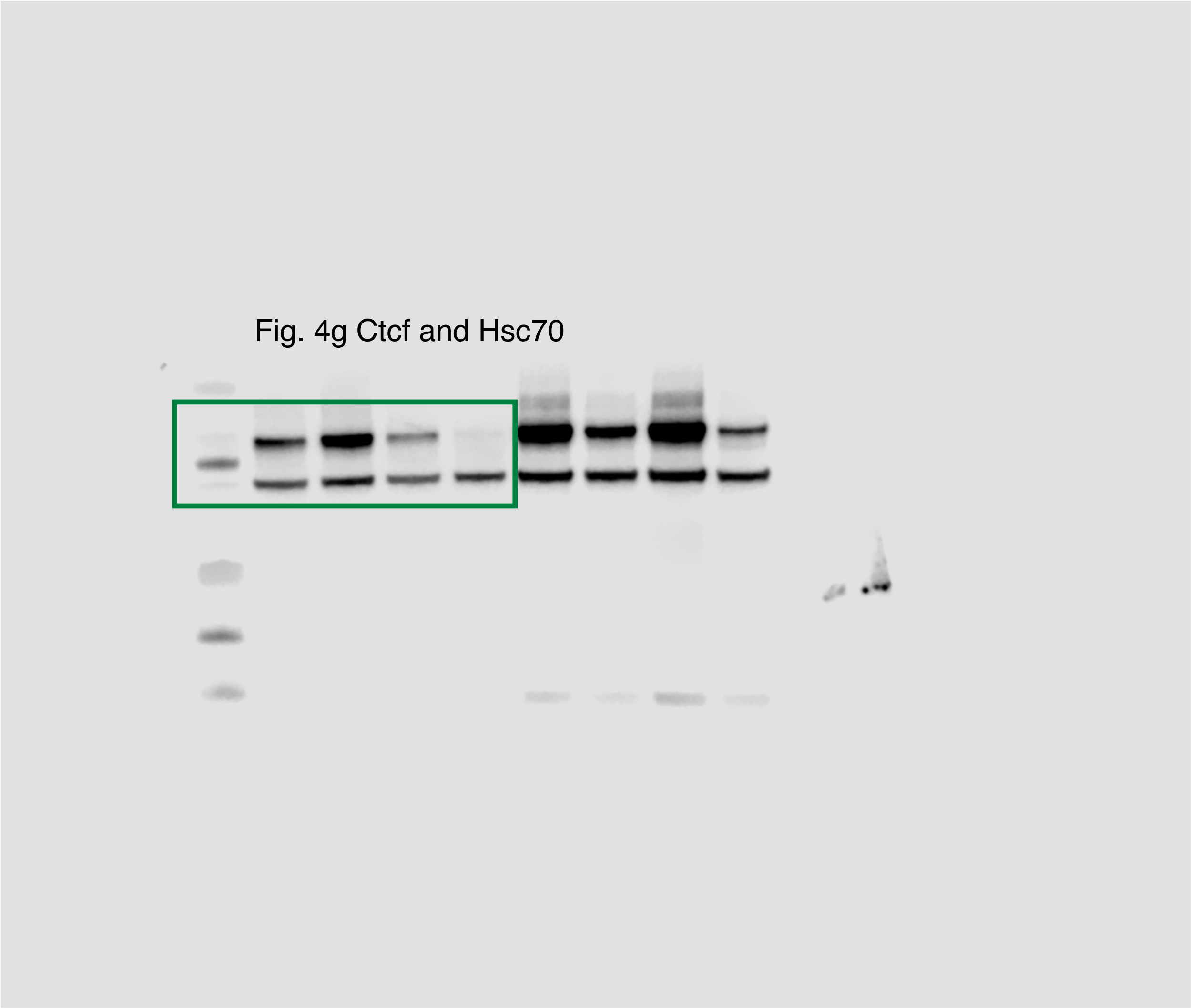

Fig. 4g Ctcf and Hsc70

Image ID: 0004916_02
Acquire Time: Jan 8, 2025 12:32:11 PM

Acquisition Information

#	Image ID	Acquire Time	Channels	Integration Times	Analysis	Image Name	Comment
1	0004916_02	Jan 8, 2025 12:32:11 PM	Chemi	02:00	Manual	0004916_02	EGFP for LT

Image Display Values

Channel	Color	Minimum	Maximum	K
Chemi	Gray Scale (Black on White)	0.0000126	0.000198	0

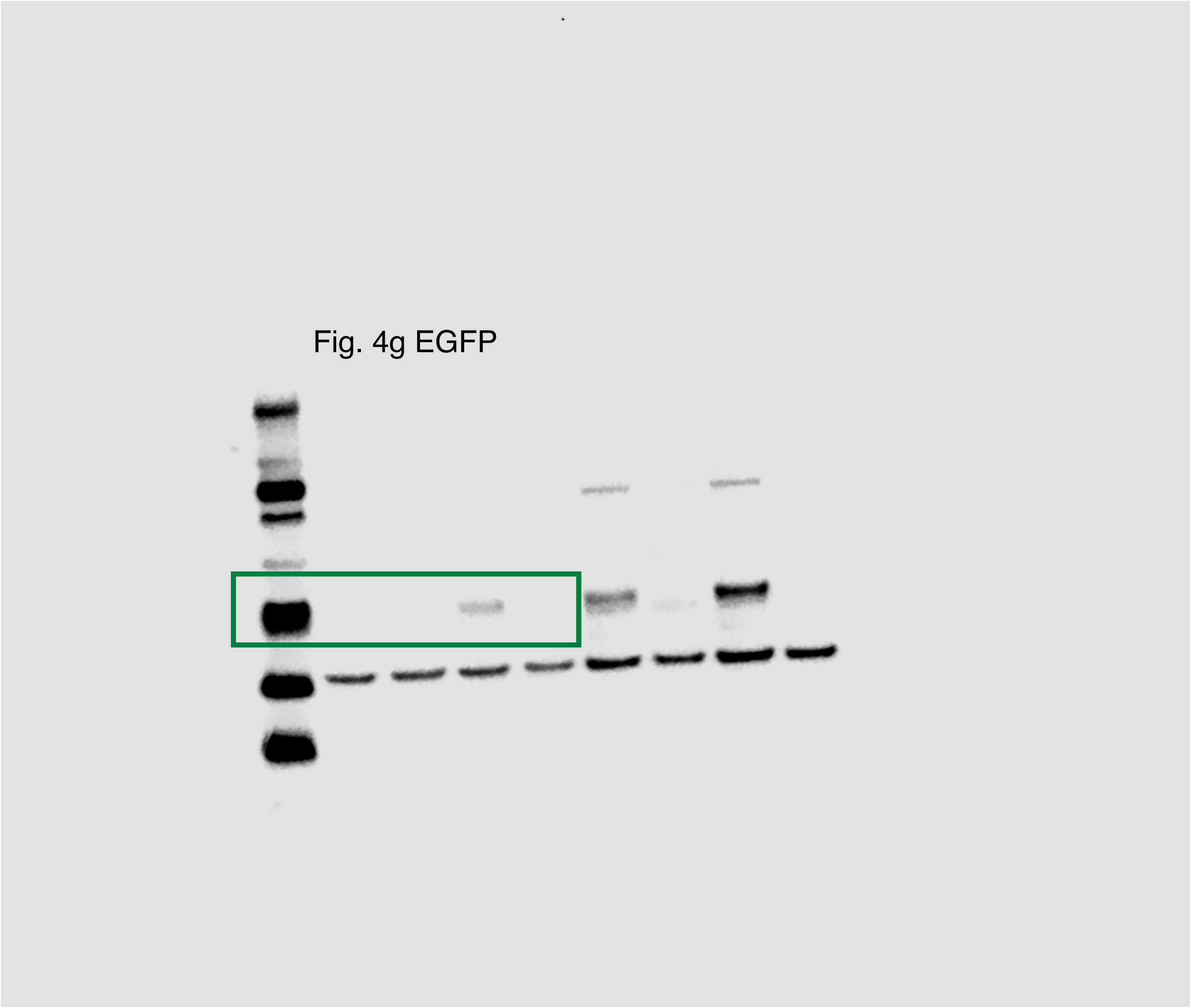

Image ID: 0005592_02
Acquire Time: Jun 26, 2025 2:15:47 PM

Acquisition Information

#	Image ID	Acquire Time	Channels	Integration Times	Analysis	Image Name	Comment
1	0005592_02	Jun 26, 2025 2:15:47 PM	Chemi	02:00	Manual	0005592_02	AID BMCs

Image Display Values

Channel	Color	Minimum	Maximum	K
Chemi	Gray Scale (Black on White)	0.0000460	0.00206	0

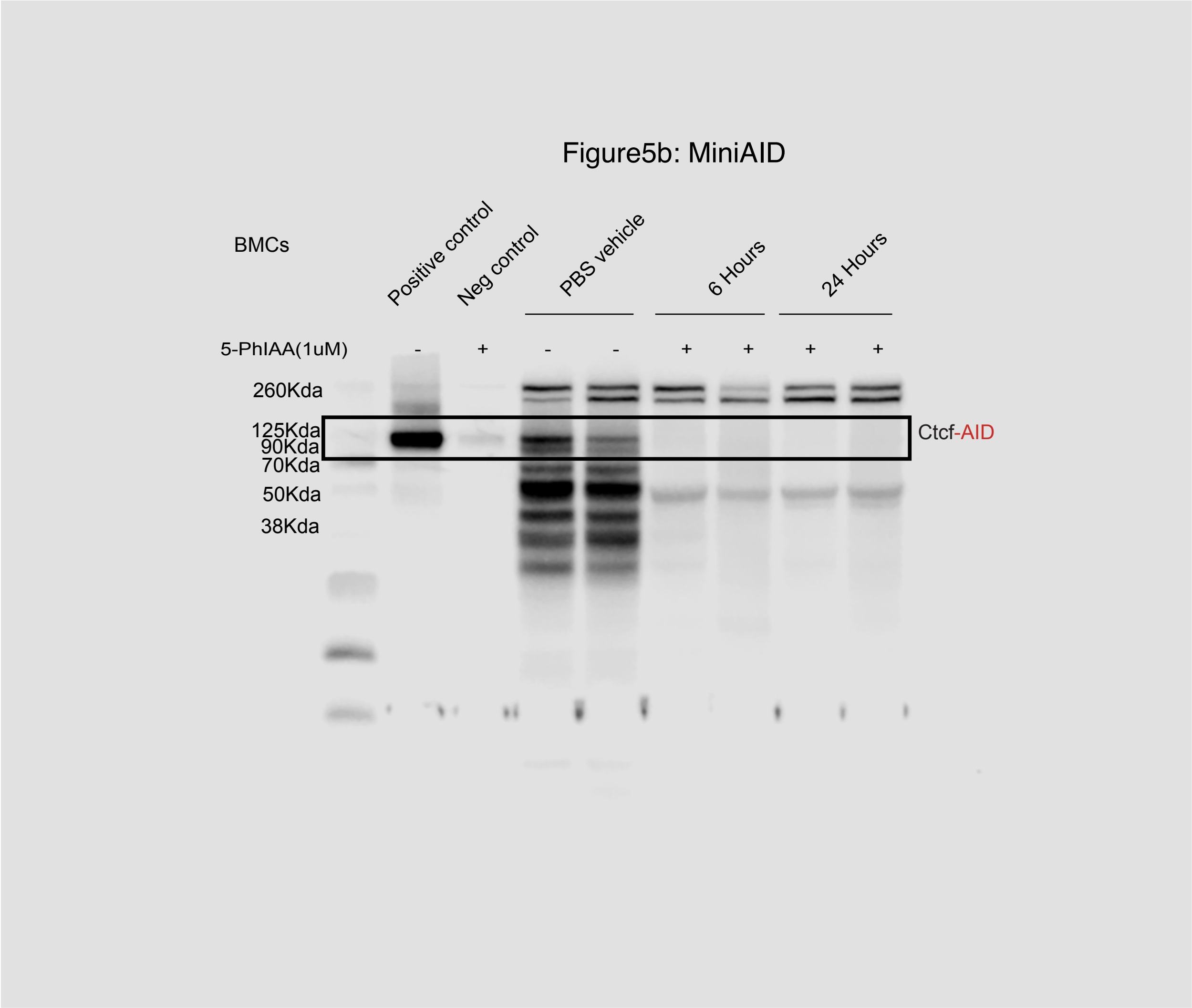

LI-COR

Image ID: 0005600_02
Acquire Time: Jun 27, 2025 11:58:04 AM

Acquisition Information

#	Image ID	Acquire Time	Channels	Integration Times	Analysis	Image Name	Comment
1	0005600_02	Jun 27, 2025 11:58:04 AM	Chemi	02:00	Manual	0005600_02	GFP for AID BMCs

Image Display Values

Channel	Color	Minimum	Maximum	K
Chemi	Gray Scale (Black on White)	0.00000101	0.00695	0

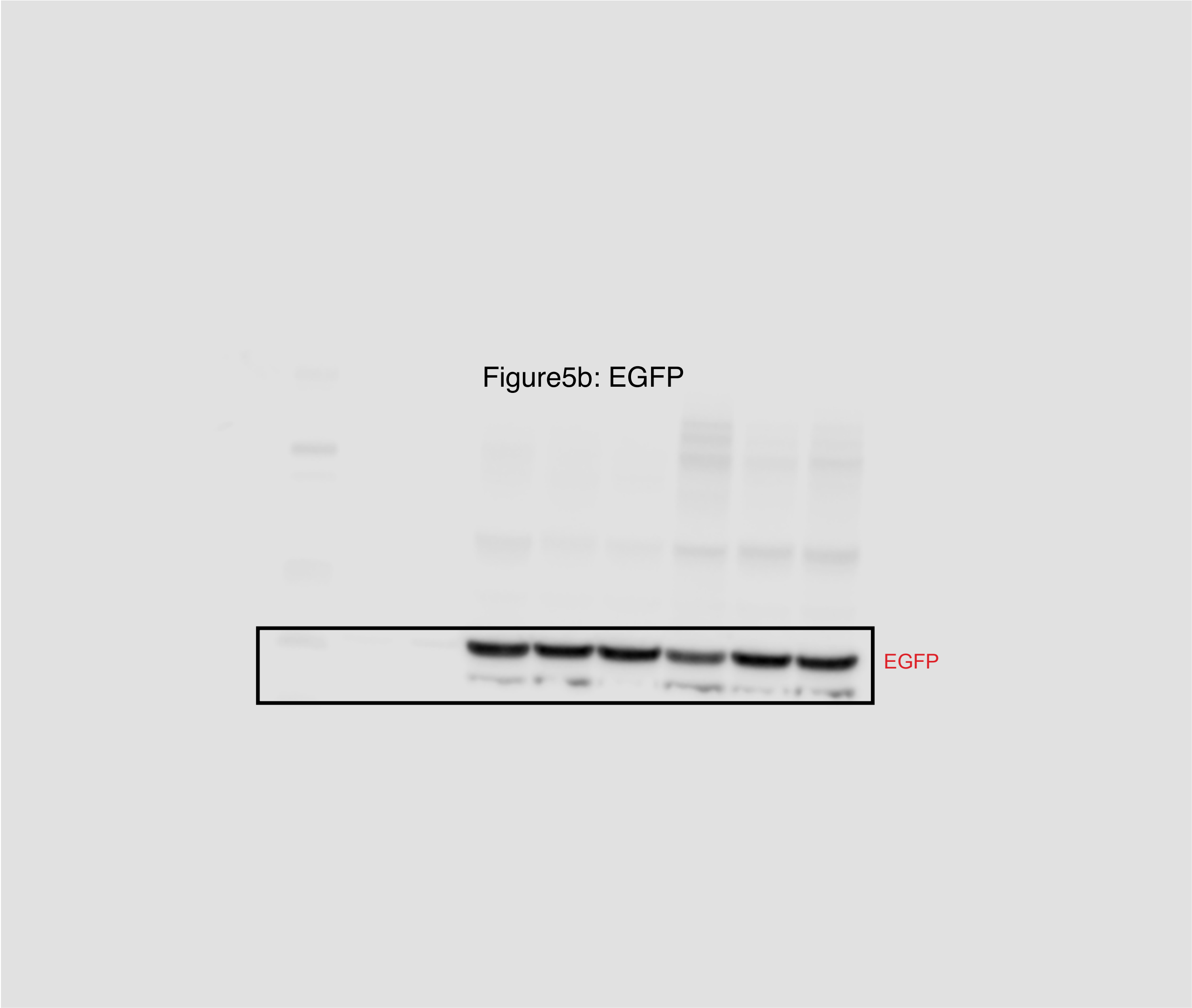

Image ID: 0005640_02
Acquire Time: Jul 2, 2025 11:43:19 AM

Acquisition Information

#	Image ID	Acquire Time	Channels	Integration Times	Analysis	Image Name
1	0005640_02	Jul 2, 2025 11:43:19 AM	Chemi	02:00	Manual	0005640_02

Image Display Values

Channel	Color	Minimum	Maximum	K
Chemi	Gray Scale (Black on White)	0.0000573	0.00312	0

Figure5c: MiniAID

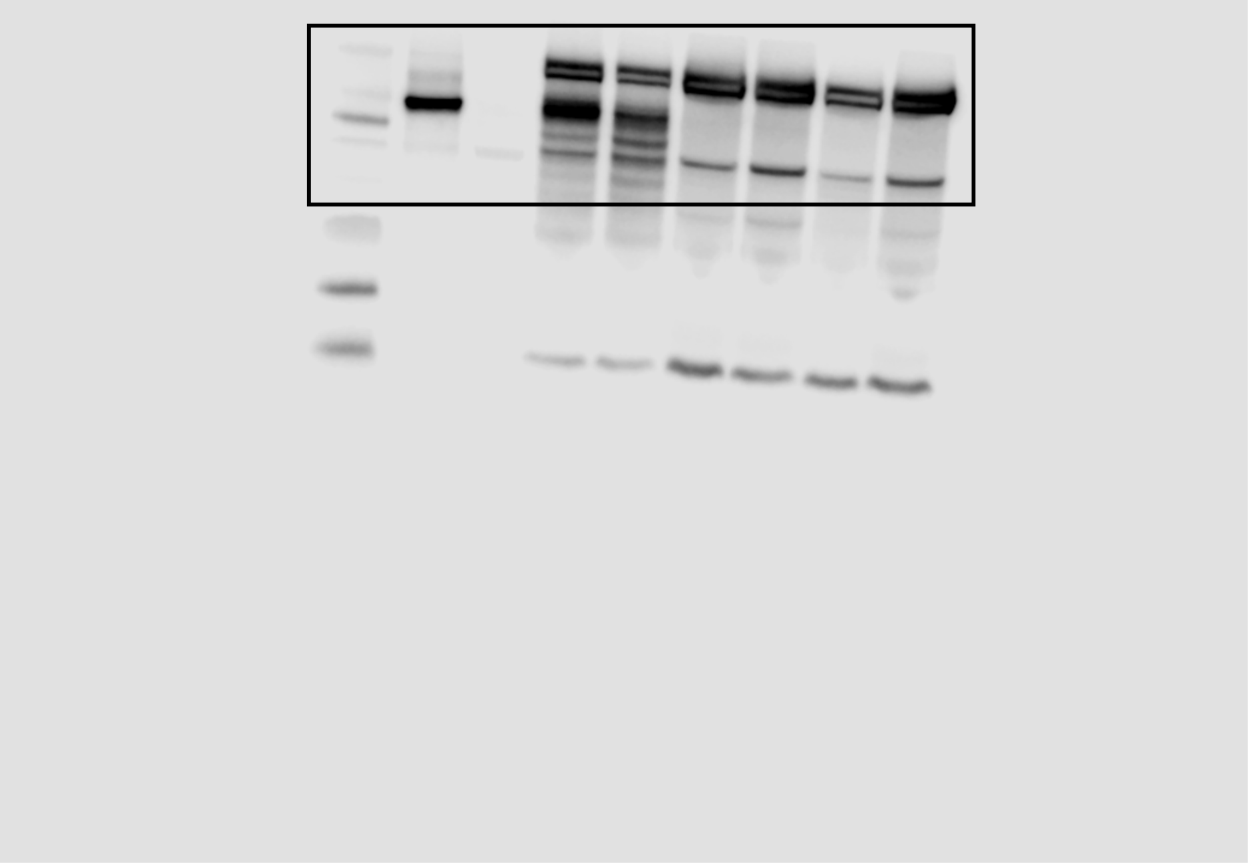

Image ID: 0005646_02
Acquire Time: Jul 2, 2025 3:15:45 PM

Acquisition Information

#	Image ID	Acquire Time	Channels	Integration Times	Analysis	Image Name	Comment
1	0005646_02	Jul 2, 2025 3:15:45 PM	Chemi	02:00	Manual	0005646_02	EGFP for AID

Image Display Values

Channel	Color	Minimum	Maximum	K
Chemi	Gray Scale (Black on White)	0.0000511	0.000404	0

Figure5c: EGFP

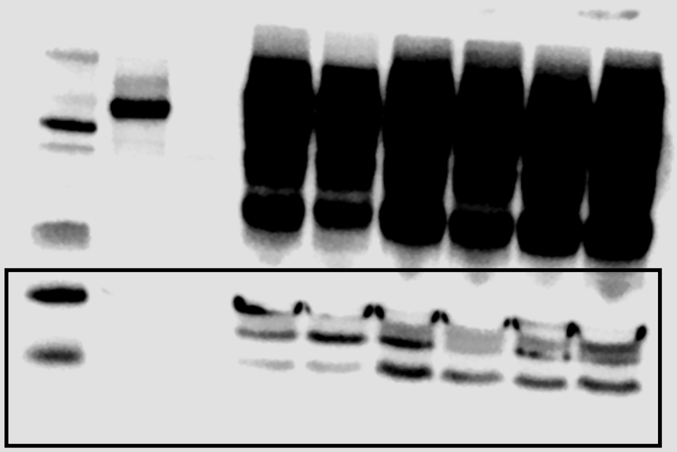

Image ID: 0005829_02
Acquire Time: Aug 13, 2025 12:20:00 PM

Acquisition Information

#	Image ID	Acquire Time	Channels	Integration Times	Analysis	Image Name	Comment
1	0005829_02	Aug 13, 2025 12:20:00 PM	Chemi	02:00	Manual	0005829_02	AID in vehicle and treatment groups

Image Display Values

Channel	Color	Minimum	Maximum	K
Chemi	Gray Scale (Black on White)	0.0000417	0.00293	0

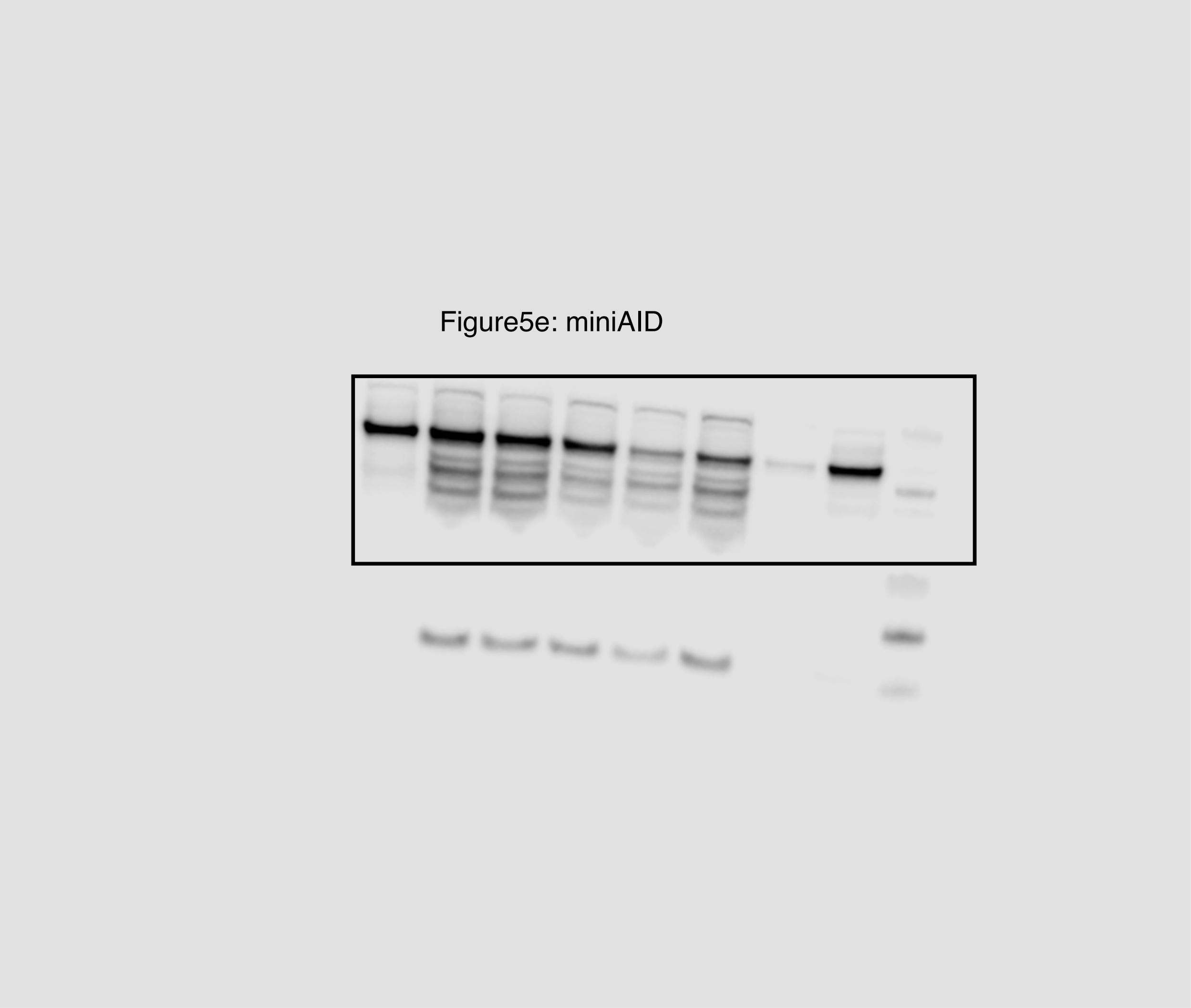

Image ID: 0005836_02
Acquire Time: Aug 13, 2025 3:52:06 PM

Acquisition Information

#	Image ID	Acquire Time	Channels	Integration Times	Analysis	Image Name
1	0005836_02	Aug 13, 2025 3:52:06 PM	Chemi	02:00	Manual	0005836_02

Image Display Values

Channel	Color	Minimum	Maximum	K
Chemi	Gray Scale (Black on White)	0.0000560	0.00463	0

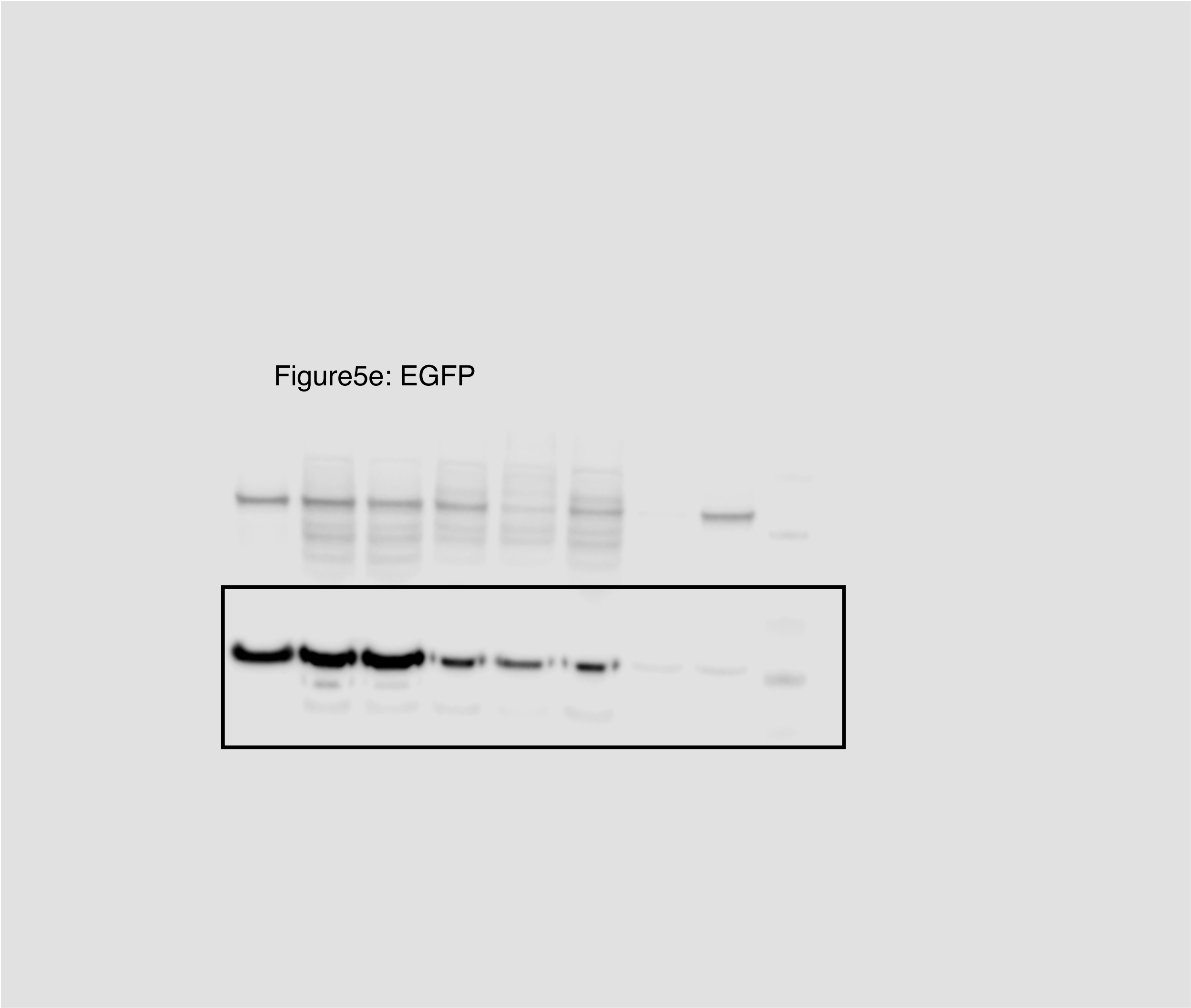

Image ID: 0005863_02
Acquire Time: Aug 20, 2025 11:26:46 AM

Acquisition Information

#	Image ID	Acquire Time	Channels	Integration Times	Analysis	Image Name	Comment
1	0005863_02	Aug 20, 2025 11:26:46 AM	Chemi	02:00	Manual	0005863_02	AID in PBS group 6h and 24hour

Image Display Values

Channel	Color	Minimum	Maximum	K
Chemi	Gray Scale (Black on White)	0.00000131	0.00732	0

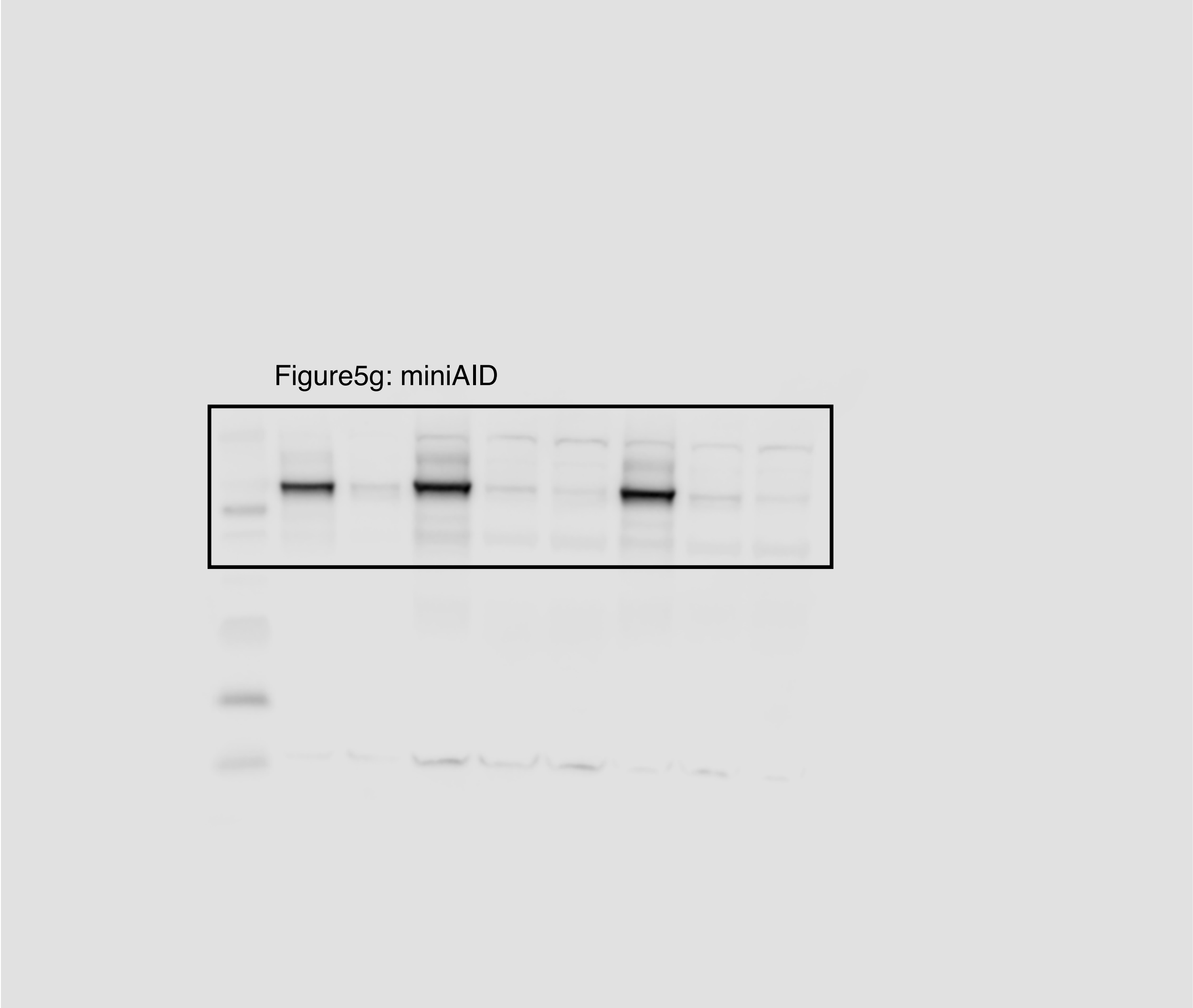

Image ID: 0005865_02
Acquire Time: Aug 20, 2025 2:58:27 PM

Acquisition Information

#	Image ID	Acquire Time	Channels	Integration Times	Analysis	Image Name	Comment
1	0005865_02	Aug 20, 2025 2:58:27 PM	Chemi	02:00	Manual	0005865_02	EGFP for AID in PBS group

Image Display Values

Channel	Color	Minimum	Maximum	K
Chemi	Gray Scale (Black on White)	0.00000113	0.0104	0

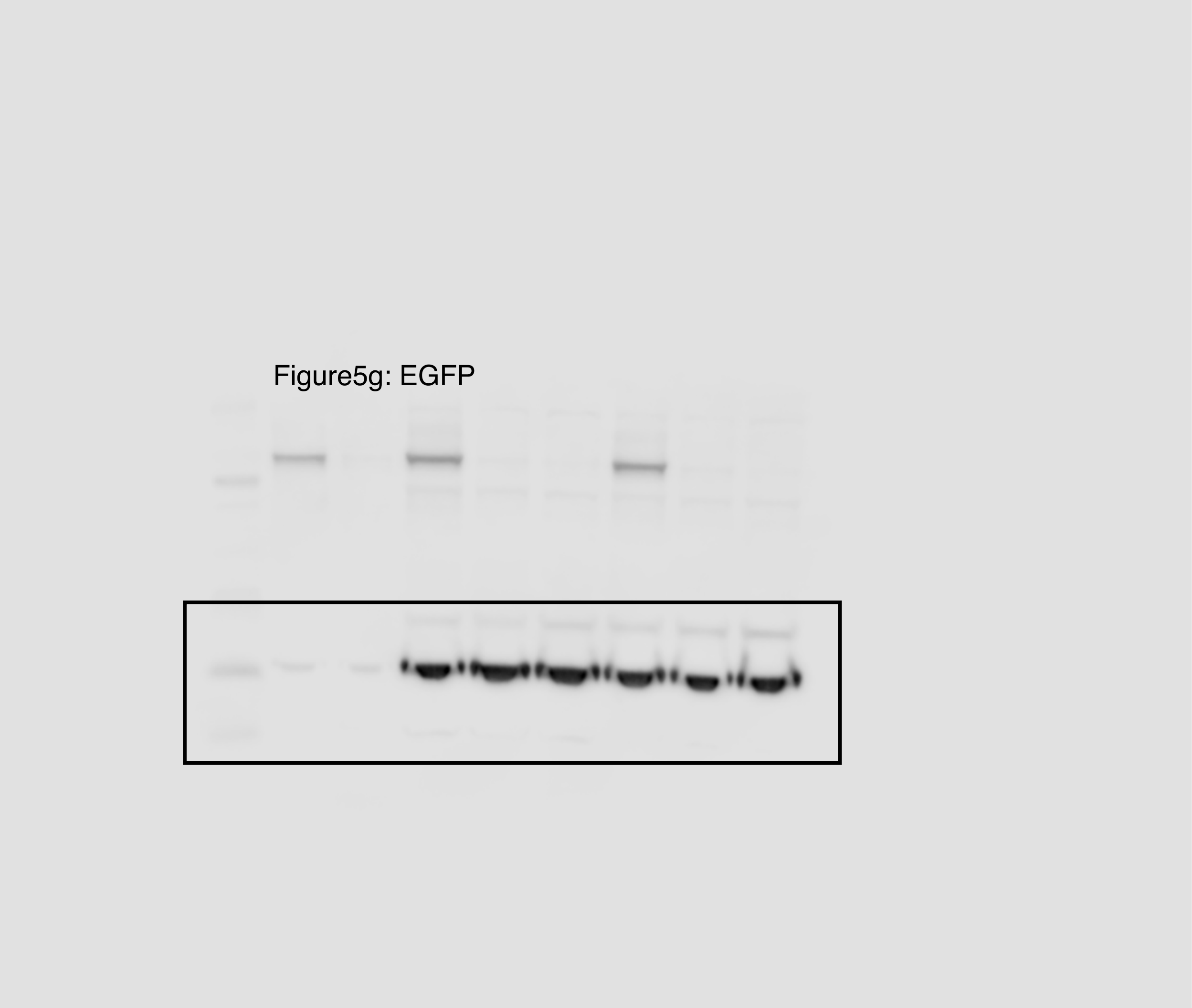

Image ID: 0005828_02
Acquire Time: Aug 13, 2025 12:15:13 PM

Acquisition Information

#	Image ID	Acquire Time	Channels	Integration Times	Analysis	Image Name	Comment
1	0005828_02	Aug 13, 2025 12:15:13 PM	Chemi	02:00	Manual	0005828_02	aid 104,105 6H 24HOUR

Image Display Values

Channel	Color	Minimum	Maximum	K
Chemi	Gray Scale (Black on White)	0.0000592	0.00380	0

Figure5h: miniAID

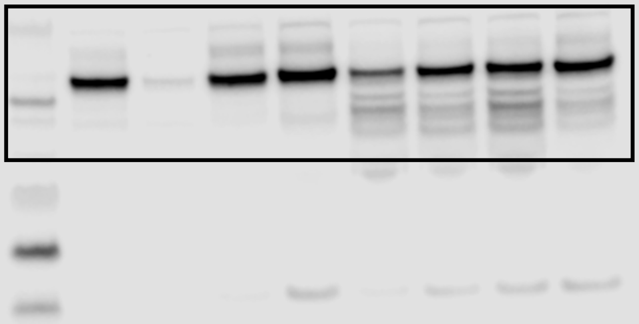

Image ID: 0005835_02
Acquire Time: Aug 13, 2025 3:47:57 PM

Acquisition Information

#	Image ID	Acquire Time	Channels	Integration Times	Analysis	Image Name	Comment
1	0005835_02	Aug 13, 2025 3:47:57 PM	Chemi	02:00	Manual	0005835_02	AID 104, 105 EGFP

Image Display Values

Channel	Color	Minimum	Maximum	K
Chemi	Gray Scale (Black on White)	0.00000113	0.00789	0

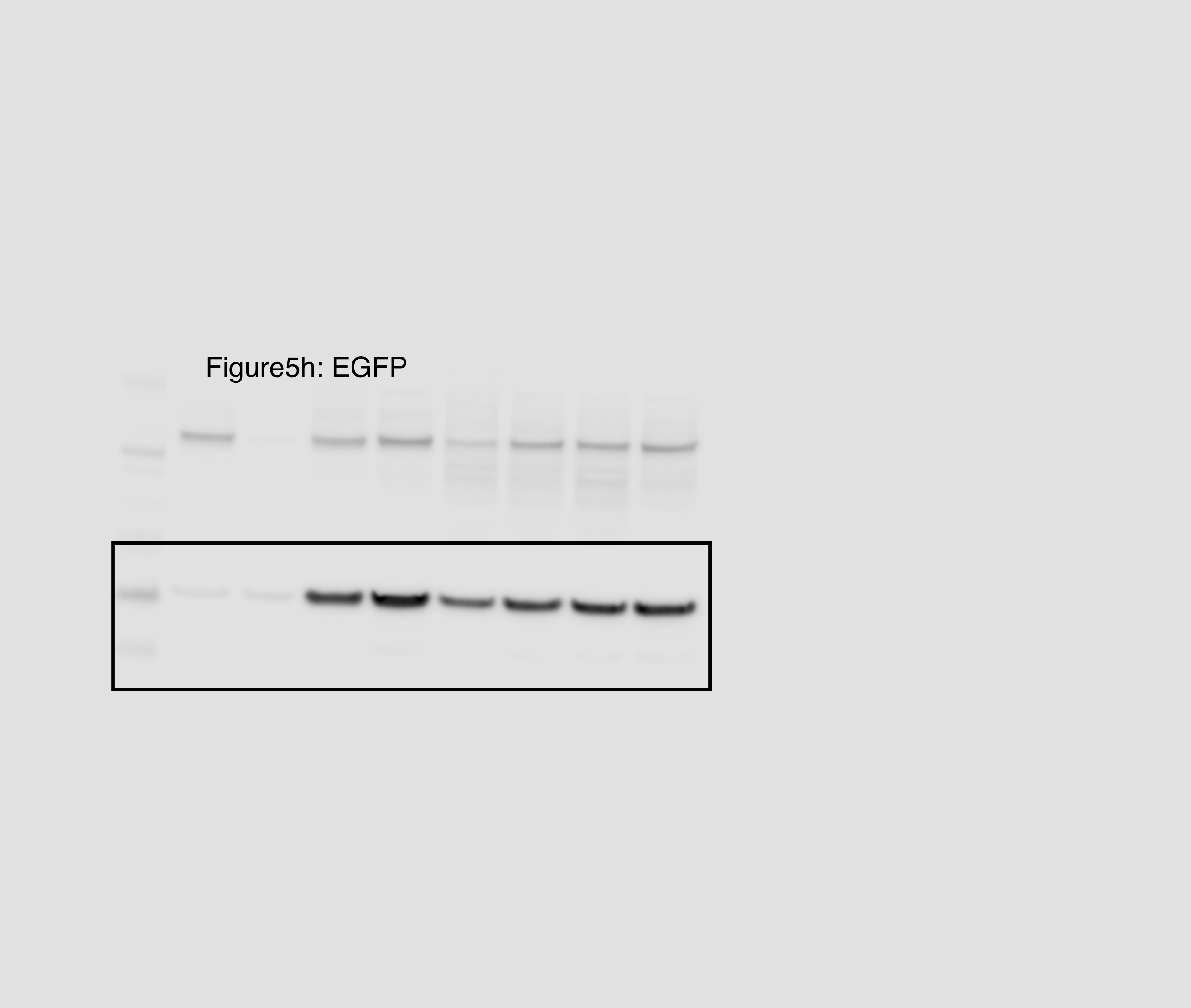

Image ID: 0006074_02
Acquire Time: Oct 28, 2025 3:18:13 PM

Acquisition Information

#	Image ID	Acquire Time	Channels	Integration Times	Analysis	Image Name	Comment
1	0006074_02	Oct 28, 2025 3:18:13 PM	Chemi	02:00	Manual	0006074_02	HSC for SP T to A

Image Display Values

Channel	Color	Minimum	Maximum	K
Chemi	Gray Scale (Black on White)	0.00000131	0.00265	0

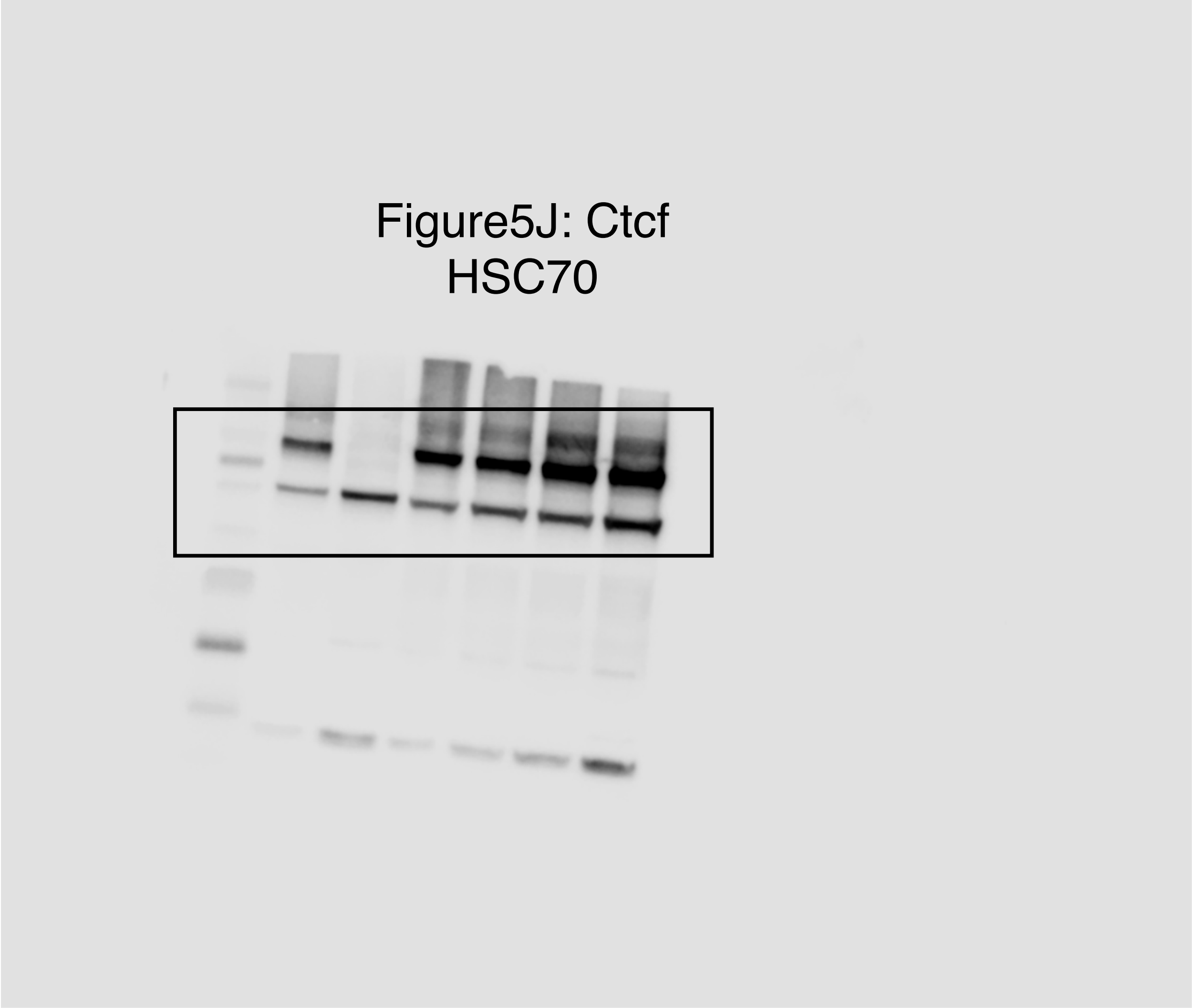

Image ID: 0000894_01
Acquire Time: Mar 31, 2022 10:08:56 AM

Acquisition Information

#	Image ID	Acquire Time	Channels	Integration Times	Analysis	Image Name	Comment	Image Modifications
1	0000894_01	Mar 31, 2022 10:08:56 AM	Chemi	04:50	Western	0000894_01		

Image Display Values

Channel	Color	Minimum	Maximum	K
Chemi	Gray Scale (Black on White)	0.0000000596	0.000139	0.09

Supplementary Fig. 1b WB CTCF

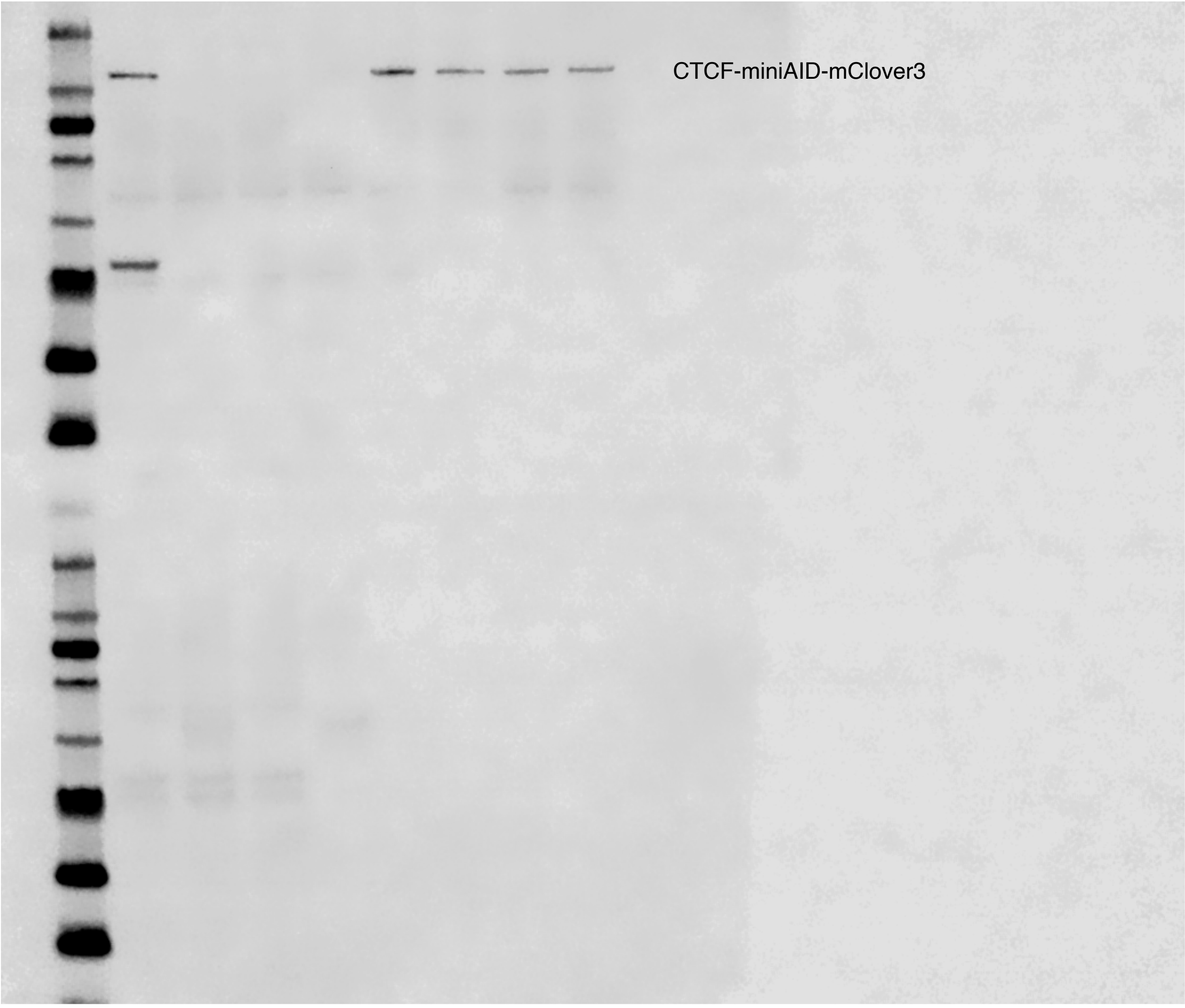

Acquisition Information

#	Image ID	Acquire Time	Channels	Integration Times	Analysis	Image Name	Comment	Image Modifications
1	0000898_01	Mar 31, 2022 12:58:12 PM	Chemi	00:35	Western	0000898_01		

Image Display Values

Channel	Color	Minimum	Maximum	K
Chemi	Gray Scale (Black on White)	0.00000232	0.00108	0

Supplementary Fig. 1b WB GAPDH

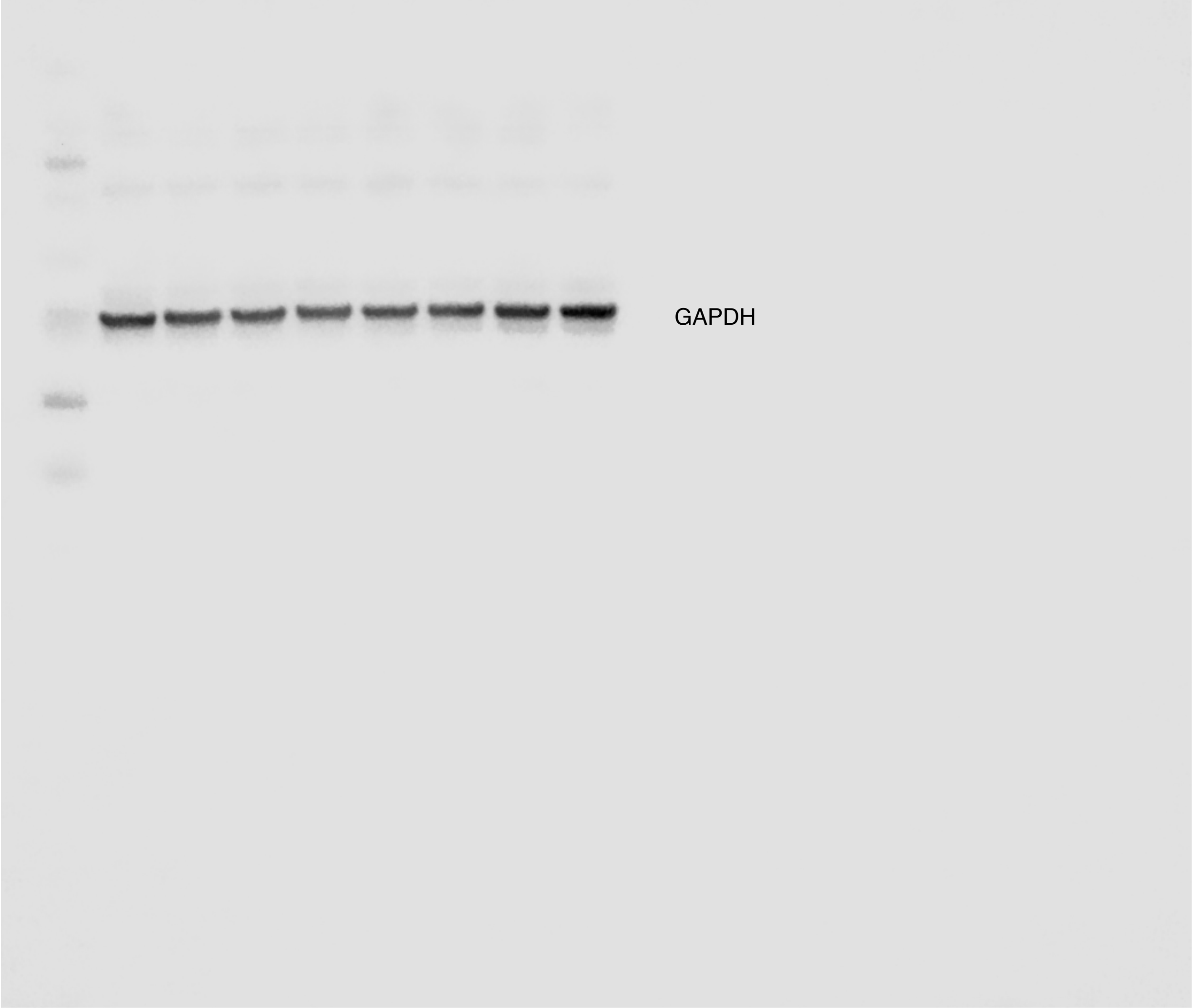

Image ID: 0003650_01
Acquire Time: Apr 10, 2024 5:39:18 PM

Acquisition Information

#	Image ID	Acquire Time	Channels	Integration Times	Analysis	Image Name	Comment	Image Modifications
1	0003650_01	Apr 10, 2024 5:39:18 PM	Chemi	04:20	Manual	0003650_01		

Image Display Values

Channel	Color	Minimum	Maximum	K
Chemi	Gray Scale (Black on White)	0.000000834	0.00234	0

Supplementary Fig. 1e WB CTCF

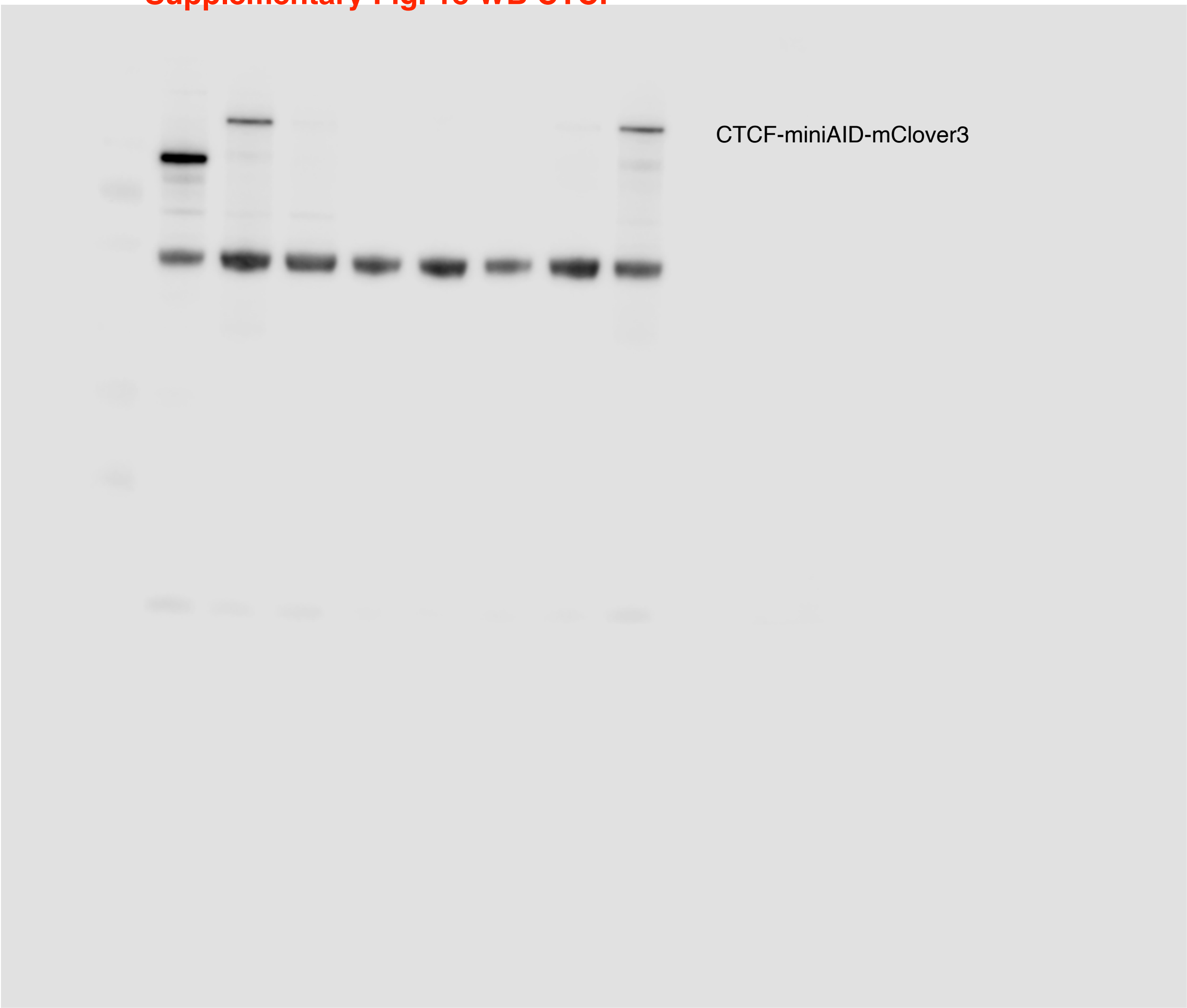

Image ID: 0003658_01
Acquire Time: Apr 11, 2024 12:11:12 PM

Acquisition Information

#	Image ID	Acquire Time	Channels	Integration Times	Analysis	Image Name	Comment	Image Modifications
1	0003658_01	Apr 11, 2024 12:11:12 PM	Chemi	04:00	Manual	0003658_01		

Image Display Values

Channel	Color	Minimum	Maximum	K
Chemi	Gray Scale (Black on White)	0.0000000596	0.000419	0.23

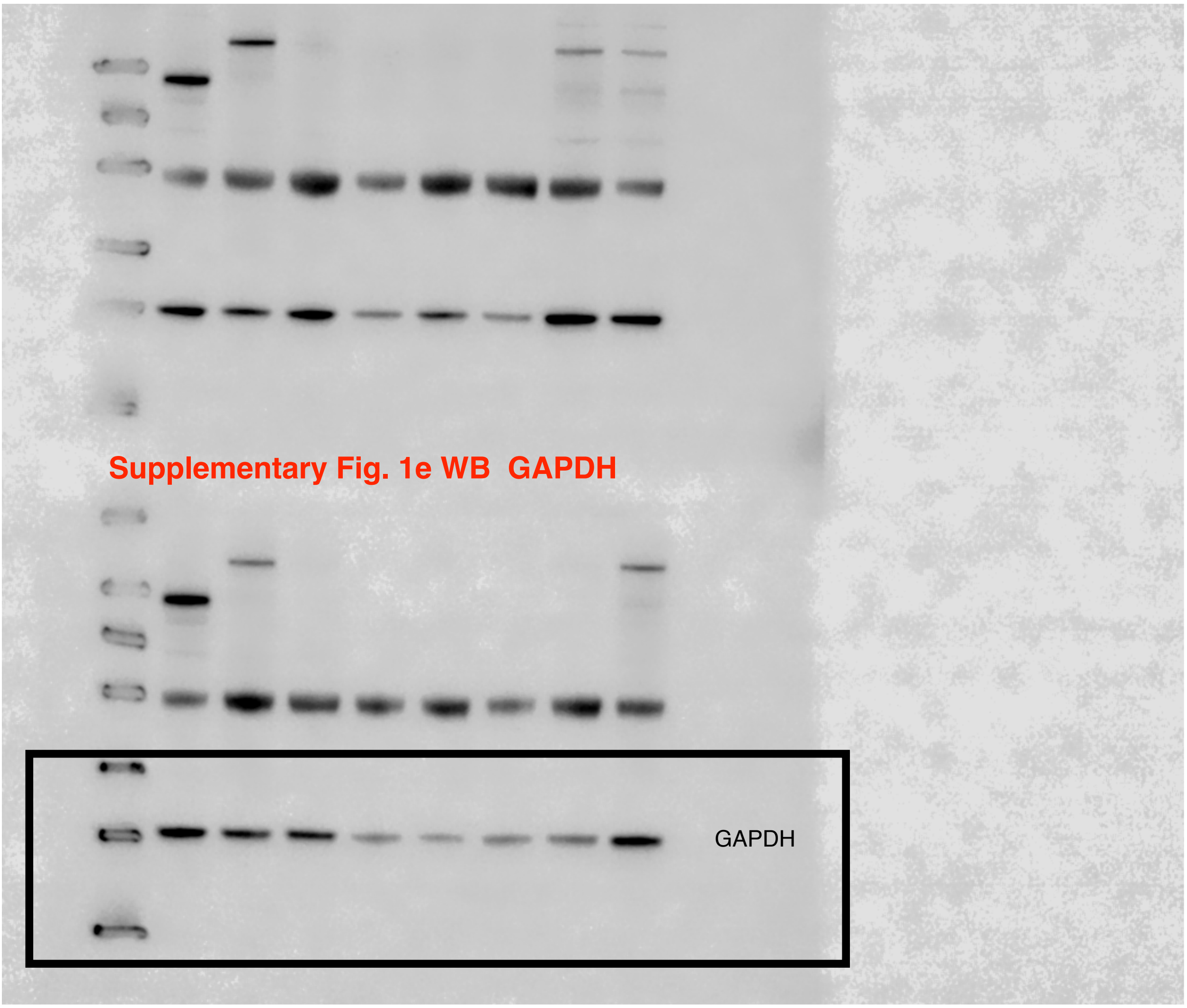

Image ID: 0003652_01
Acquire Time: Apr 10, 2024 5:50:23 PM

Acquisition Information

#	Image ID	Acquire Time	Channels	Integration Times	Analysis	Image Name	Comment	Image Modifications
1	0003652_01	Apr 10, 2024 5:50:23 PM	Chemi	04:20	Manual	0003652_01		

Image Display Values

Channel	Color	Minimum	Maximum	K
Chemi	Gray Scale (Black on White)	0.0000222	0.000755	0

Supplementary Fig. 1f WB CTCF

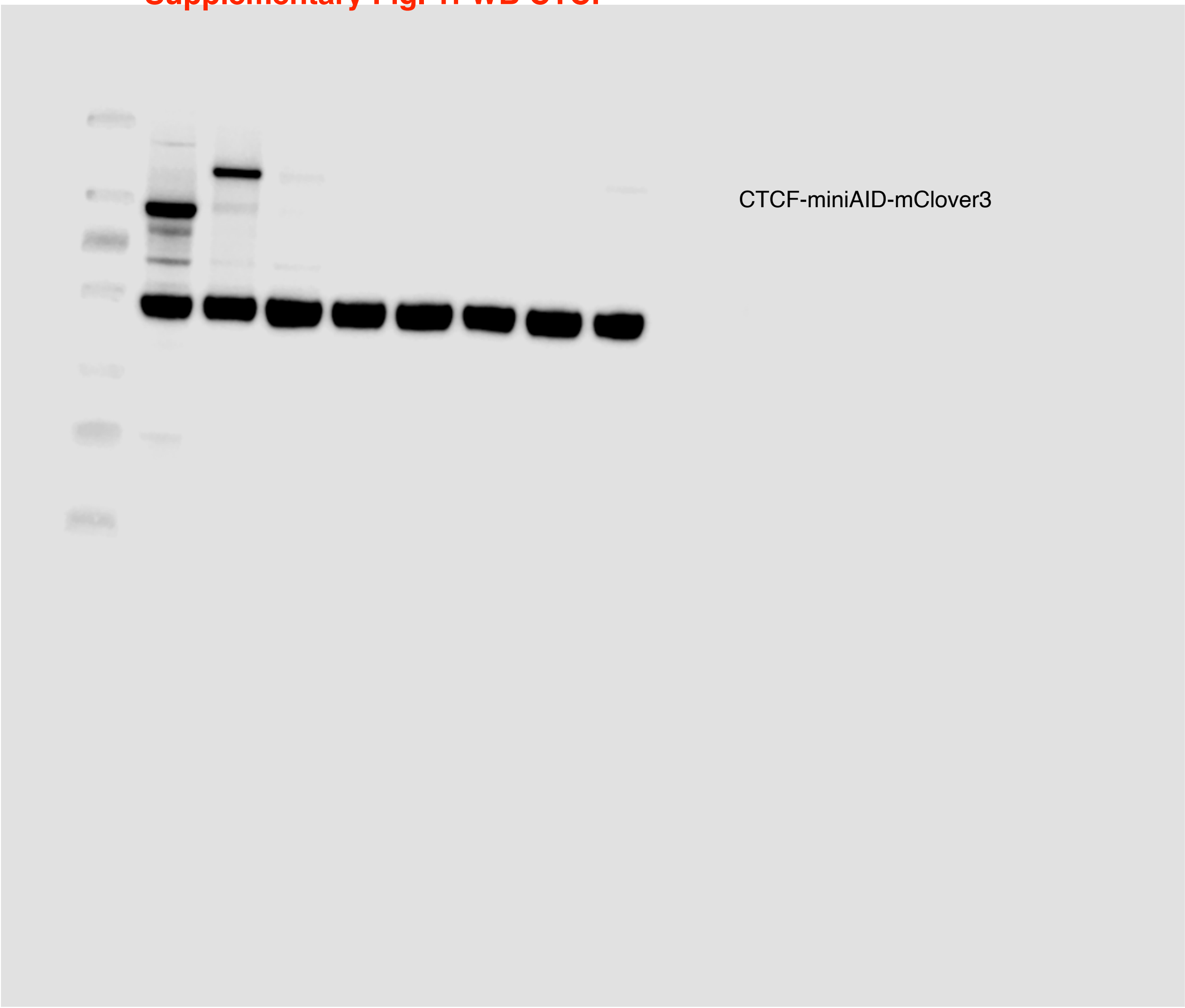

Image ID: 0003659_01
Acquire Time: Apr 11, 2024 12:19:50 PM

Acquisition Information

#	Image ID	Acquire Time	Channels	Integration Times	Analysis	Image Name	Comment	Image Modifications
1	0003659_01	Apr 11, 2024 12:19:50 PM	Chemi	02:00	Manual	0003659_01		

Image Display Values

Channel	Color	Minimum	Maximum	K
Chemi	Gray Scale (Black on White)	0.0000196	0.000881	0

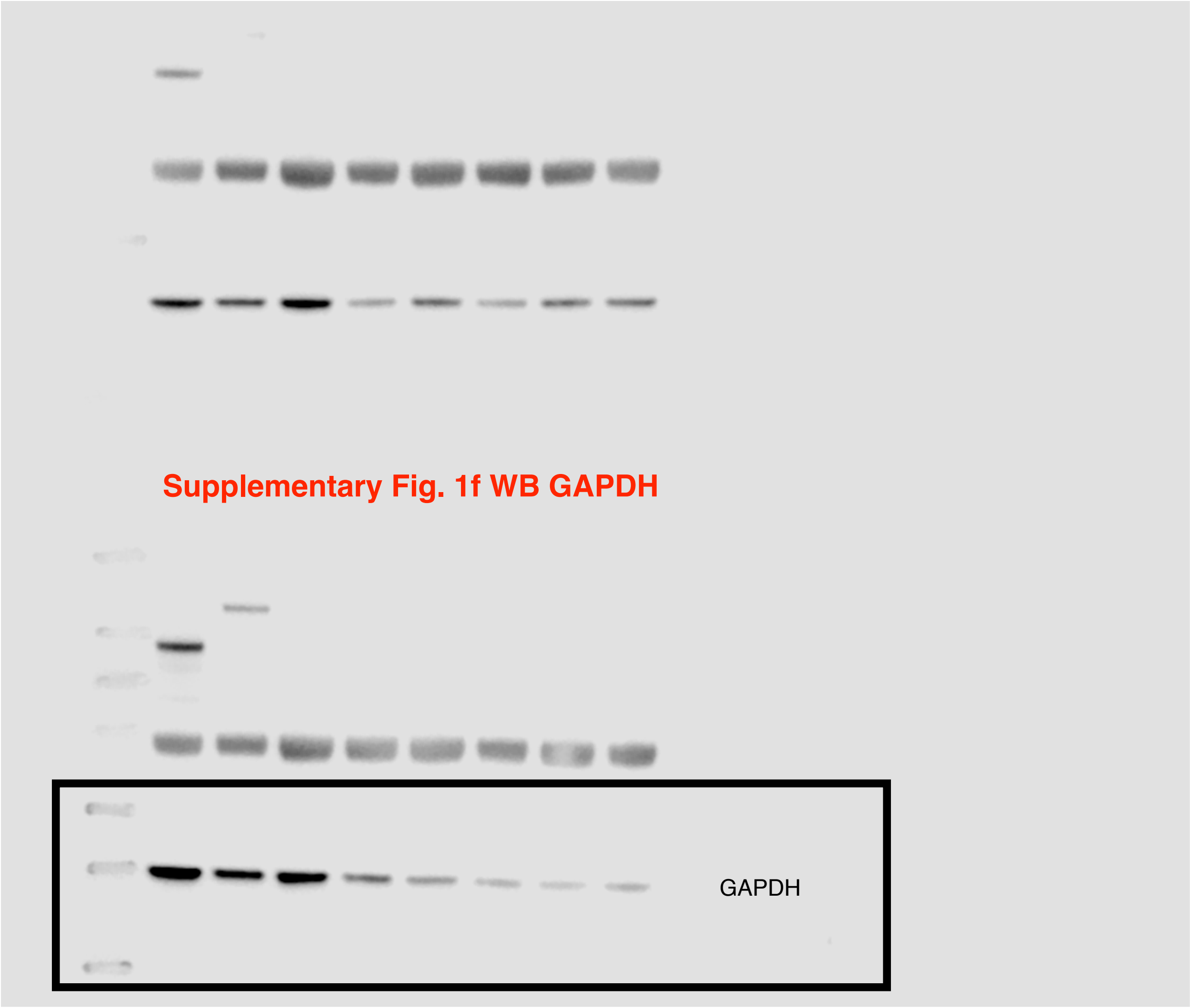

Image ID: 0003739_01
Acquire Time: Apr 19, 2024 12:11:55 PM

Acquisition Information

#	Image ID	Acquire Time	Channels	Integration Times	Analysis	Image Name	Comment	Image Modifications
1	0003739_01	Apr 19, 2024 12:11:55 PM	Chemi	04:00	Manual	0003739_01		

Image Display Values

Channel	Color	Minimum	Maximum	K
Chemi	Gray Scale (Black on White)	0.0000955	0.00438	0

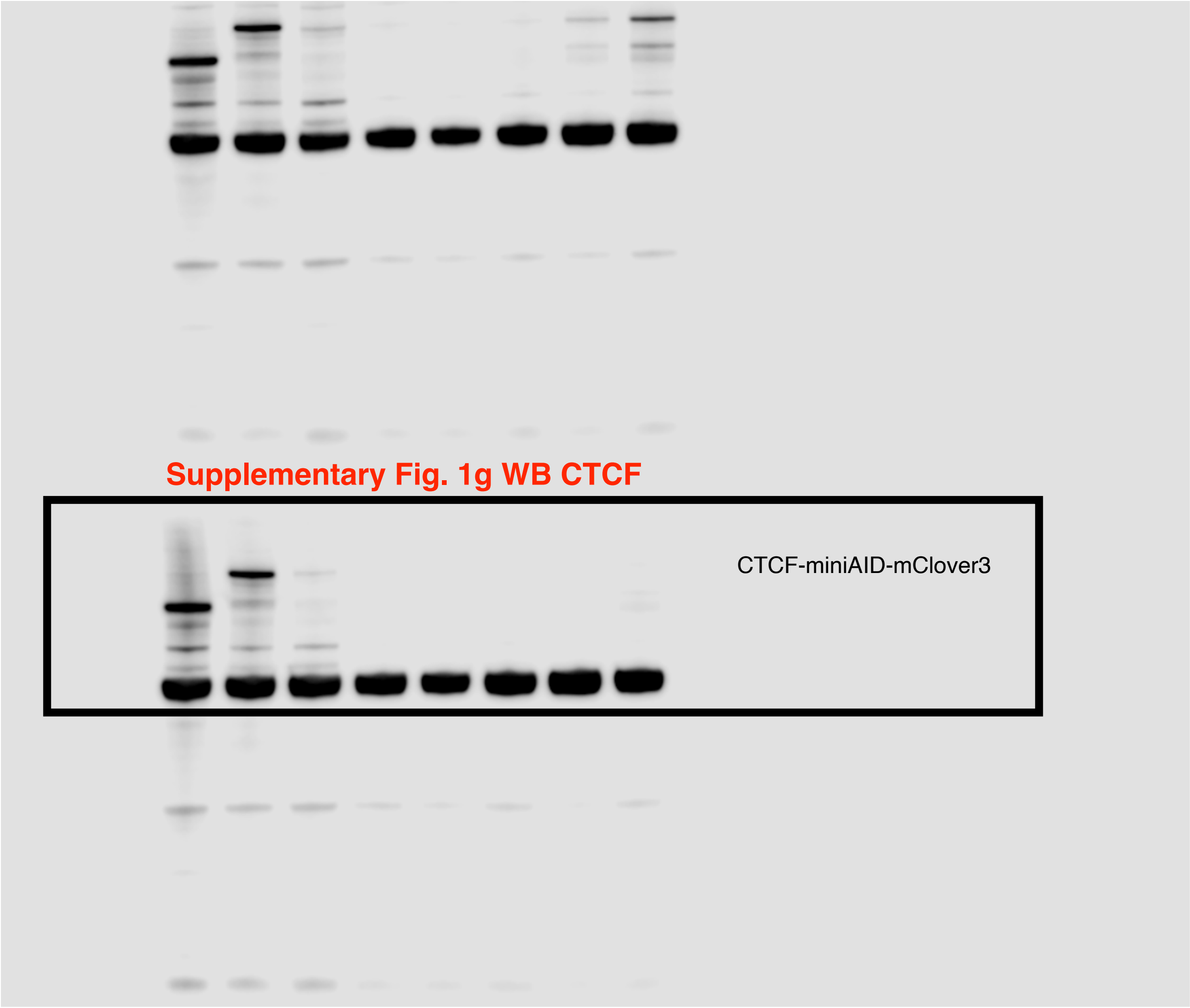

Image ID: 0003736_01
Acquire Time: Apr 18, 2024 4:37:02 PM

Acquisition Information

#	Image ID	Acquire Time	Channels	Integration Times	Analysis	Image Name	Comment	Image Modifications
1	0003736_01	Apr 18, 2024 4:37:02 PM	Chemi	02:00	Manual	0003736_01		

Image Display Values

Channel	Color	Minimum	Maximum	K
Chemi	Gray Scale (Black on White)	0.0000532	0.000954	0

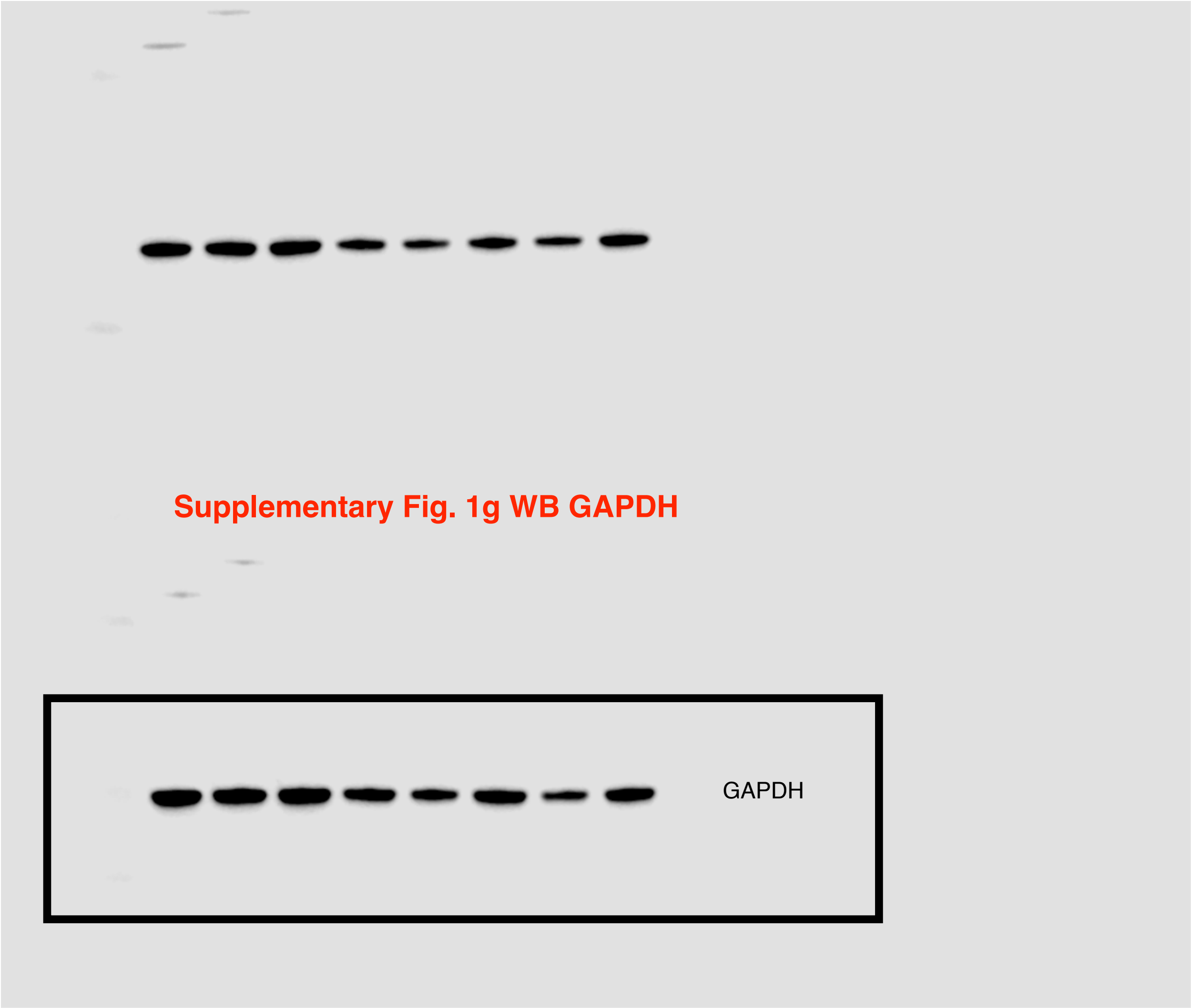

Image ID: 0003407_01
Acquire Time: Feb 15, 2024 11:13:22 AM

Acquisition Information

#	Image ID	Acquire Time	Channels	Integration Times	Analysis	Image Name	Comment	Image Modifications
1	0003407_01	Feb 15, 2024 11:13:22 AM	Chemi	02:00	Manual	0003407_01		

Image Display Values

Channel	Color	Minimum	Maximum	K
Chemi	Gray Scale (Black on White)	0.0000420	0.00418	0

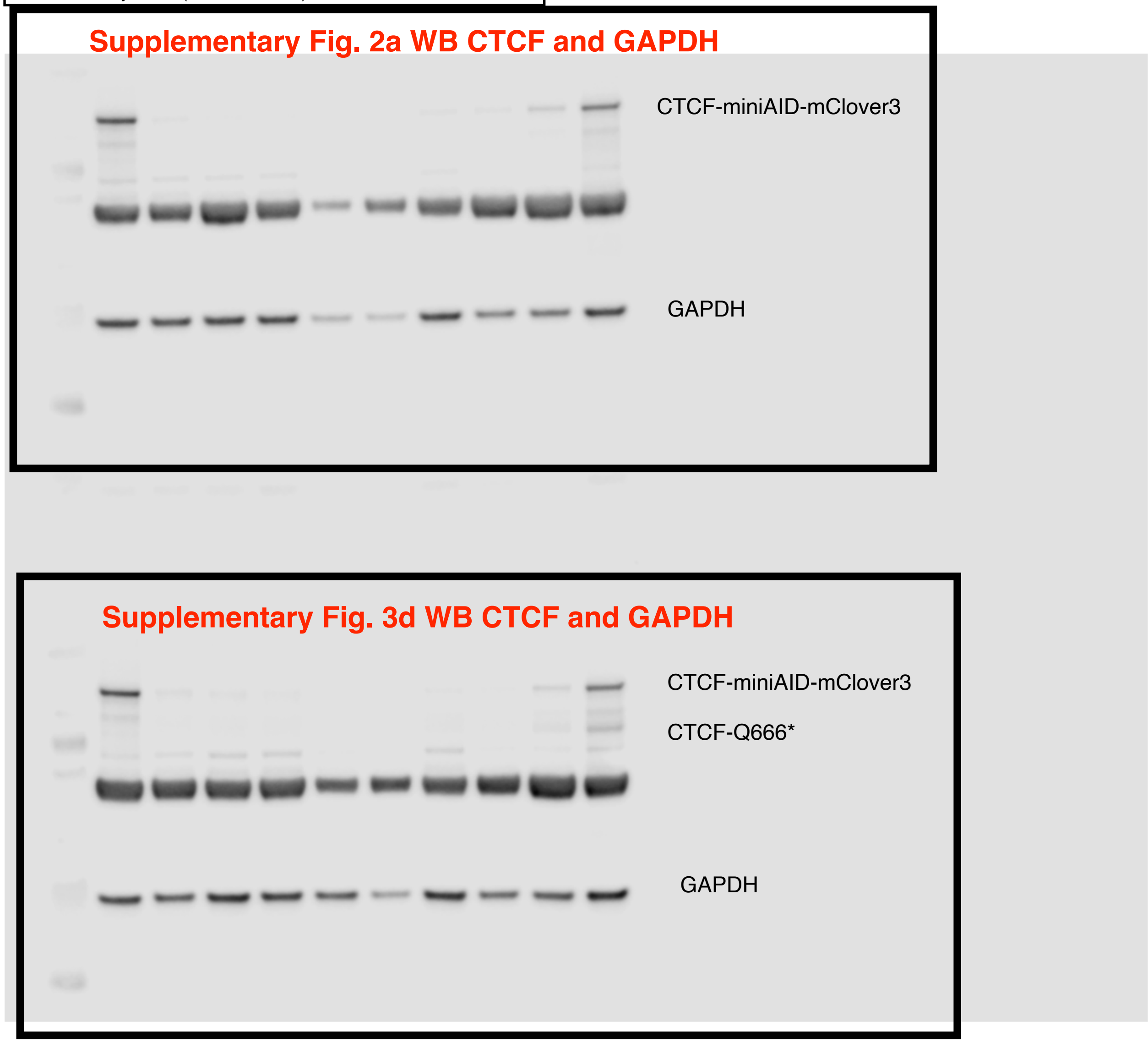

Image ID: 0003406_01
Acquire Time: Feb 15, 2024 11:07:03 AM

Acquisition Information

#	Image ID	Acquire Time	Channels	Integration Times	Analysis	Image Name	Comment	Image Modifications
1	0003406_01	Feb 15, 2024 11:07:03 AM	Chemi	04:30	Manual	0003406_01		

Image Display Values

Channel	Color	Minimum	Maximum	K
Chemi	Gray Scale (Black on White)	0.00000238	0.000319	0

Supplementary Fig. 2a WB miniAID

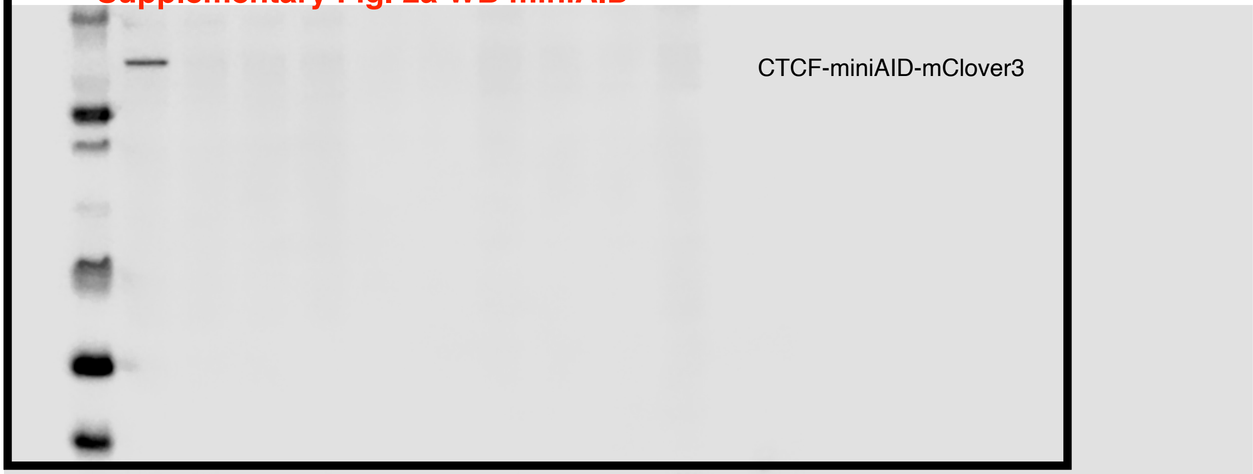

Supplementary Fig. 3d WB CTCF and GAPDH

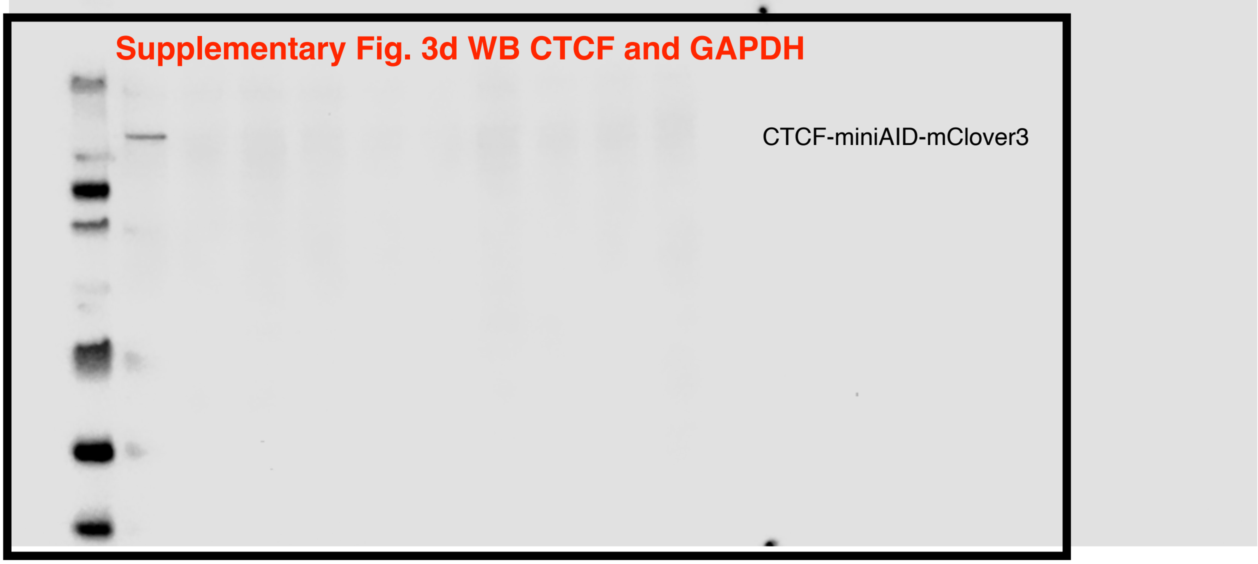

Image ID: 0004734_01
Acquire Time: Nov 21, 2024 9:08:37 AM

Acquisition Information

#	Image ID	Acquire Time	Channels	Integration Times	Analysis	Image Name	Comment	Image Modifications
1	0004734_01	Nov 21, 2024 9:08:37 AM	Chemi	01:58	Manual	0004734_01		

Image Display Values

Channel	Color	Minimum	Maximum	K
Chemi	Gray Scale (Black on White)	0.00000149	0.000266	0

Supplementary Fig. 3c WB miniAID

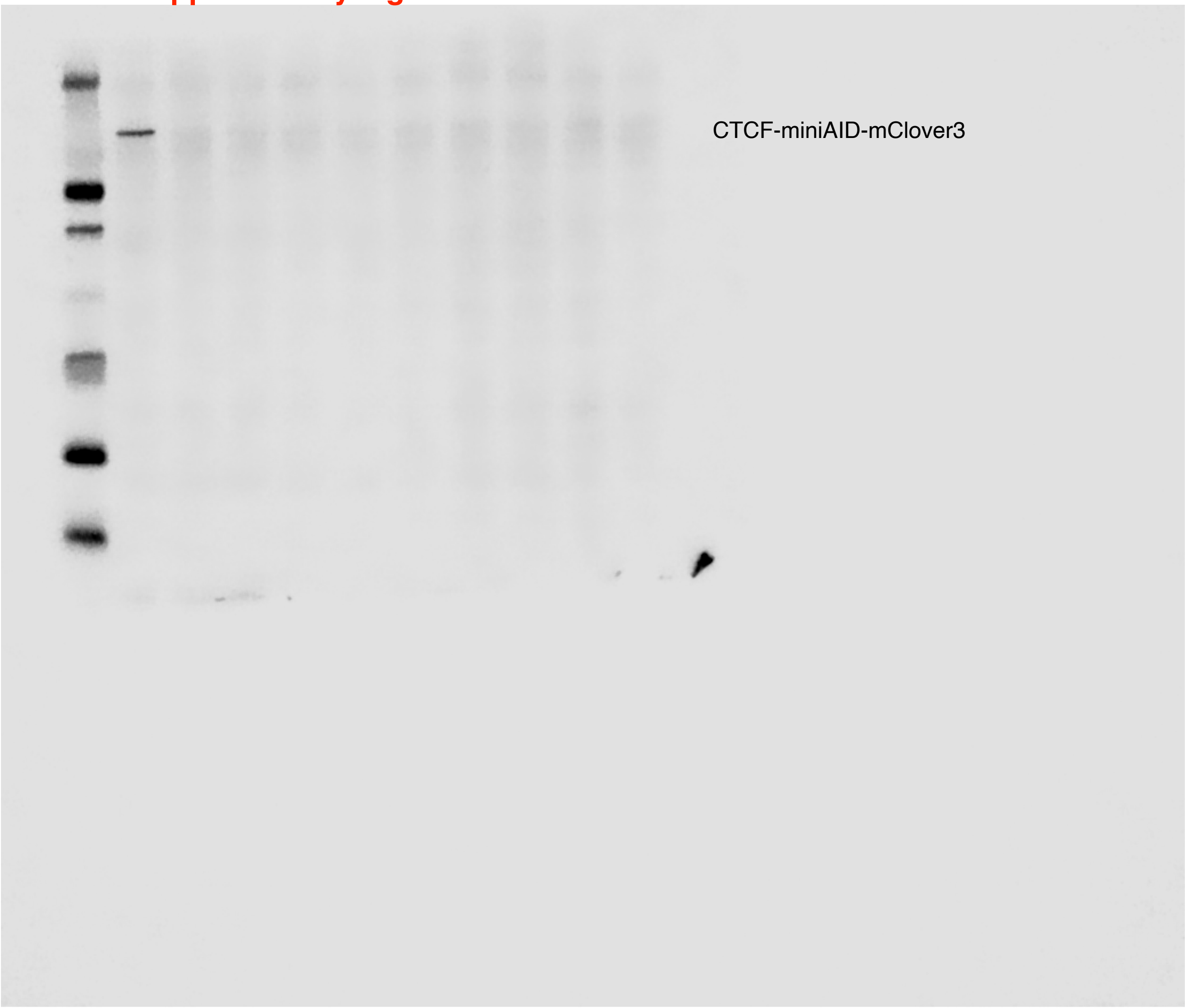

Image ID: 0004736_02
Acquire Time: Nov 21, 2024 10:09:47 AM

Acquisition Information

#	Image ID	Acquire Time	Channels	Integration Times	Analysis	Image Name	Comment
1	0004736_02	Nov 21, 2024 10:09:47 AM	Chemi	01:58	Manual	0004736_02	

Image Display Values

Channel	Color	Minimum	Maximum	K
Chemi	Gray Scale (Black on White)	0.00000489	0.00578	0

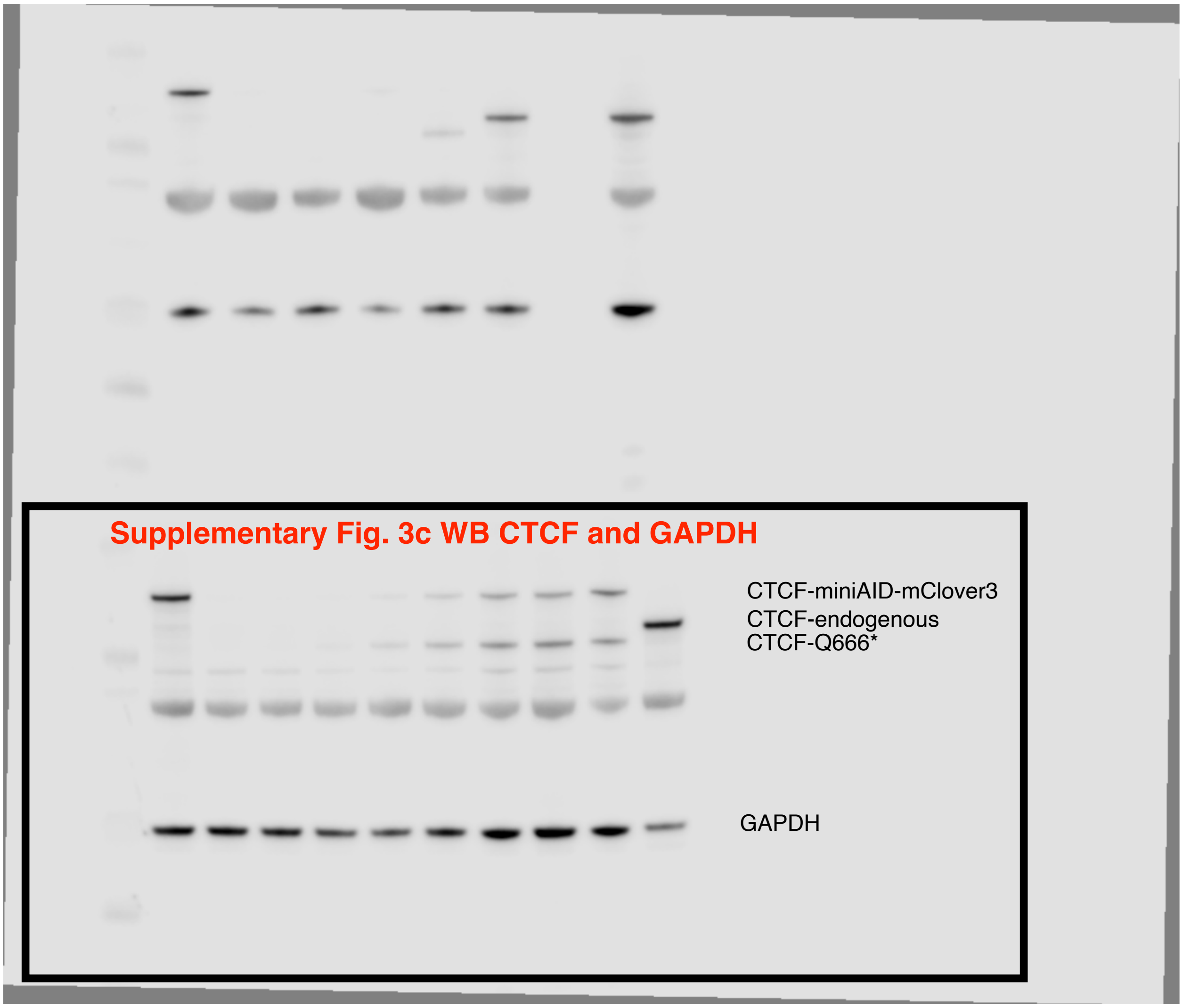

Image ID: 0005656_02
Acquire Time: Jul 8, 2025 11:00:55 AM

Acquisition Information

#	Image ID	Acquire Time	Channels	Integration Times	Analysis	Image Name	Comment
1	0005656_02	Jul 8, 2025 11:00:55 AM	Chemi	02:00	Manual	0005656_02	

Image Display Values

Channel	Color	Minimum	Maximum	K
Chemi	Gray Scale (Black on White)	0.00000364	0.00367	0.05

Supplementary Fig. 4a

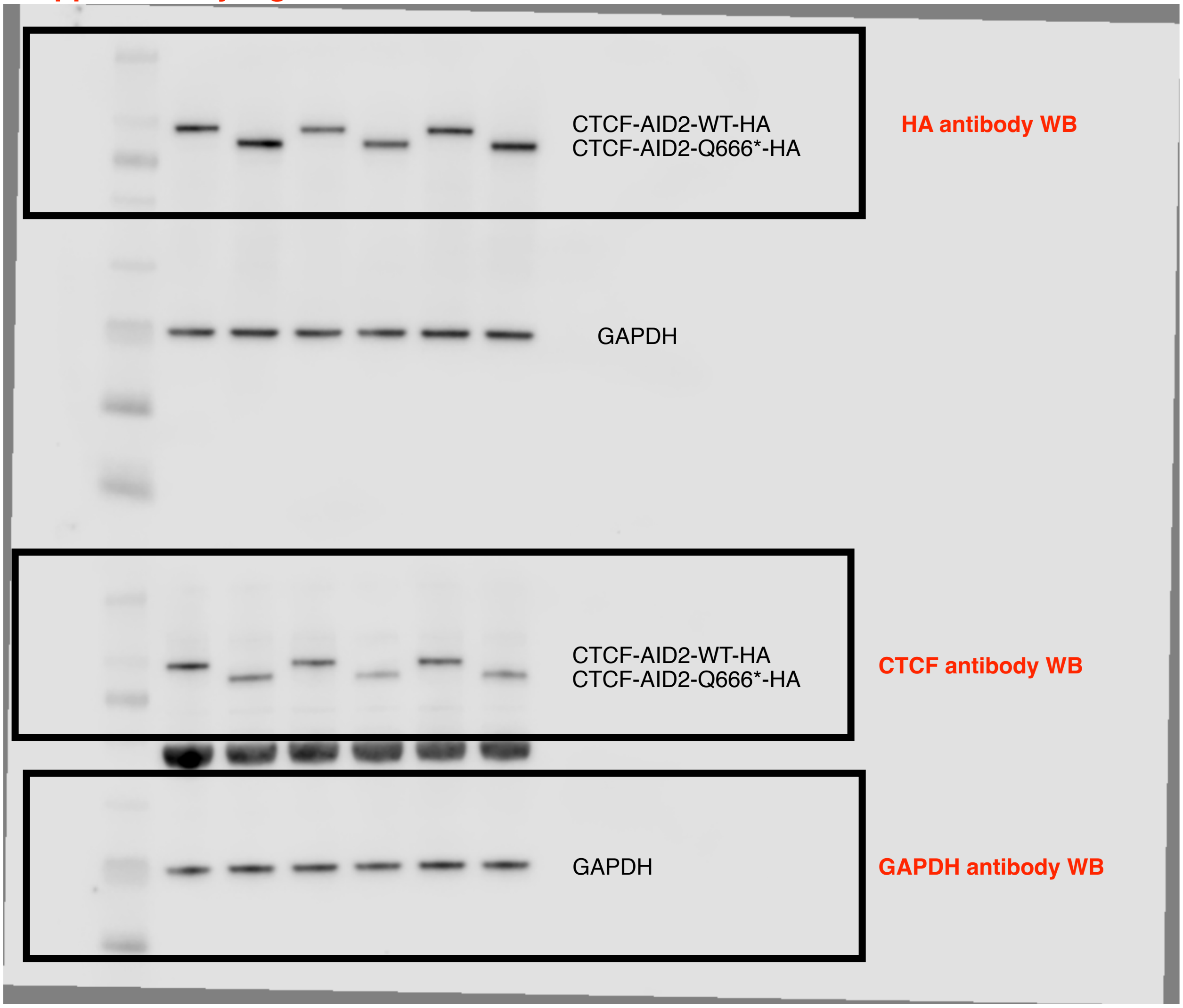

Image ID: 0004909_02
Acquire Time: Jan 8, 2025 11:05:18 AM

Acquisition Information

#	Image ID	Acquire Time	Channels	Integration Times	Analysis	Image Name	Comment
1	0004909_02	Jan 8, 2025 11:05:18 AM	Chemi	01:58	Manual	0004909_02	

Image Display Values

Channel	Color	Minimum	Maximum	K
Chemi	Gray Scale (Black on White)	0.0000167	0.000377	0

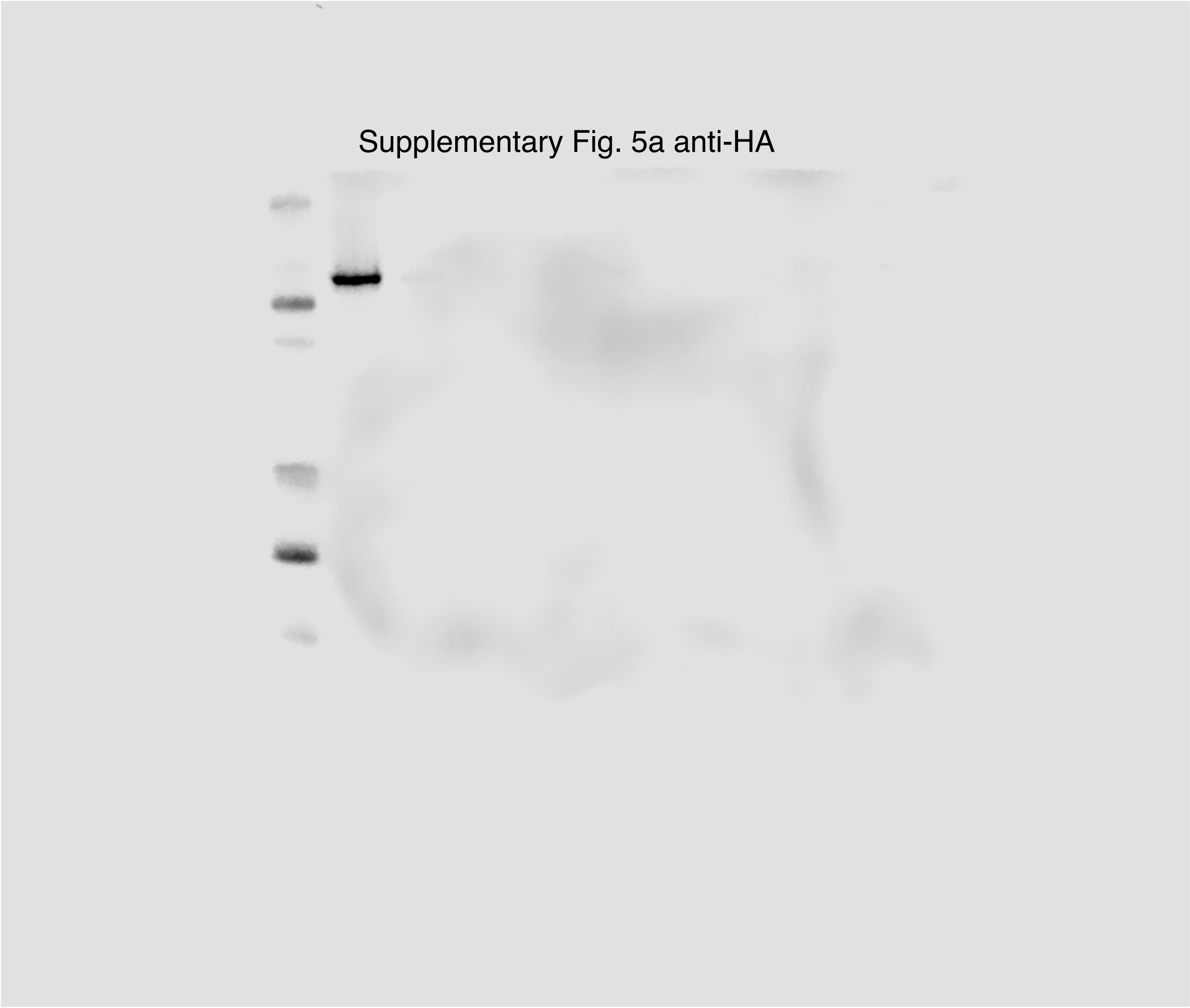

Image ID: 0004908_01
Acquire Time: Jan 8, 2025 11:01:48 AM

Acquisition Information

#	Image ID	Acquire Time	Channels	Integration Times	Analysis	Image Name	Comment	Image Modifications
1	0004908_01	Jan 8, 2025 11:01:48 AM	Chemi	01:58	Western	0004908_01		

Image Display Values

Channel	Color	Minimum	Maximum	K
Chemi	Gray Scale (Black on White)	0.00000352	0.000152	0

Supplementary Fig. 5a anti-RBM5

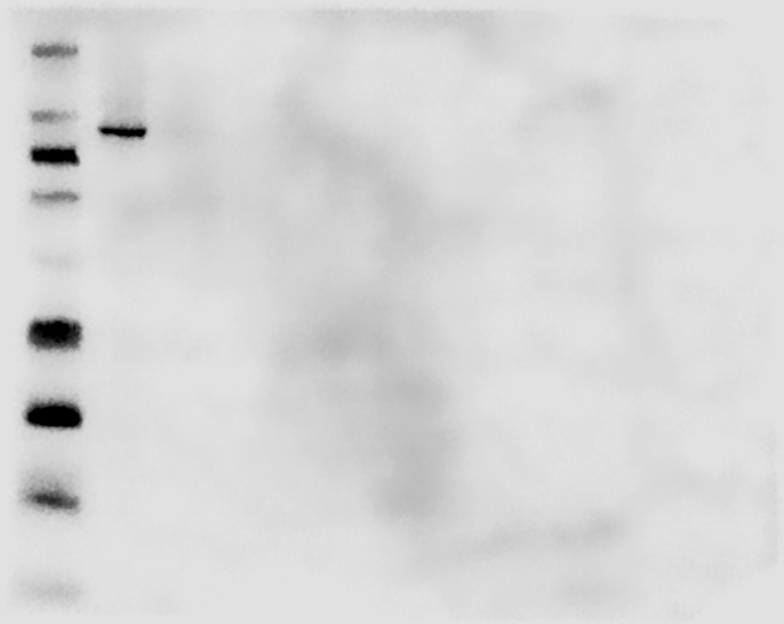

Acquisition Information

#	Image ID	Acquire Time	Channels	Integration Times	Analysis	Image Name	Comment	Image Modifications
1	0004923_01	Jan 9, 2025 12:17:53 PM	Chemi	00:59	Western	0004923_01		

Image Display Values

Channel	Color	Minimum	Maximum	K
Chemi	Gray Scale (Black on White)	0.00000727	0.000276	0

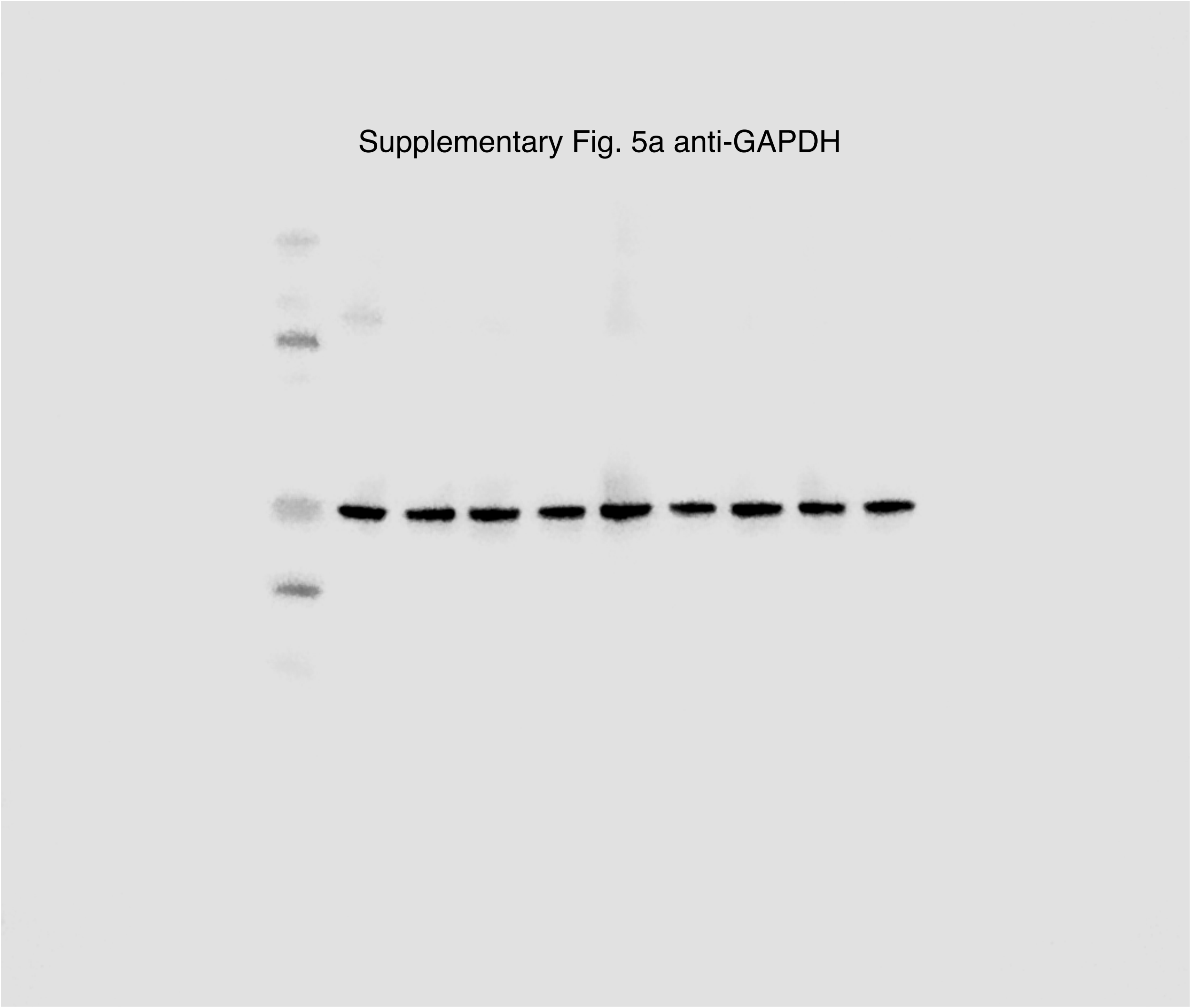

Image ID: 0004928_02
Acquire Time: Jan 14, 2025 12:17:11 PM

Acquisition Information

#	Image ID	Acquire Time	Channels	Integration Times	Analysis	Image Name	Comment
1	0004928_02	Jan 14, 2025 12:17:11 PM	Chemi	01:52	Manual	0004928_02	

Image Display Values

Channel	Color	Minimum	Maximum	K
Chemi	Gray Scale (Black on White)	0.00000364	0.000211	0

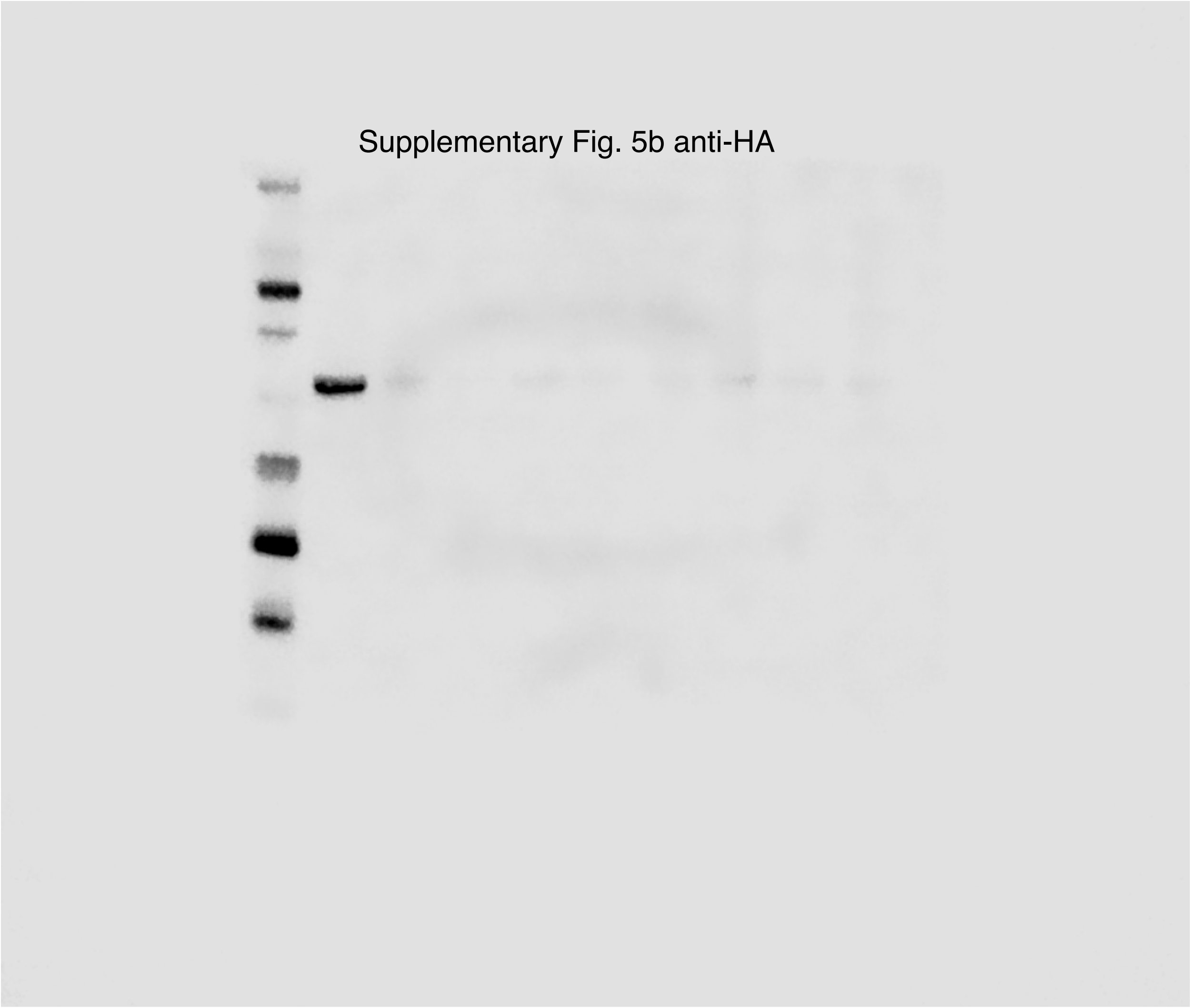

Image ID: 0004927_01
Acquire Time: Jan 14, 2025 12:13:26 PM

Acquisition Information

#	Image ID	Acquire Time	Channels	Integration Times	Analysis	Image Name	Comment	Image Modifications
1	0004927_01	Jan 14, 2025 12:13:26 PM	Chemi	01:52	Western	0004927_01		

Image Display Values

Channel	Color	Minimum	Maximum	K
Chemi	Gray Scale (Black on White)	0.00000459	0.000177	0

Supplementary Fig. 5b anti-MBNL1

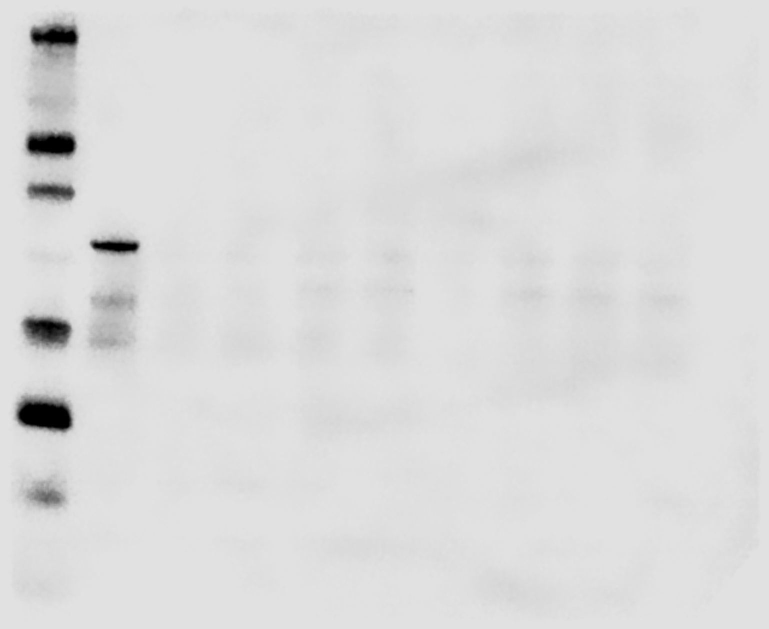

Image ID: 0004931_03
Acquire Time: Jan 15, 2025 1:30:06 PM

Acquisition Information

#	Image ID	Acquire Time	Channels	Integration Times	Analysis	Image Name	Comment
1	0004931_03	Jan 15, 2025 1:30:06 PM	Chemi	00:58	Manual	0004931_03	

Image Display Values

Channel	Color	Minimum	Maximum	K
Chemi	Gray Scale (Black on White)	0.0000327	0.000611	0

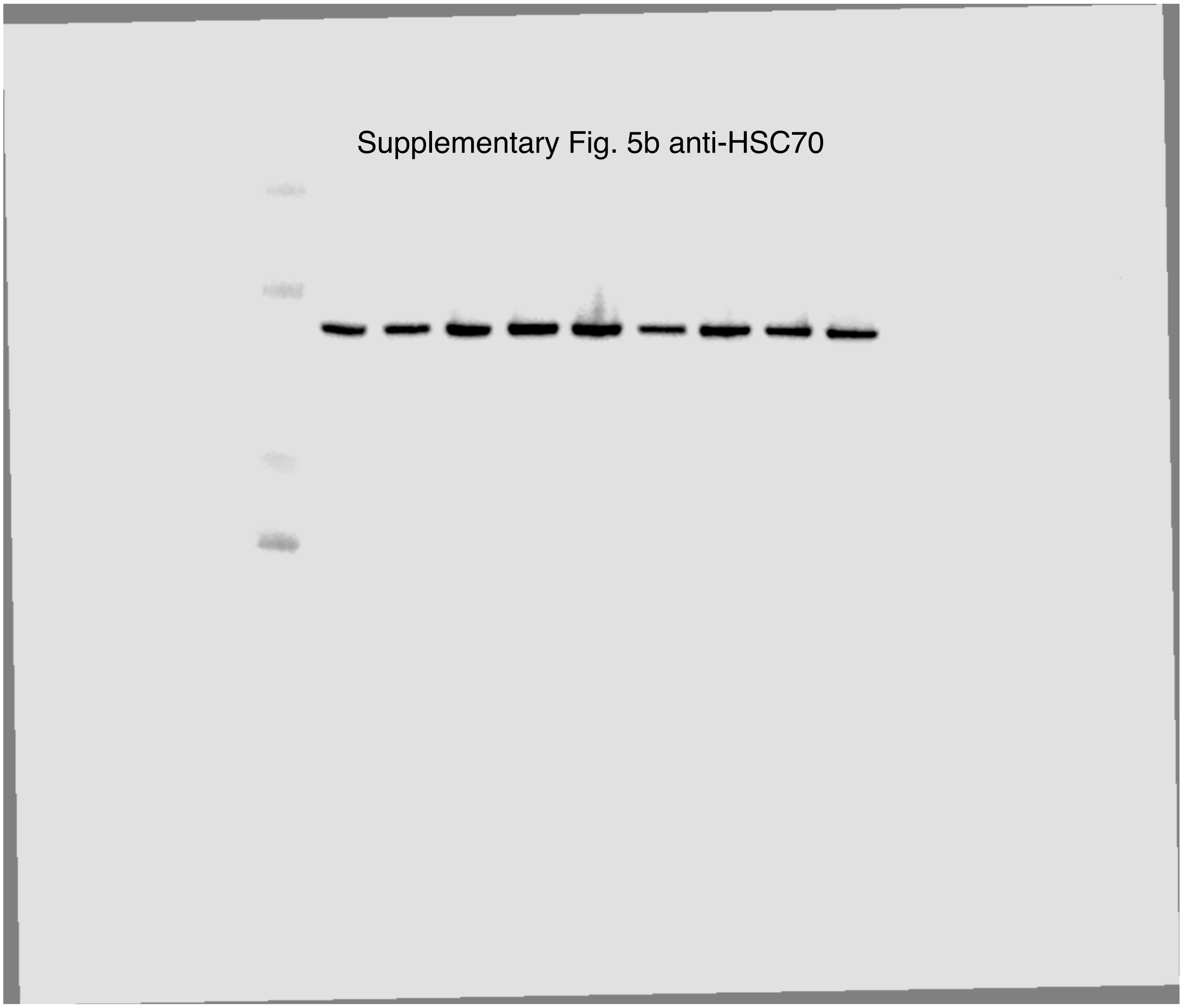

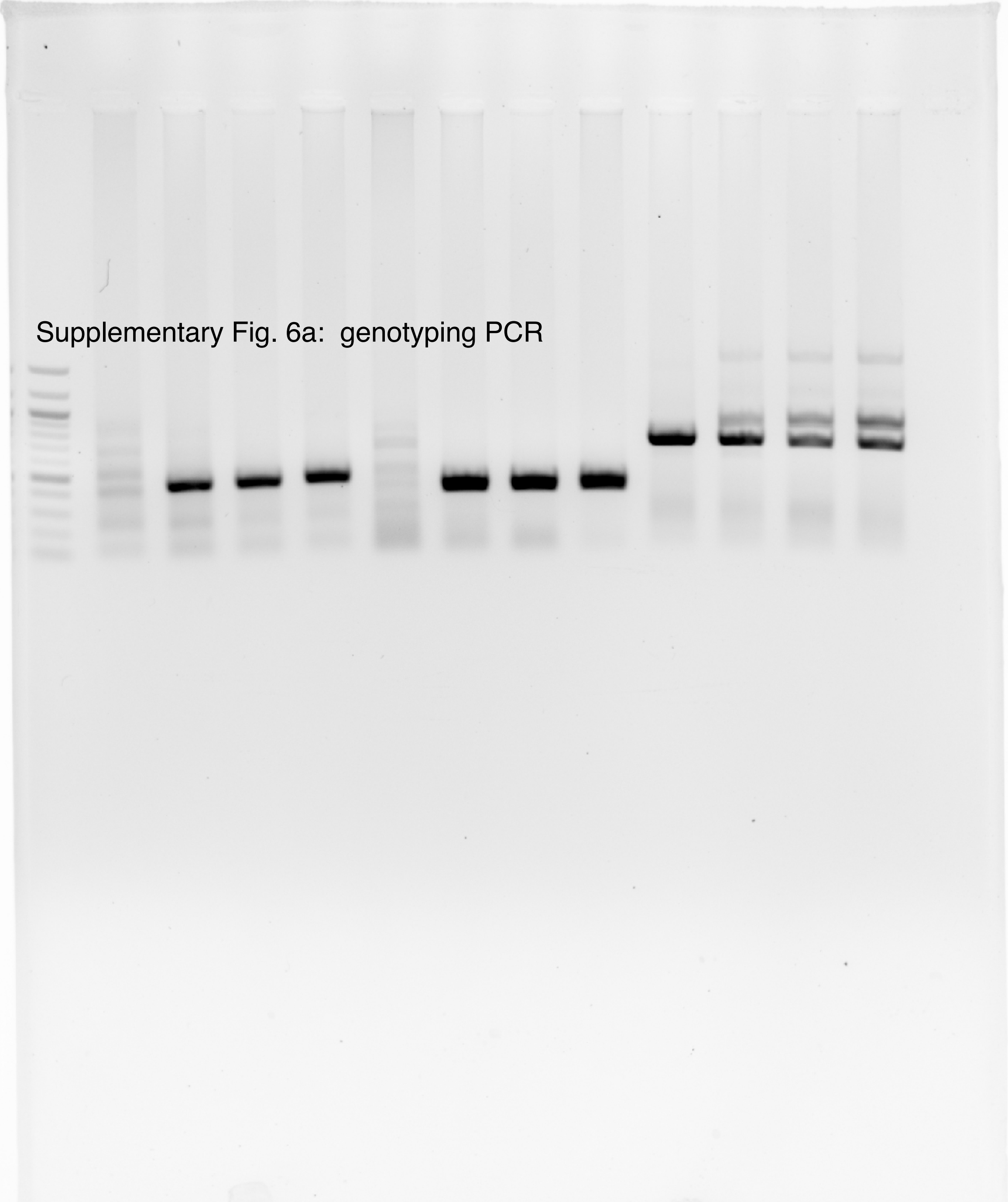
Supplementary Fig. 6a: genotyping PCR

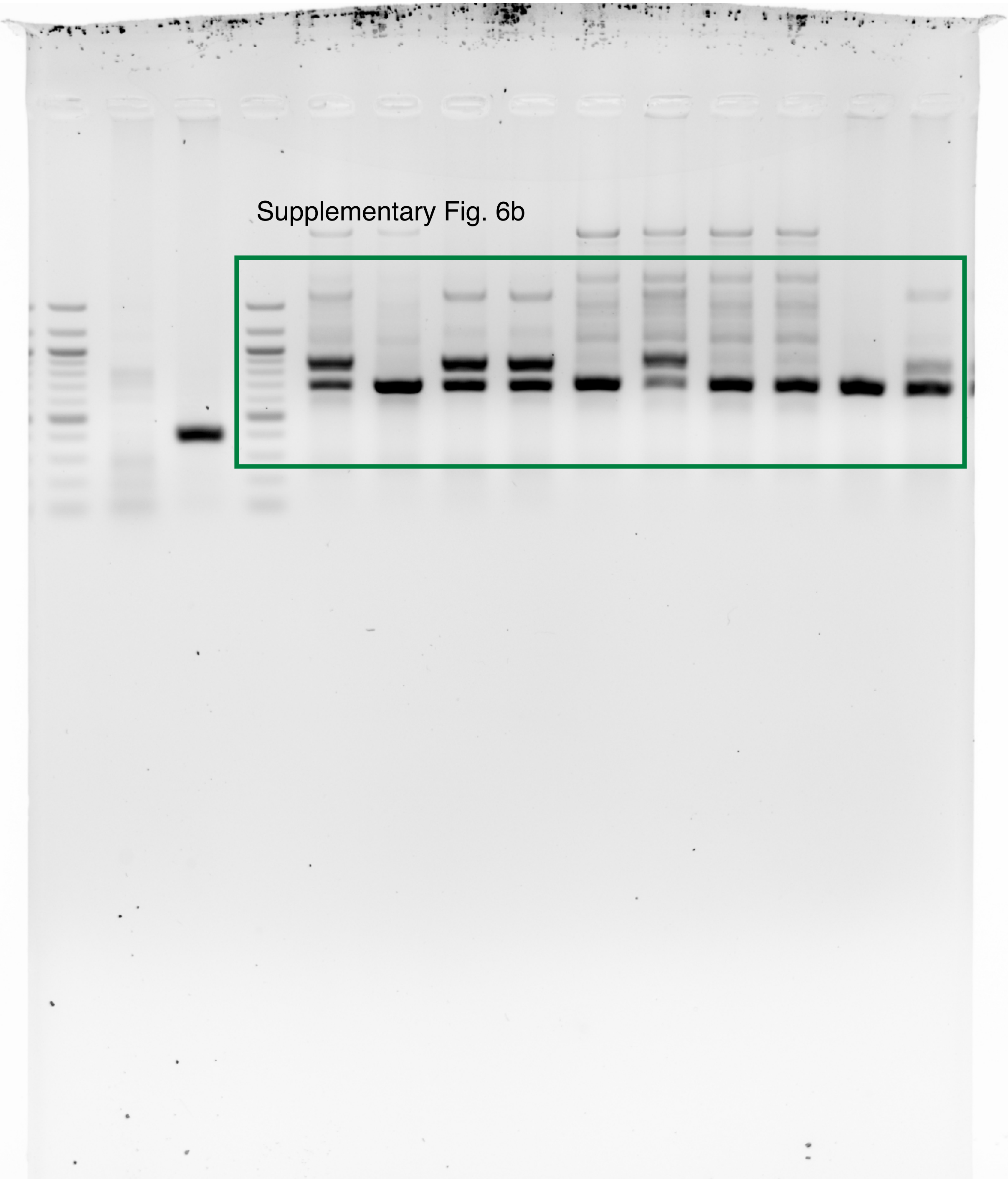
Supplementary Fig. 6b

LI-COR®

Image ID: 0004370_02
Acquire Time: Sep 19, 2024 3:50:53 PM

Acquisition Information

#	Image ID	Acquire Time	Channels	Integration Times	Analysis	Image Name	Comment
1	0004370_02	Sep 19, 2024 3:50:53 PM	Chemi	02:10	Manual	0004370_02	

Image Display Values

Channel	Color	Minimum	Maximum	K
Chemi	Gray Scale (Black on White)	0.000132	0.00775	0

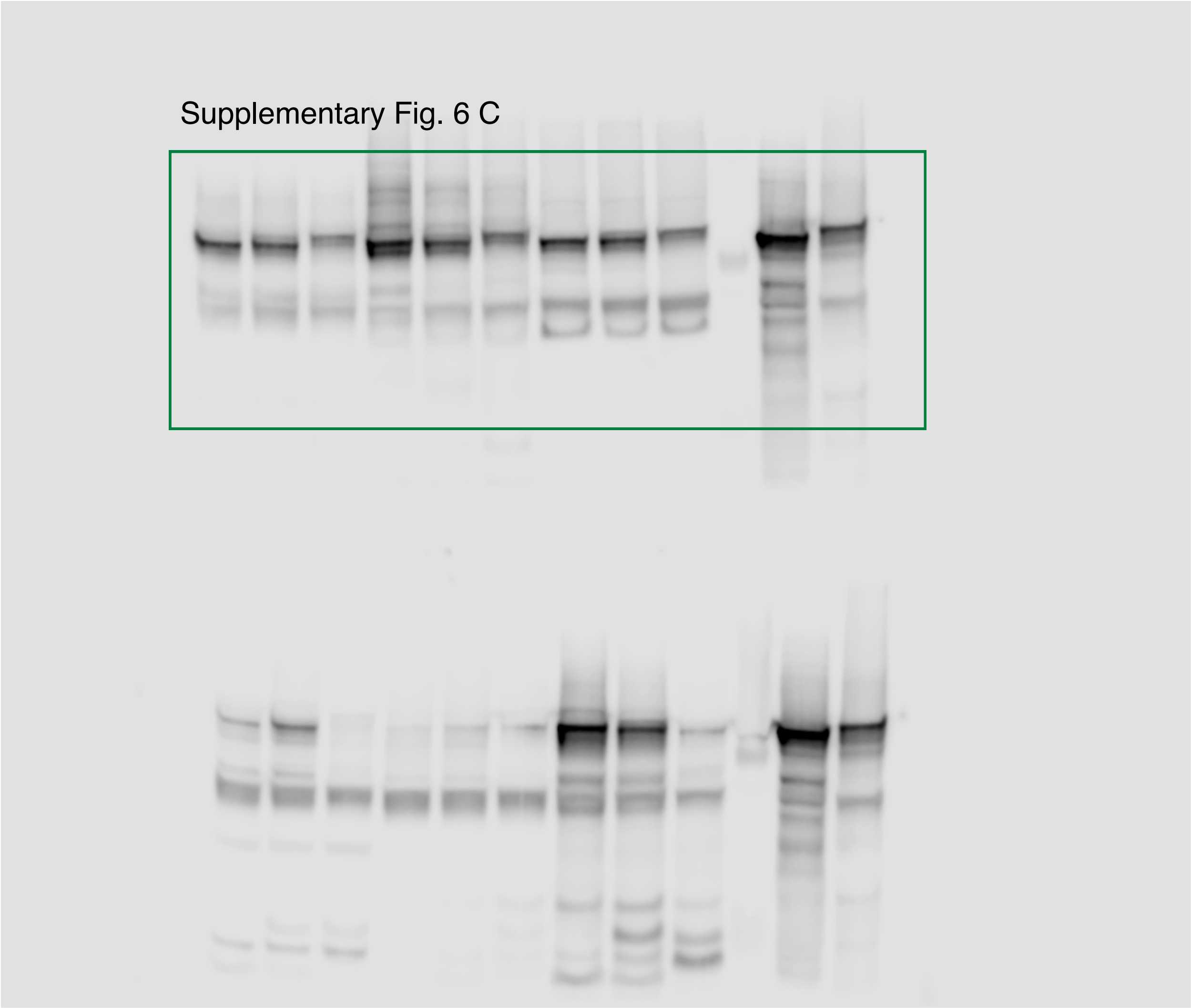

Image ID: 0004532_02
Acquire Time: Oct 18, 2024 1:19:15 PM

Acquisition Information

#	Image ID	Acquire Time	Channels	Integration Times	Analysis	Image Name	Comment
1	0004532_02	Oct 18, 2024 1:19:15 PM	Chemi	02:00	Manual	0004532_02	washout- CTCF HSP70

Image Display Values

Channel	Color	Minimum	Maximum	K
Chemi	Gray Scale (Black on White)	0.0000188	0.000242	0

Supplementary Fig. 6g Ctcf and Hsc70

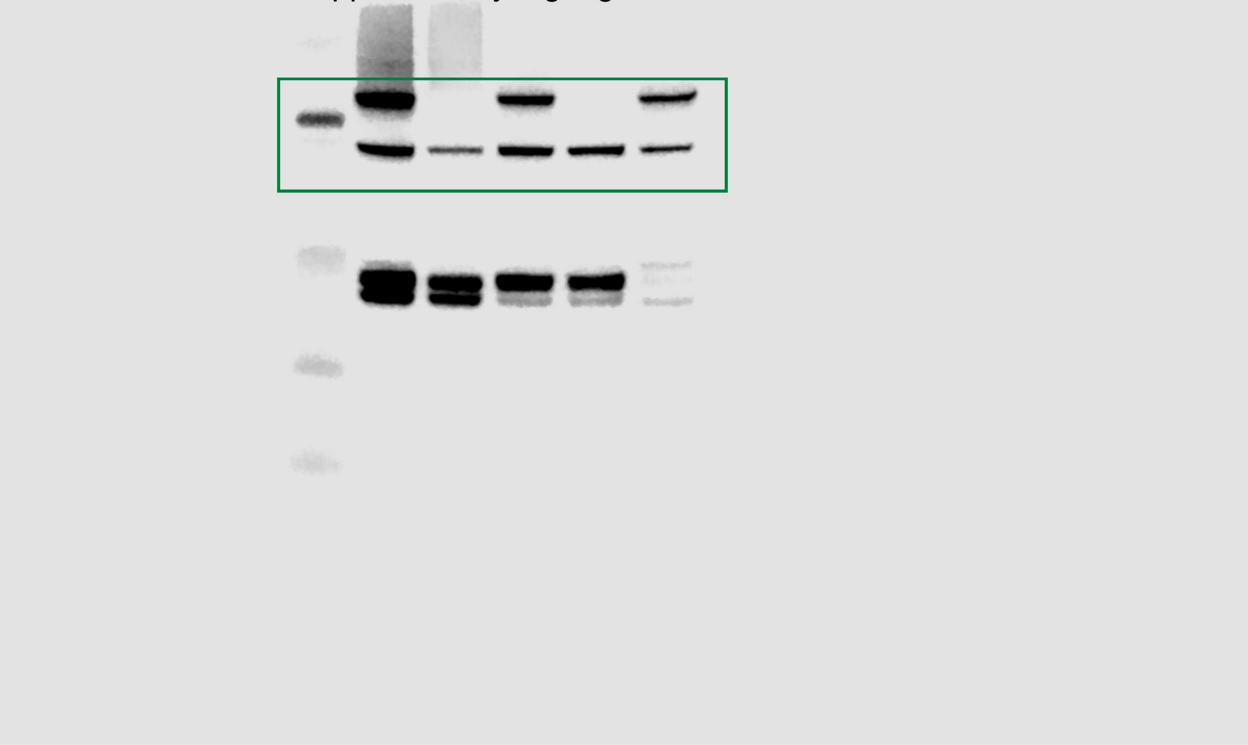

Image ID: 0004495_02
Acquire Time: Oct 16, 2024 12:57:59 PM

Acquisition Information

#	Image ID	Acquire Time	Channels	Integration Times	Analysis	Image Name	Comment
1	0004495_02	Oct 16, 2024 12:57:59 PM	Chemi	02:00	Manual	0004495_02	washout- AID

Image Display Values

Channel	Color	Minimum	Maximum	K
Chemi	Gray Scale (Black on White)	0.00000709	0.000107	0

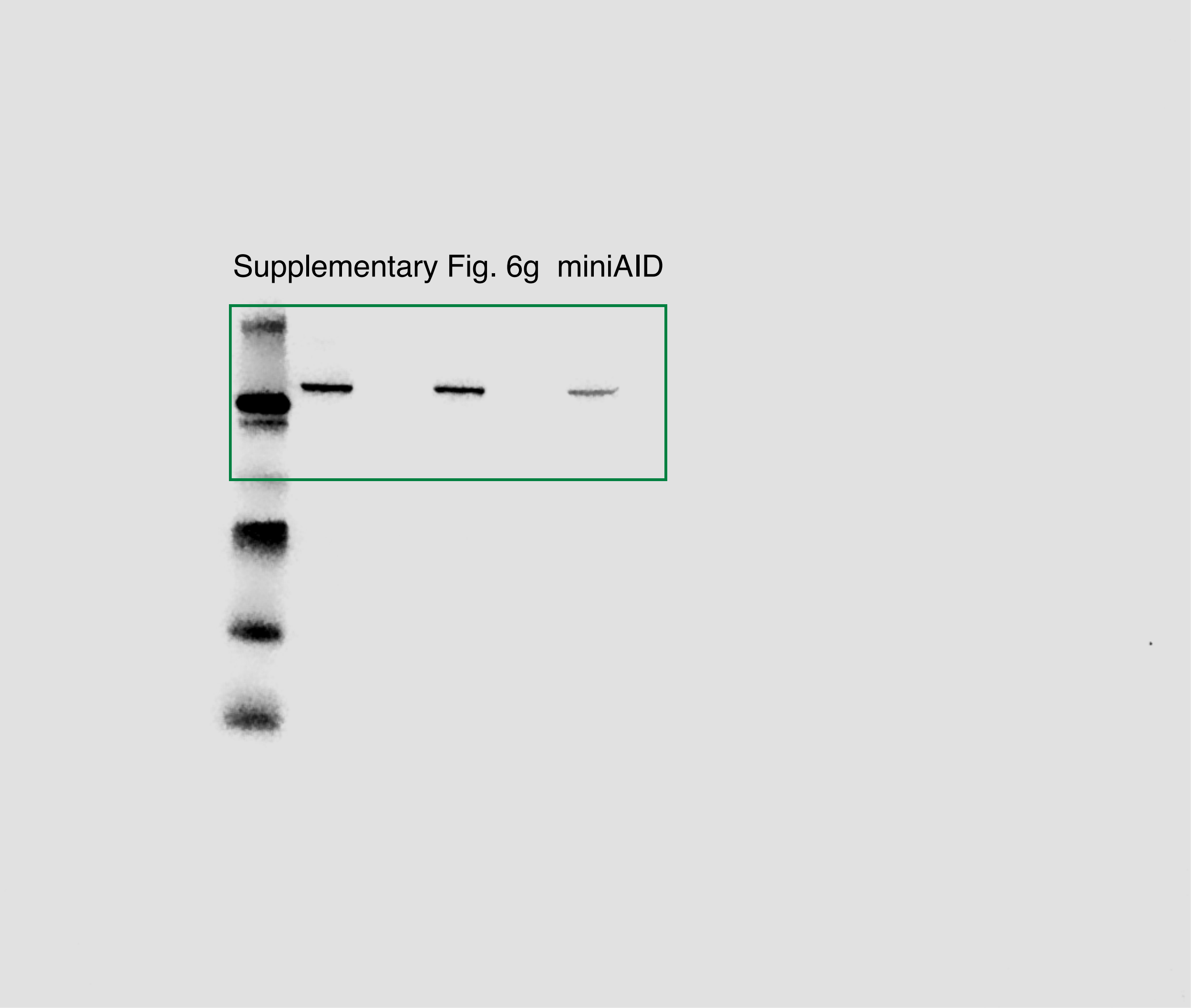

Image ID: 0004625_02
Acquire Time: Nov 1, 2024 4:18:06 PM

Acquisition Information

#	Image ID	Acquire Time	Channels	Integration Times	Analysis	Image Name	Comment
1	0004625_02	Nov 1, 2024 4:18:06 PM	Chemi	02:00	Manual	0004625_02	LT CTCF- HSC70

Image Display Values

Channel	Color	Minimum	Maximum	K
Chemi	Gray Scale (Black on White)	0.0000533	0.00334	0

Supplementary Fig. 7b Ctcf and HSC70

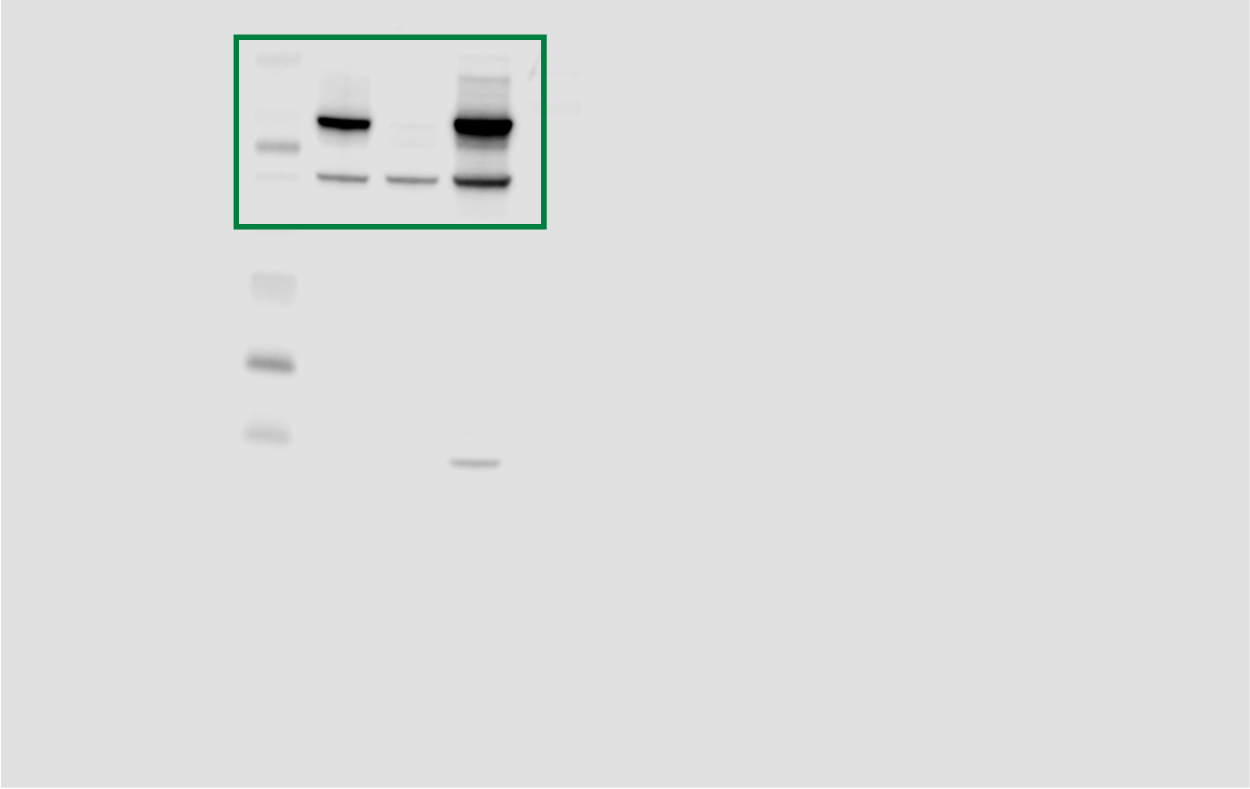

Image ID: 0005432_02
Acquire Time: May 16, 2025 4:05:31 PM

Acquisition Information

#	Image ID	Acquire Time	Channels	Integration Times	Analysis	Image Name	Comment
1	0005432_02	May 16, 2025 4:05:31 PM	Chemi	02:00	Manual	0005432_02	HSC70 for TP cells CTCF

Image Display Values

Channel	Color	Minimum	Maximum	K
Chemi	Gray Scale (Black on White)	0.0000228	0.00158	0

Supplementary Fig. 7i Ctcf and HSC70

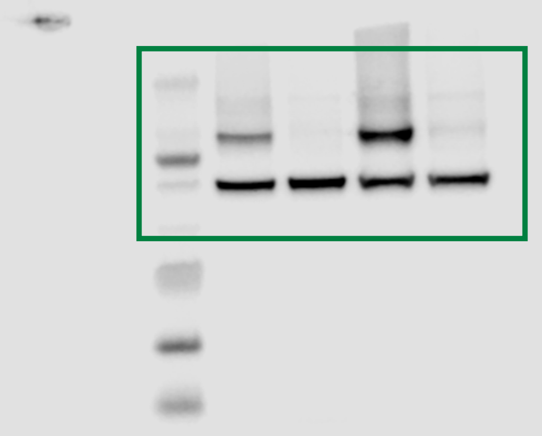

Image ID: 0005433_02
Acquire Time: May 16, 2025 4:10:08 PM

Acquisition Information

#	Image ID	Acquire Time	Channels	Integration Times	Analysis	Image Name	Comment
1	0005433_02	May 16, 2025 4:10:08 PM	Chemi	02:00	Manual	0005433_02	HSC70 for TP cells AID

Image Display Values

Channel	Color	Minimum	Maximum	K
Chemi	Gray Scale (Black on White)	0.0000157	0.000725	0

Supplementary Fig. 7i miniAID and HSC70

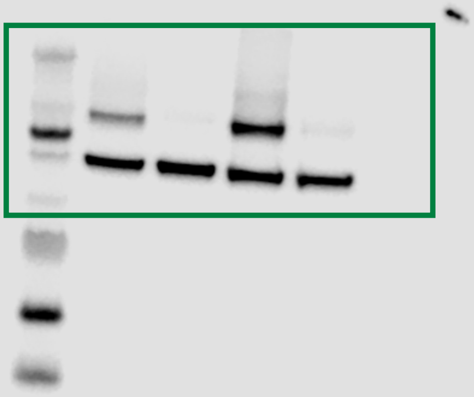

Supplement: Supplementary Information [file mmc6.pdf]
